# Time-resolved Mendelian randomization detects substantial variation in the detrimental effect of obesity throughout life

**DOI:** 10.1126/sciadv.adv0926

**Published:** 2025-10-24

**Authors:** Torgny Karlsson, Fatemeh Hadizadeh, Mathias Rask-Andersen, Daniel Schmitz, Åsa Johansson

**Affiliations:** Department of Immunology, Genetics and Pathology, Science for Life Laboratory, Uppsala University, Box 815, 75108 Uppsala, Sweden.

## Abstract

The global burden of disease attributable to obesity continues to rise. The disease incidence is substantially higher in elderly populations, but how obesity affects disease risk across a lifetime is largely unknown. To quantify the long-term temporal impact of obesity, access to large-scale longitudinal cohorts spanning many decades would typically be required. However, these longitudinal studies are rare and may be heavily biased by the presence of unaccountable confounding. Here, we develop a method—time-resolved Mendelian randomization—to estimate the cumulative effect of body mass index on disease risk at different ages. Using the UK Biobank, we find strong age-varying patterns for type 2 diabetes mellitus, coronary artery disease, and atrial fibrillation, as well as for osteoarthritis. We demonstrate that some of the most notable temporal characteristics are sex specific, while other features are shared between sexes. Analyses suggest that these features can be manifestations of primary prevention strategies.

## Teaser

Time-resolved MR untangles when, in life, obesity most strongly modifies disease risk, which facilitates better-timed preventions.

## INTRODUCTION

Obesity poses a serious hazard to human health. It is a major risk factor for numerous common diseases such as cardiometabolic conditions and osteoarthritis (OA), which are among the leading causes of mortality and disability (*1*), and the prevalence of obesity worldwide increases at an alarming rate with nearly 1 billion adults currently classified as being obese (*2*). There are also differences between sexes. The distribution of adiposity within the human body is strongly sex specific (*3*), and fat deposits in different body compartments are distinctly coupled to disease risk (*4*, *5*). However, despite the established link between obesity and higher incidence of disease, the detailed mechanisms of how obesity influences the risk in females and males over the course of a lifetime remain largely unclear. There is an urgent need for a deeper understanding of the intricate interplay between obesity and sex-specific disease development across different stages in life (*6*). Identifying changes in disease risk with increasing age would not only shed light on the temporal etiology of disease but would also enable us to determine specific windows of increased disease susceptibility and to select optimal timings for preventive interventions—timings that may not be equal for females and males, a priori. This, however, calls for a causal framework that acknowledges the dynamics of the underlying mechanisms.

**Fig. 1.**
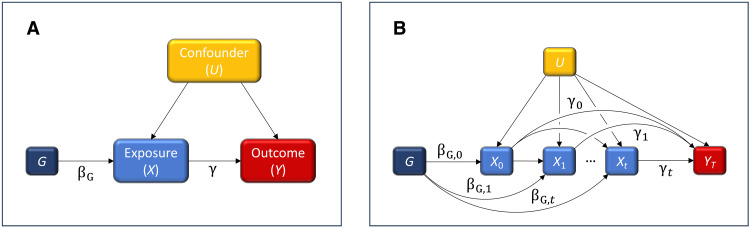
Directed acyclic graphs for time-fixed and time-dependent effects. (**A**) The causal pathways in a standard MR analysis where all effects are time invariant. The general idea behind the instrumental-variable technique is to identify a proxy variable—an instrument—associated with the exposure of interest, such that the proxy and the outcome of interest do not have any causes in common. Genetic variants would be a natural choice of instrument since the genotype is not affected by any variable determined after conception, including disease outcomes. The effect of the genetic instrument on exposure is denoted by $\beta_{G}$ , while the sought-after effect of the exposure on outcome is denoted by γ . If G is a valid instrument (see the Supplementary Materials), the effect of the instrument on outcome, η , is entirely mediated via the exposure, wherefore $\eta =\beta_{G}\gamma$ . The Wald ratio, $\eta /\beta_{G}=\gamma$ , provides an estimate of the unconfounded effect of the exposure on outcome. (**B**) The causal pathways when both the effect of the exposure on outcome and the effect of the instrument on exposure are allowed to vary at discrete time points k∈{0,1,…,t} . Here, $\beta_{G,k}$ denotes the effect of the instrument on exposure at time point k , while $\gamma_{k}$ denotes the effect of the exposure on outcome, exerted by the exposure at k . The outcome is measured at some later time point T≥t.

Mendelian randomization (MR) is an instrumental-variable technique commonly used in genetic epidemiology to test for causality and estimate unconfounded effects (*7*). In MR, an effect of the exposure on outcome can formally be estimated by the Wald ratio (Fig. 1A) (*8*), where the instrument is typically a genetic variant or a polygenic score (PGS). The identification of genetic variants to be used as instrumental variables is nowadays made with little effort, due to easily accessible summary statistics from large genome-wide association studies (GWASs) (*9*), and MR has become a standard method for causal inference in epidemiological research (*10*). Since an individual’s genotype is fixed at conception, a genetic predisposition to some specific phenotypic expression, e.g., to high body mass index (BMI), denotes a lifelong exposure. A phenotypic effect that is estimated using genetic variants as instrument would therefore be cumulative in nature due to the increasing exposure time with age. In a standard MR analysis, however, all relations are taken as time fixed (Fig. 1A). This includes the effect of the instrument on exposure that must not vary with age in standard MR to obtain interpretable causal estimates (*11*). However, for many phenotypes, including BMI, this assumption has been questioned (*12*–*15*). Instead, both the instrument’s effect on exposure and the exposure’s effect on outcome may commonly depend on time (Fig. 1B).

Few attempts have been made to incorporate time dependence in MR. Statistical tests for binary outcomes have been developed that allow for the effect of the genetic instrument on exposure to vary with time (*16*), while the method assumes a time-fixed effect of the exposure on outcome. More recently, multivariable MR has been proposed as a method for estimating the direct effect of an exposure exerted at some early point in life, accounting for the mediating effect of the exposure exerted at a later time point, on an outcome measured later in life (*17*). A study applying this method to coronary artery disease (CAD) and type 2 diabetes mellitus (T2DM) found childhood obesity to have no significant direct causal effect on these diseases later in life when adjusting for adulthood obesity (*18*). However, the method is sensitive to model misspecification, as it assumes that causality is exerted only at discrete, predefined time points (*19*). Average causal effects of a time-varying exposure can be estimated from longitudinal data with structural mean models (*20*) and structural nested cumulative failure time models (*21*), using *g*-estimation. However, the failure time models rely on the rare outcome approximation, and the instrument effect on exposure is typically assumed to be time fixed, although time-dependent instruments could, in theory, be handled for the failure time models (*21*).

To address these limitations, we developed an MR approach—time-resolved MR—where the assumptions of time invariance were relaxed (Fig. 1B). The method uses time-to-event data on disease outcomes to estimate time-dependent effects. Here, these effects represent susceptibility to a lifelong exposure to a risk factor at different ages. Time-to-event data are typically collected from linked records of in- and outpatient health registers, which often attain very high temporal resolution. To fully exploit the temporal information in these registers, we used Aalen’s additive hazard model (*22*), which also handles common outcomes. This model has several desirable properties (see Materials and Methods) and naturally allows for time-varying effects—in terms of hazard rate differences—to be estimated nonparametrically. The additivity also simplifies certain technical operations, such as integration, used to express the accumulated effect over the life course (see Materials and Methods). The cumulative effects considered in this study denote the total risk of developing disease at a given age, by being exposed to a one-unit higher level of the risk factor over the entire life course, up to this age (*11*). With this definition of “lifetime effect,” there is no need for the liability of the genetic instrument to vary across the life course (*23*), as, e.g., in multivariable MR (*17*). On the other hand, we do not require the instrument effect on the risk factor to be time invariant, and this assumption is relaxed here.

To identify critical periods in life during which a lifelong exposure most markedly changes the current risk of disease onset, we further developed a nonparametric technique to estimate effect changes averaged over a given time period (see Materials and Methods). We applied time-resolved MR to identify time-dependent effects of a lifelong exposure to high BMI on incident CAD, T2DM, atrial fibrillation (AF), and OA in males and females. These four diseases exhibit a large diversity in pathophysiology, with obesity as a common risk factor.

## RESULTS

### Overview of the time-resolved MR approach

As a basis for the discussion, let us be slightly more formal and define two types of hazard rates. The (accumulated) hazard rate usually modeled in survival analysis denotes the total rate of events at age t=T years, conditional on survival until T years. This rate is directly linked to the observed number of incident cases at T in a population. In contrast, the momentaneous hazard rate is defined as the change in rate of events per unit time at age t≤T. Since it denotes a change in the hazard rate, it is only indirectly linked to the observed number of incident cases at T and may be interpreted as a latent outcome.

Next, we define the expression “sustained exposure” to represent a unit higher value in some continuous exposure variable (e.g., BMI) during the entire time from conception at t=0 up to time t=T . Furthermore, let the cumulative effect of a sustained exposure, Γ=Γ(T) at age T years, be defined as the difference in hazard rates at time T between two exposure trajectories for which the higher trajectory is one unit higher in value during the entire time period t=[0,T] , as compared to the lower trajectory (see Materials and Methods). We will also refer to this cumulative effect as the life-course effect, as it denotes the accumulated effect exerted by the exposure during the life course up to time T . Similarly, let the momentaneous effect of the exposure at time t , γ=γ(t) , be defined as the corresponding difference in momentaneous hazard rates underlying the latent outcome at time t . Hence, γ(t) represents the net effect of one additional year of exposure at age t≤T years, conditional on exposure up to t . In this capacity, the momentaneous effect describes the causal effect exerted by the exposure at a specific moment in time. Here, γ will also be interpreted as the gradient of the cumulative effect Γ.

The derivation of the life-course effect Γ(T) using an instrument that is allowed to be time dependent is outlined in Materials and Methods. Briefly, since the instrument effect on the outcome, here denoted by Η=Η(T) , is typically estimated using observed time-to-event data, Η will represent an effect accumulated over the course of life, similar to Γ . If so, then Γ cannot directly be retrieved by taking the usual Wald ratio, even if the instrument effect on exposure, here denoted by $\beta_{G}=\beta_{G}\left(T\right)$ , is known. That is, $\Gamma \left(T\right)\neq Η\left(T\right)/\beta_{G}\left(T\right)$ . The nonequality arises because Η(T) at time T depends on $\beta_{G}\left(t\right)$ at all past time points t≤T , as a result of the accumulation of mediating effects through the exposure over the life course. This dependence can neither adequately be removed by simply dividing by $\beta_{G}$ at a single time point T nor by dividing by some time average of $\beta_{G}$ (see Fig. 1). Instead, we propose to remove the dependence on $\beta_{G}$ by operating on the time gradient of Η , i.e., $\dot{Η}\left(t\right)=\eta \left(t\right)=\gamma \left(t\right)\beta_{G}\left(t\right)$ , which corresponds to the mediating effect at the single time point t . Here, we made the simplifying assumption that a prompt change in exposure, i.e., a change exerted over a short time interval, results in an instant and irreversible change in the latent outcome. A generalized model relaxing this assumption is briefly discussed in the Supplementary Materials. When the momentaneous effects, $\gamma \left(t\right)=\eta \left(t\right)/\beta_{G}\left(t\right)$ , have been estimated, γ may be integrated over time to retrieve an estimate of Γ(T).

### Simulations of a time-dependent effect on disease with a time-dependent instrument

To validate our method, we performed several simulations, as described in detail in the Supplementary Materials. Briefly, we generated time-to-event data under a model where both the exposure and the genetic instrument effect varied over time. The causal effect of the exposure on the latent disease process was assumed to follow a power law in time, such that $\gamma \left(t\right)\propto t^{4}$ . This corresponds to a cumulative effect Γ(T) at time T , which is proportional to $T^{5}$ . The exposure distribution was modeled to resemble the distribution of BMI in UK Biobank (UKB) and was generated using a dichotomous, binomially distributed genetic instrument, a γ-distributed confounder with a time-dependent coefficient, and an additional time-dependent covariate (see the Supplementary Materials). The latent outcome was generated in a similar way but with an exposure term instead of an instrument and with different time-dependent coefficients. The time dependences for all simulated variables are given in table S1. Disease onset was modeled using a negative binomial process based on a transformed cumulative hazard. The instrument effect on exposure was estimated using either a misspecified or a correctly specified linear regression model (see the Supplementary Materials for details), while the instrument effect on outcome was estimated using Aalen’s additive hazard model.

**Fig. 2.**
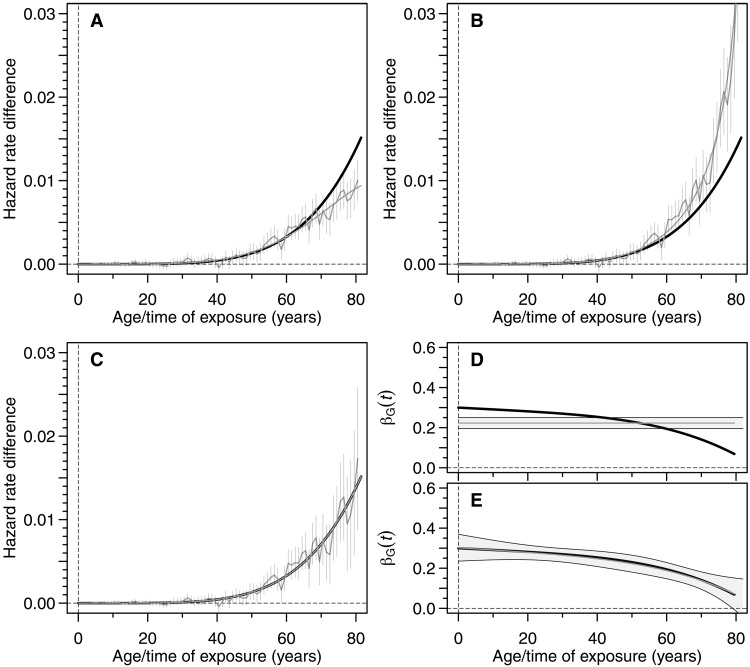
Simulation of time-dependent effect with a time-dependent instrument. The cumulative effect of the exposure on outcome is assumed to follow a power law in time $\propto T^{5}$ , while the time-dependent effect of the instrument on exposure is a quartic polynomial of the form: $\beta_{G}\left(t\right)=0.3-9\times 10^{-4}t-4\times 10^{-9}t^{4}$ , t∈[0,80] . The prevalence of the outcome is 15% in the population, with age ≤80 years. (**A**) Biased estimation [gray; connected points with 95% confidence interval (CI)] of the nonlinear ( $\propto T^{5}$ ) cumulative effect (black thick line), adopting a naïve use of the Wald ratio (see the Supplementary Materials). Here, the time-dependent instrument effect $\beta_{G}\left(t\right)$ is approximated by a constant, time-averaged effect. The biased, underlying effect is denoted by the gray curve. (**B**) Biased estimation (gray; connected points with 95% CI) of the cumulative effect (black thick line), adopting a naïve use of the Wald ratio. Here, we have assumed the proper time dependence $\beta_{G}\left(T\right)$ of the instrument effect, but we have not accounted for the fact that the instrument effect on outcome at any time T depends on $\beta_{G}\left(t\right)$ at all past time points t≤T . The biased effect is denoted by the gray curve. (**C**) Unbiased estimation (gray; connected points with 95% CI) of the cumulative effect (black thick line) using time-resolved MR. No theoretical bias is present (gray curve). (**D**) Estimated main (constant) effect of instrument on exposure is denoted by the gray thin line with corresponding 95% CI. This estimate is used in the calculations presented in (A). The black thick curve denotes the true time dependence (quartic polynomial) of the instrument. (**E**) Estimated time-dependent effect of instrument on exposure assuming the correct quartic dependence is denoted by the gray thin curve with corresponding 95% CI. This estimate is used in the calculations presented in (B) and (C). The black thick curve denotes the true time dependence (quartic polynomial) of the instrument.

We compared three approaches in the simulations: two naïve implementations of the Wald ratio, with either time-fixed or time-varying instrument effects, and our proposed time-resolved MR method. In the first naïve method, the cumulative effect was estimated by dividing the instrument effect on outcome estimated from Aalen’s model by a constant, time-averaged estimate of $\beta_{G}$ estimated from the misspecified model. In the second naïve method, the instrument effect on outcome at each time T was divided by the effect $\beta_{G}\left(T\right)$ estimated from the correctly specified model (see the Supplementary Materials). The naïve methods produced biased estimates of the time-dependent exposure effect, particularly at older ages, due to their inability to adequately separate the time dependences of exposure and instrument. In contrast, time-resolved MR accurately recovered the true time-varying causal effect across the entire age range (Fig. 2).

Further simulations (fig. S4) also confirmed the robustness of time-resolved MR under varying conditions, including different disease prevalences (3 and 30%), increasing instrument strength with age, and protective effects that increased in strength over time. In all scenarios, time-resolved MR consistently outperformed the naïve methods, which often showed substantial bias, especially later in life. This shows that time-resolved MR is a reliable and unbiased method for estimating time-dependent life-course effects, even in the presence of time-varying instruments.

In the applied analyses using real world data (see below), we used a PGS for BMI as an instrument to ensure that the first MR assumption, the relevance condition (see the Supplementary Materials), was satisfied across the entire lifespan. To verify that a PGS, defined as the weighted sum of single-nucleotide polymorphisms (SNPs), is also a valid time-dependent instrument, we performed an additional simulation in which we assumed the exposure to be described by three independent genetic variants with unique and highly diverse time-dependent effects (see the Supplementary Materials and fig. S5A). For simplicity, we defined the corresponding PGS as the unweighted sum of these three SNPs. We estimated the time-dependent effects of this PGS on the exposure (fig. S5A) and on outcome and performed a time-resolved MR analysis. The simulation showed that time-resolved MR accurately estimates the effect of the exposure on outcome with the PGS as a valid instrument given its specific time dependence (fig. S5B), despite the fact that it is generated from individual genetic variants with distinctly different time-dependent effects on exposure.

### Age-dependent effect of PGS on BMI

We applied time-resolved MR to identify time-dependent effects of a sustained high BMI on age at disease onset, with age as primary timescale (Table 1). The analysis was performed in UKB adopting a one-sample design, and a PGS for BMI was used as instrument. To avoid an overfitted PGS (*24*), we performed a separate GWAS for BMI in two independent, randomly selected subsamples ( $N_{A}=N_{B}=180,953$ ) of UKB, hereafter referred to as subsamples A and B . Genome-wide significant ( $P=5\times 10^{-8}$ ) SNPs were identified, and their effect sizes were extracted from each GWAS to be used as weights. We identified, in total 122 independent lead SNPs in subsample A and 106 independent lead SNPs in subsample B (data S1). Of these, 11 SNPs were identified in both subsamples, while 36 of the remaining SNPs were in high linkage disequilibrium ( $R^{2}>0.80$ ) and 52 SNPs were in low to moderate linkage disequilibrium ( $0.05<R^{2}<0.80$ ). For each subsample, a PGS was computed using the SNPs and weights identified in the other subsample (see Materials and Methods). The PGS for each subsample was thus computed independently from the data in which the SNPs selected for the PGS were identified. The two PGSs were then standardized within each subsample before the subsamples were remerged into a single sample.

**Table 1.** Descriptive statistics of the exposure and the four outcome diseases.

		Females	Males	Sex-combined
Number of participants (%)		194,770 (53.8)	167,136 (46.2)	361,906 (100.0)
Age at baseline assessment, median (first to third quartile)		58 (51–63)	59 (51–64)	58 (51–63)
Age at end of follow-up, median (first to third quartile)^*^		70 (62–75)	70 (62–75)	70 (62–75)
BMI at baseline assessment, means ± SD		27.03 ± 5.14	27.84 ± 4.23	27.41 ± 4.76
T2DM	Events ≤76 years/controls (%)^†^	10,427/184,343 (5.4)	16,287/150,847 (9.7)	26,714/335,190 (7.4)
	Percent of events ≤76 years^‡^	95.1	95.6	95.4
AF	Events ≤76 years/controls (%)^†^	8,379/186,390 (4.3)	15,565/151,570 (9.3)	23,944/337,960 (6.6)
	Percent of events ≤76 years^‡^	88.4	90.7	89.9
CAD	Events ≤76 years/controls (%)^†^	13,807/180,962 (7.1)	27,399/139,736 (16.4)	41,206/320,698 (11.4)
	Percent of events ≤76 years^‡^	94.1	95.7	95.2
OA	Events ≤76 years/controls (%)^†^	52,329/142,439 (26.9)	36,241/130,894 (21.7)	88,570/273,333 (24.5)
	Percent of events ≤76 years^‡^	97.2	96.7	97.0

* Age at end of follow-up was defined as the age on 31 December 2020 or age at death, whichever came first.

† Number of first-time events that occurred in participants with age ≤76 years. Controls denote those individuals who either did not have an event during this time period ( ≤76 years) or who did not have an event before end of follow-up, while the values within parentheses denote the disease prevalence at this time point. The cutoff age of 76 years was chosen to avoid spurious boundary effects in Aalen’s model, as well as low-number statistics present in the oldest age bins, above 76 years. Note that the number of events and controls do not always add up to the total number of participants in each stratum (females, males, and sex-combined) due to a small number of undefined event dates specific for each disease.

‡ Values denote the fraction of events that occurred at an age ≤76 years relative to all events in the specific stratum of UKB (females, males, and sex-combined) registered before end of follow-up.

**Fig. 3.**
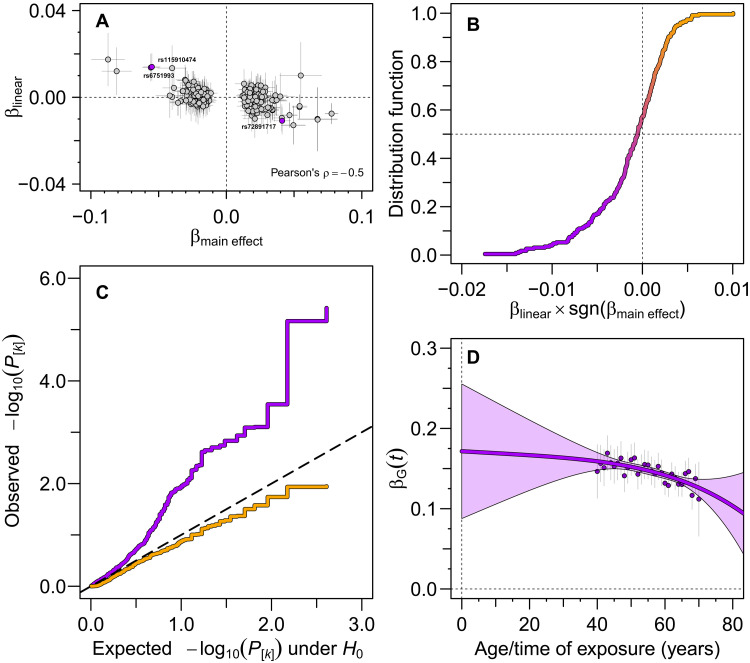
An age-dependent component of the genetic effect on BMI. Many BMI-increasing alleles show evidence of a diminishing effect with increasing age. (**A**) Estimated effects of the linear interaction with age ( $\beta_{\text{linear}}$ ) against main, time-fixed effects ( $\beta_{\text{main effect}}$ ) for the BMI-associated lead SNPs (data S1) identified in the two subsamples of UKB (see Materials and Methods). While only three SNPs (filled violet circles) show a significant time dependence [false discovery rate (FDR) < 0.05], a significant negative correlation ( ρ=−0.5 ; 95% CI, −0.6 to −0.4 ) is observed between the main and the linear interaction effects. (**B**) Empirical distribution function of the linear interaction effect estimates, aligned with the BMI-increasing effect allele. The fraction of negative interaction effects is larger (57% versus 43%), and the tail of negative interactions also extends toward more extreme values ( −0.0174 versus +0.0100 ), as compared to that of positive interaction effects. Here, $\text{sgn}\left(\beta_{\text{main effect}}\right)$ denotes the sign of $\beta_{\text{main effect}}$ . (**C**) Quantile-quantile plot for one-tailed *P* values of the linear interaction effect estimates. The violet curve denotes the *P* values in decreasing order for a left-tailed test with alternative hypothesis $H_{1}:\beta_{\text{linear}}\times \text{sgn}\left(\beta_{\text{main effect}}\right)<0$ , while the amber-yellow curve denotes the corresponding *P* values in decreasing order for a right-tailed test with alternative hypothesis $H_{1}:\beta_{\text{linear}}\times \text{sgn}\left(\beta_{\text{main effect}}\right)>0$ . (**D**) The age-dependent effect $\beta_{G}\left(t\right)$ of the BMI-PGS on BMI (violet curve) using a quartic fit to the data (see Materials and Methods). The 95% CI of the fit is denoted by the light-violet area. Dark-violet filled circles denote main, time-fixed effect estimates stratified on age.

To underscore the importance of considering time-dependent instrument effects in MR, we sought to identify any potential time-varying component in the instrument effect on BMI. As illustrated in Fig. 3 (A to D), clear evidence of a decreasing effect with increasing age was found both for the individual lead SNPs and for the BMI-PGS instrument generated from these SNPs (however, see also the “Impact of selection into UKB” section). For the individual lead SNPs, the number of low *P* values corresponding to negative SNP×age interaction effects is enriched (Fig. 3C), which suggests that an appreciable number of the observed negative effects are truly negative. In contrast, the number of low *P* values that corresponds to positive interaction effects appears to be consistent with numbers expected from random variation alone. The BMI-PGS was found to have a significant time-dependent component (*P* = 1.2 × 10^−7^ for $\beta_{\text{linear}}$ ), where the effect on BMI decreases with increasing age (Fig. 3D). The corresponding time-dependent effects of the disease-specific PGSs on *z*-transformed BMI are depicted in fig. S6.

### Age-dependent, sex-combined effect of sustained high BMI on incident disease

Before performing the time-resolved MR analyses, we computed disease-specific PGSs to be used as instruments. We applied Steiger filtering (*25*) separately in each subsample to exclude invalid genetic variants that did not significantly explain more variance in BMI than in the specific disease outcome. Only a small number of SNPs were generally excluded for each disease (see data S1). After the Steiger filtering step, we computed a BMI-PGS unique to each disease, as described in Materials and Methods.

**Fig. 4.**
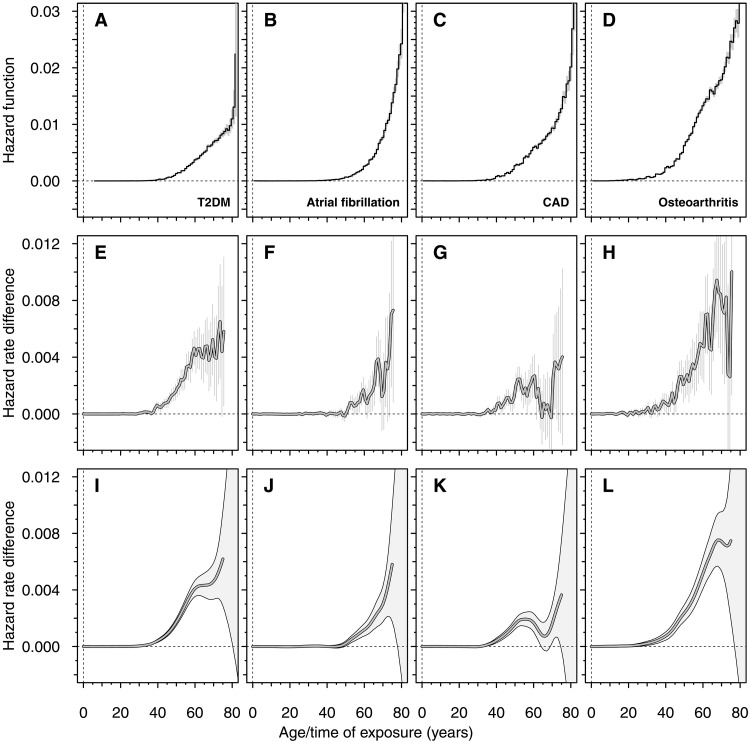
Time-resolved, cumulative effects of BMI on four complex diseases in all individuals. Each column shows the results for a specific disease. The four top panels show crude hazard functions of (**A**) T2DM, (**B**) AF, (**C**) CAD, and (**D**) OA. The middle panels show the time-dependent differences in hazard rates (per year) having one SD higher BMI during the life course, up to a given age, for (**E**) T2DM, (**F**) AF, (**G**) CAD, and (**H**) OA. Cumulative effects are numerically integrated over time using the trapezoidal rule (see Materials and Methods). Corresponding time-series trends of the cumulative effects, estimated using Hodrick-Prescott filtering with λ=50 (see Materials and Methods), are presented in the bottom panels for (**I**) T2DM, (**J**) AF, (**K**) CAD, and (**L**) OA. Parametric bootstrap with 10,000 bootstrap samples was used to calculate the 95% CIs of the trends. Similar to AF, OA shows an increase in effect over time, at least up to about 65 to 70 years of age, after which the temporal progression of the trend becomes uncertain. The increasing trend starts later for AF than for OA, but the rate of increase is steeper for AF (fig. S7). Around 60 years of age, the effect of BMI on T2DM levels off temporarily at about 0.0045 ${\text{year}}^{-1}$ , while the effect on CAD exhibits a distinct trough starting already around the age of 50 years (fig. S8).

Similar to the overall burden of each of the four diseases investigated (Fig. 4, A to D), the cumulative effect of BMI on disease rate, as estimated by time-resolved MR, generally increases with increasing age (Fig. 4, E to L). However, this increase in effect is not always monotonic, and several distinct features among the diseases are noted. Although AF and OA both exhibit predominantly increasing trends with age (Fig. 4, J and L, and fig. S7, A and B), these trends differ in several aspects. While BMI becomes a nonnegligible risk factor for OA more than 20 years earlier than for AF, the rate at which the increase in effect occurs is considerably higher for AF than for OA (figs. S7C and S8). Hence, BMI rapidly becomes an appreciable risk factor for AF but only in the old population.

The life-course effects of BMI on T2DM and CAD were found to be complex and multifaceted. After an initial increase, the effect of BMI on T2DM appears to reach a temporary plateau around 60 to 70 years of age (Fig. 4, E and I). At around the same age range, the effect on CAD shows a significant decrease to near null before the effect increases again (Fig. 4, G and K, and fig. S8). It is unclear what would be the origin of such a local minimum—a trough—in effect. To rule out the possibility that these features are artifacts of changes in participation rates into UKB postretirement, we performed an analysis in which we only included participants who had not yet reached retirement age at assessment. The results show that these features are also present in the subsample of preretirement participants and suggest that any potential changes in participation around retirement cannot explain the troughs and features observed in the trends for CAD and T2DM (fig. S9).

### Age-dependent effect of sustained high BMI on incident CAD in individuals on lipid-lowering treatment

The trough observed in the trend of the life-course effect on CAD could potentially be a mediating result of preventive intervention, such as the introduction of statins (*26*) and other lipid-lowering medications, which are prescribed more frequently to overweight individuals in the UKB [relative risk, 1.40; 95% confidence interval (CI), 1.39 to 1.41]. We therefore performed a secondary analysis, where we first removed participants with a CAD event before assessment. This was done to reduce the risk of including participants taking lipid-lowering treatment in response to a CAD event and to ensure that all individuals who were taking medication at assessment were diagnosis free. Then, we ran two analyses for CAD: In the first analysis, we followed participants who said that they were taking lipid-lowering treatment at assessment. In the second reference analysis, we followed participants who did not take lipid-lowering treatment or antihypertensives at assessment. While the reference analysis showed a considerably weakened trough, the first analysis showed a distinct trough appearing around 65 to 70 years (fig. S10), in the same age span as the trough in Fig. 4K. These results suggest that primary preventive treatment can potentially explain the presence of some troughs in certain diseases, such as CAD.

### Age-dependent effect of sustained high systolic blood pressure on incident AF and CAD

In a secondary analysis, we further estimated the corresponding life-course effects of systolic blood pressure (SBP) on AF and on CAD to ascertain whether similar troughs are present in other exposure-outcome relations and whether prevention may pose a plausible explanation for the existence of troughs. Elevated blood pressure is a strong risk factor for AF (*27*, *28*). Individuals in their midlife who are treated for hypertension with antihypertensive medication are likely to lower their SBP, which may then temporarily mitigate the risk of AF. If so, then we would expect to find a similar trough in the trend of the life-course effect of SBP on AF. A moderate trough was also observed for AF (fig. S11). Contrarily, no obvious trough in the trend of the effect of SBP on CAD was observed. In addition, we did not detect a trough in the effect of BMI on AF (e.g., fig. S11), which may be consistent with statin treatment not having a strong effect on AF risk (*29*).

### Age-dependent, sex-stratified effect of sustained high BMI on incident disease

**Fig. 5.**
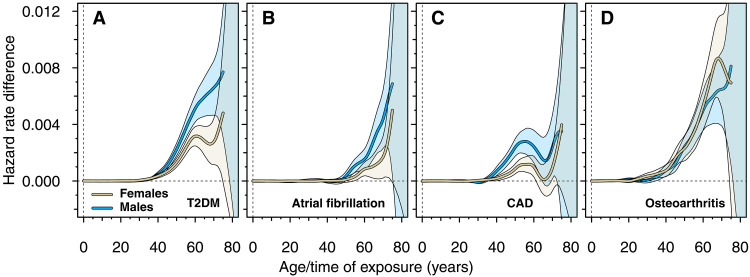
Trends of time-resolved, cumulative effects of sustained high BMI on disease in females and males. Time-series trends of the cumulative effects (fig. S12), estimated using Hodrick-Prescott filtering with λ=50 , are presented for (**A**) T2DM, (**B**) AF, (**C**) CAD, and (**D**) OA. Results for females are denoted in beige, while results for males are denoted in blue. Parametric bootstrap with 10,000 bootstrap samples was used to calculate the 95% CIs of the trends. While the effect on T2DM in males shows a monotonically increasing trend with increasing age, the initially increasing trend in females is abruptly halted at about 60 years of age, followed by an apparent decrease over the following 10 years. The plateau found for T2DM in the sex-combined analysis is essentially due to this temporary decline of the trend in females. In contrast, the trough in the trend of the effect on CAD is present in both sexes.

In general, trends were indicative of higher effects of BMI in males as compared to females, except for OA where trends were similar in both sexes up to roughly 60 years of age (Fig. 5, A to D). After about 67 years of age, there was a tendency for the OA trend to decrease in females but not in males. However, these results are uncertain with diverging CIs. The trough in the trend for CAD was present in both sexes (Fig. 5C). In contrast, distinctive trends were found for T2DM, where a trough was observed in females but not in males (Fig. 5A).

We explored the likelihood that the female-specific trough for T2DM could be coupled to menopause or to menopausal hormone therapy. First, we split postmenopausal females in two nonoverlapping samples, with regard to self-reported age at menopause. The mean age when entering menopause was 44.6 years in the “early menopause” sample and 53.6 years in the “late menopause” sample. This yielded a difference in means of 9 years. Despite this difference, however, no difference in the timing of the trough was observed between the two samples (fig. S13), which suggests that the observed trough is not directly related to the timing of menopause. Second, exogenous estrogen administered via menopausal hormone therapy, mainly prescribed to women in their 50s with menopausal symptoms, is recognized as having a beneficial effect on incident diabetes risk (*30*). To test whether this treatment could cause the female-specific trough for T2DM, we estimated the life-course effect of sustained high BMI on incident T2DM in nonusers of menopausal hormone therapy in the UKB. The analyses hinted that, also in nonusers, a similar trough was present (fig. S14). Menopausal hormone therapy may therefore not be the principal driver behind the observed temporary decline in the female-specific effect (Fig. 5A).

### Age-dependent, pathway-specific effect of sustained high BMI on incident disease

To explore potential heterogeneity in the causal pathways linking BMI to disease, we performed SNP-level clustering analyses (see Materials and Methods). These analyses could potentially shed further light on the underlying biological mechanisms behind the time-dependent patterns observed, particularly for the two cardiometabolic diseases T2DM and CAD. Distinct genetic clusters corresponding to different pathways, showed divergent temporal risk profiles for these diseases, including both risk-enhancing and protective effects (fig. S15). We found tentative evidence that the sex difference that was recognized in the time-dependent effect of BMI on T2DM (Fig. 5A) is primarily linked to SNPs affiliated with the high-risk pathway (see the Supplementary Materials). The trough observed in the trend for CAD (Fig. 4K) also appears to be mainly coupled to the high-risk pathway. These findings highlight the complexity of time-dependent genetic mechanisms and warrant further investigation.

### Impact of selection into UKB

The probability of participation into volunteer-based studies such as the UKB is seldom equal for all individuals (*31*). This may lead to nonrepresentative sampling of the target population and can potentially induce bias in effect estimates. For example, older individuals with high BMI are probably less likely to participate, as compared to younger individuals with a normal range in BMI. We examined whether such a selection could result in smaller effect estimates of the genetic instrument on BMI observed in the older sample (Fig. 3D) and assessed what impact such selection would have on our results.

The mean BMI-PGS for all individuals showed a decreasing trend with increasing age, similar to $\beta_{G}\left(t\right)$ in Fig. 3D (fig. S16). Here, we hypothesize that this decreasing trend is a result of a nonequal probability of selection into UKB, presuming that this selection depends on BMI and age. To test whether this selection influences our time-resolved MR results, we first estimated the corresponding expected decrease in mean BMI-PGS per year in the age range of 40 to 70 years (fig. S16), when UKB participants first visited the assessment centers. Second, we randomly appended “new” participants with high BMI to the sample, such that the originally decreasing trend in mean BMI-PGS was rectified and the age dependence was no longer detected (see Materials and Methods). Note that this is a relative correction in the sense that we only rectify the slope of the mean BMI-PGS in the age range of 40 to 70 years. Any offset of the mean BMI-PGS due to selection into UKB is not corrected for here.

The correction step resulted in an expanded UKB sample, with about 2.2% duplicated participants overall, while as many as 5% were duplicated in the oldest age strata (fig. S16). We adopted this expanded dataset to reestimate the effect of the PGS on BMI, including a linear interaction with age. Noticeably, the time dependence was substantially reduced (fig. S16), and the interaction coefficient was no longer significant ( P>0.05 for $\beta_{\text{linear}}$ ). If this correction procedure to some extent accounts for selection into UKB, then we can perform time-resolved MR using the expanded UKB sample and attain results that are supposedly less affected by bias due to participation. The findings of this analysis are presented in fig. S17. While there are minor systematic differences between the original life-course effects and the effects corrected for selection, the results are similar. In particular, all previously identified features and troughs were unaffected by the selection.

## DISCUSSION

In this study, we have estimated the age-varying effect of obesity on disease risk at unprecedented temporal resolution across the entire lifespan. We were thereby able to elucidate the dynamic etiology of obesity-related disease and identify several age-dependent characteristics that were unique to each disease investigated. We observed a clear trough in the life-course effect on CAD, in both sexes. This trough is likely due to preventive treatment of individuals with high BMI, as obesity is a major risk factor for CAD. Incidence rates of CAD have decreased in recent years, not only due to smoking cessation and reduced exposure to tobacco but also due to more frequent use of medications for lipid control, such as statins (*32*). Statins reduce low-density lipoprotein and triglyceride levels and have been shown to decrease the risk of cardiovascular disease. This makes them the first-choice agent for individuals at an elevated cardiovascular risk (*33*), such as individuals with high BMI. In support of this claim, the use of lipid-lowering treatment was significantly more frequent among overweight individuals in the UKB.

Since high blood pressure is known to increase the risk for AF (*27*, *28*), it is reasonable to expect that antihypertensive treatment is effective at mitigating the impact of SBP on AF. Consistent with this interpretation, we found a trough in the life-course effect of SBP on AF similar to what we observed for BMI on CAD, while no obvious troughs were visible, neither in the effect of BMI on AF nor in the effect of SBP on CAD. These latter results are broadly consistent with large meta-analyses of randomized control trials, which suggest that statin treatment does not have a substantial effect on AF risk (*29*), while blood pressure–lowering treatment reduces the risk of major cardiovascular events and coronary heart disease only in individuals with a high baseline SBP of ≥140 mmHg (*34*). Last, we note that although SBP is found to be only a moderate risk factor for AF at midlife, and despite the temporary decline in effect around 55 to 65 years of age, SBP quickly becomes a very strong risk factor at old age, with a cumulative effect that increases exceptionally fast with age.

Together, this indicates that features similar to the observed trough for CAD may be fingerprints of preventive interventions that can be detected in the population, which further validates the accuracy of our time-resolved MR method. With the high temporal resolution achieved, we have gained the possibility to survey effects of earlier implementations of population-wide interventions, in terms of whether these interventions were successful. Another aspect of large-scale intervention campaigns that becomes visually apparent when resolving effects in time is their impact on the causal inference and conclusions drawn from cohort studies. Partial mediation by preventive interventions that are introduced at a particular age span could considerably modify the total effect of an exposure on incident disease outcomes.

We also observed a female-specific trough in the cumulative effect of sustained high BMI on T2DM at an age span succeeding the age when women typically enter menopause and may use exogenous estrogen administered via menopausal hormone therapy. Since these agents have been shown to lower the risk of diabetes (*30*), menopausal hormone therapy could potentially explain the female-specific trough. However, this was not supported by our analyses, and the timing of the female-specific trough appeared not to be related to the timing of menopause at all. Another potential explanation could be that women are more inclined to visit a physician and more receptive to advice regarding primary prevention, such as weight reduction and healthier dietary recommendations. Greater adherence among women compared to men could therefore partially explain the observed differences between sexes (*35*). In general, the existence of troughs is compelling, and further investigations are warranted to elucidate their origins.

In MR, heterogeneity in excess of the uncertainty due to statistical error in individual SNP estimates is often interpreted as a consequence of horizontal pleiotropy that leads to the presence of invalid instruments (*36*). However, heterogeneity may also be directly coupled to disease etiology and could thereby shed light on the underlying biological mechanisms behind the time-dependent patterns observed in males and females for these diseases. Studies (*37*–*40*) have suggested that heterogeneous genetic effects could arise in situations where the effect of different variants is mediated via the exposure through different molecular mechanisms or that it is mediated via distinct components of a composite exposure, such as BMI. For example, it has been shown that while most of the BMI-increasing alleles also increase the risk of T2DM, a subset is instead associated with a protective effect. The two sets of variants are differentially associated with several mediating factors including fat distribution and lipid traits (*41*). These mediating factors could have different impact at different times in life. In agreement with these findings, we identified three clusters of BMI-associated SNPs, not only for T2DM but also for CAD. The clustering analyses indicate that the mechanisms underlying the troughs in the life-course effects on CAD and T2DM are potentially coupled to distinct subgroups of genetic variants, in particular SNPs that were associated with the highest risk of disease.

Our time-resolved MR approach complements other recent developments, such as estimation of time-varying effects with multivariable MR (*17*) and the multi–principal component (PC) MR method (*42*). In the time-dependent setting, multivariable MR is used to estimate the direct effect of being exposed to a risk factor, e.g., in childhood or adolescence on the risk of developing disease later in life. In contrast, time-resolved MR estimates the total effect of continuously being exposed to a risk factor over the entire lifespan up to a given age. In this respect, the two methods are designed to answer different causal questions, and the results presented here do not contradict the hypothesis that events early in life can influence the risk of developing disease in adulthood (*43*). Time-resolved MR may be used to address research questions such as when does a certain risk factor have the highest impact on disease risk during the life course or when should proactive interventions or preventive treatments connected to a certain risk factor be applied for most efficient response at the population level, e.g., lowest number needed to treat to prevent one event.

Using discrete sampling with a limited number of time points, as in multivariable MR, may not be sufficient for time-dependent causal inference and could lead to biased estimates (*19*). In time-resolved MR, the resolution of the time-dependent effect is set by the temporal resolution in the health registers and the number of events in the dataset. To ensure enough power in each time point, we adopted a yearly binning over the entire lifespan. This resolution is considerably higher than what is achieved in multivariable MR where, typically, only two broadly defined time points are used over the life course. To overcome this specific shortcoming of the multivariable MR method, Tian *et al.* (*42*) recently proposed to model exposure trajectories in continuous time using functional PCs analysis. This model circumvents the issue of discrete sampling. It is flexible and allows for complex forms of time-dependent effects. However, similar to multivariable MR, the method increasingly suffers from weak instrument-related problems with an increasing number of PC terms included in the model. The functional complexity of the time dependence that the method can handle in practice is thus limited, with currently available datasets. We note that the multi-PC MR method is presently implemented for continuous outcomes only, while our time-resolved MR method is implemented for binary disease outcomes, making them complementary to each other.

The present study has some limitations. To reduce population stratification, we restricted the analysis to European ancestry. Results may therefore have limited generalizability to other populations and would require confirmation in large-scale studies on other ethnical groups. As for all epidemiological and MR studies, time-dependent analyses are susceptible to biases related to selection and participation into the study. When accounting for selection, we found small but systematic differences in the overall effect amplitudes. Disease-specific features and troughs were, however, unaffected by selection. The assumption in the present study that a prompt change in the exposure should lead to an instant and irreversible change in the latent outcome may be a potential limitation, e.g., in situations when a change in the exposure only leads to a future change in the outcome. However, if $\beta_{G}$ depends weakly on time and can be considered constant between the time of change in exposure and the delayed time of change in latent outcome, then the Wald ratio as presented here will represent a good approximation of the time-dependent causal effect. We show that with additional information about disease biology, this assumption can be relaxed. Another limitation is that we only use genetic instruments for BMI that are evaluated in adults, and extrapolation of the time dependence of the BMI-PGS instrument toward young and old ages may pose a potential problem. However, sensitivity analyses showed that even for large systematic offsets at young ages, the bias in the MR estimates was negligible for the adult-onset diseases investigated here. In general, it is important that $\beta_{G}\left(t\right)$ is accurately estimated at all ages at which the effect of the instrument on disease is nonnegligible. There has been an increasing interest in recent years to identify genetic variants affecting BMI and other traits in children and adolescents (*44*). In the near future, sample sizes in these studies will be large enough to generate reliable results that can be incorporated into our models also to study childhood and adolescent-onset diseases.

In conclusion, we have shown that time-resolved MR accurately identifies the cumulative effect of obesity on disease risk at different ages across the lifespan. Time-resolved MR enables the identification of critical periods of heightened susceptibility, where an exposure such as sustained high BMI has the greatest impact on disease risk, something that standard MR approaches fail to capture. The time-resolved MR method can easily be adapted and applicable to any exposure for which valid genetic instruments can be identified and to any disease outcome for which time-to-event data are available through large biobank cohorts. Quantification of time-dependent effects opens a previously unexplored window for the study of underlying disease biology. We now have the potential to markedly increase our understanding of when disease-associated risk factors are of concern and when individuals would benefit the most from preventive treatment. This empowers us to tailor targeted and adaptable health campaigns, thereby addressing people’s changing health needs over time.

## MATERIALS AND METHODS

### Study population, exposure, outcomes, and covariates

The study was performed in the UKB, which is a large biomedical cohort with detailed information about genetics and health, collected both retrospectively and prospectively for more than half a million participants from across the United Kingdom (*45*). Participants, originally between 37 and 73 years of age, were initially invited for assessment in 2006–2010. They underwent physical examinations at which anthropometric traits, including BMI, were measured and were interviewed about lifestyle, health, and disease history via touchscreen questionnaires and verbal interviews. To reduce population stratification in the MR analyses, we used a subsample of UKB, including only participants who were both identified as Caucasians by genetic PCs analysis (data-field 22006) and self-identified as white British (data-field 21000). As a consequence, the results presented in this study may have limited generalizability to other ethnic populations. Additional filtering was performed, and related individuals with an estimated genetic relationship of >0.044 were removed, as were individuals with poor call rate (<95%), high heterozygosity (data-field 22010), or sex errors (data-field 22001). A total of 361,906 participants remained for downstream analyses. To avoid introducing bias due to overfitting of the PGS instrument, this sample was split in two independent, equally sized subsamples A and B ( $N_{A}=N_{B}=180,953$).

In this study, we investigated how BMI, as a modifiable risk factor, affects the rate of disease onset. BMI was measured for each individual at initial assessment (Table 1 and table S2). Before being used as an exposure in the models, we standardized the BMI measurements for each sex and age stratum using *z*-transformation (fig. S18). To compensate for low numbers in the outermost age strata, the lowest age stratum included all participants aged 40 years or below, while the highest age stratum included all participants aged 70 years or above. This standardization of BMI was performed, partly to circumvent the need for nonlinear adjustment for age in the models, including the GWASs, and partly to remove differences in the variance of BMI across age. In doing so, the time-dependent effect could be compared on a common scale over the entire range in age.

Information about disease onset dates was extracted from a curated dataset of first occurrence of disease (category 1712). The dataset was acquired from primary care data, hospital inpatient data, cause-of-death register records, and self-reported medical conditions reported at baseline or at subsequent UKB assessment visits, with each disease being mapped to a three-character international classification of diseases, 10th revision (ICD-10) code. The ICD-10 codes for the four disease outcomes investigated here can be found in table S2. Dates of first occurrence of disease were collected up to May 2022 and include diagnoses made both before UKB recruitment and those recorded in health registers after recruitment. The end of follow-up was set to 31 December 2020 to reduce data incompleteness. Age, in unit of years, was used as primary timescale. Age at first occurrence of disease was defined as the year of first occurrence minus year of birth. Similarly, age at end of follow-up was defined as min(2020,year of death) minus year of birth. However, to avoid spurious boundary effects from the modeling and low-number statistics present in the oldest age bins, participants were only followed until a maximum age of 76 years. Covariates (table S2) that were used in the modeling included sex, age at assessment, genotyping array, assessment center, and the first 25 genetic PCs.

The UKB was granted a generic Research Tissue Bank approval from the National Research Ethics Service and UKB’s Research Ethics Committee in 2010. This covers the large majority of research using the resource without separate ethical approval, including the research described in this study. An application to use UKB data for this project has been approved by the UKB Access Subcommittee (no. 15152). Ethical approval to store and analyze UKB data in Sweden has also been granted by the Swedish Ethical Review Authority (no. 2020–04415).

### GWAS of BMI and identification of lead SNPs

Genome-wide genotyping using two custom-designed microarrays (UK BiLEVE and Axiom Genotyping) with 807,411 and 820,967 genotyped SNPs, respectively, was performed on all individuals in UKB. In addition, imputation of more than 90 million variants was performed using UK10K (*46*) and 1000 Genomes (*47*), phase 3 as reference panels. SNPs with a minor allele frequency of >0.01, high call rate (>95%), and high imputation quality (>0.9) were kept for analysis. SNPs deviating from Hardy-Weinberg equilibrium (*P* < 10^−12^) were removed. After filtering, 8,560,996 SNPs remained.

To identify genome-wide significant SNPs for the BMI-PGS instruments and determine their effect on standardized (*z*-transformed) BMI, we performed a GWAS in each of the two subsamples of UKB ( $N_{A}=N_{B}=180,953$ ) using PLINK (v2.00a2.3LM), adopting the multiple linear regression model

$$E\left[x_{\text{BMI},i}\right]=\beta_{0}+\beta_{g}\cdot g_{i,j}+\text{covariates}$$

where $g_{i,j}$ denotes the genotype of SNP j for individual i and $x_{\text{BMI},i}$ is the standardized BMI for individual i . The covariates included in the BMI GWASs were sex, age at assessment (standardized), genotyping array (UK BiLEVE or Axiom), assessment center, and the first 25 genetic PCs.

We used the clump function in PLINK (*48*) to identify independent lead SNPs for BMI. We defined two SNPs to be independent lead SNPs if they both were genome-wide significant for BMI with $P<5\times 10^{-8}$ and if they either were in low linkage disequilibrium ( $R^{2}<0.0001$ ) or if they were at least 20 Mbp apart. The clumping was performed in each GWAS separately.

We used the exact same procedure and the same covariates to extract lead SNPs associated with sex- and age-stratified standardized SBP. This was done to estimate cumulative effects of SBP on AF and on CAD in one of the sensitivity analyses. In total, 66 independent lead SNPs were identified in subsample A , while 61 independent lead SNPs were identified in subsample B . Subsequent Steiger filtering (see below) was performed to remove SNPs that did not explain significantly more variation in SBP than in disease [false discovery rate (FDR) > 0.05].

### Steiger filtering and the generation of PGSs for BMI

We performed Steiger filtering (*25*) to select valid genetic instruments for each exposure-outcome relation before the generation of the disease-specific BMI-PGS. A valid variant is assumed to explain more variance in the exposure (BMI) than in the disease outcome. In this way, the risk of bias due to inclusion of invalid SNPs that potentially have an effect in the reversed causal direction is reduced. To avoid introducing bias using the target samples for SNP selection (*24*), we identified and removed SNPs suspected to be primarily related to the outcome within each selection sample. Since the two correlations to be compared, i.e., $\rho_{g,x}$ and $\rho_{g,y}$ for exposure x and outcome y , respectively, were estimated within the same sample, we adopted a test for comparison of two dependent, overlapping correlation coefficients with one variable ( g ) in common (*49*). For this test, $\rho_{g,x}$ and $\rho_{g,y}$ were first transformed using Fisher’s *z*, such that $Z_{*}=\text{atanh}\left(\rho_{*}\right)$ . The test statistic is given by

$$z=\left(Z_{g,x}-Z_{g,y}\right)\cdot \sqrt{\frac{N-3}{2h\left(1-\rho_{x,y}\right)}}$$

where N is the sample size and h is

$$h=\frac{1-f\bar{\rho^{2}}}{1-\bar{\rho^{2}}}$$

with

$$f=\frac{1}{2}\cdot \frac{1-\rho_{x,y}}{1-\bar{\rho^{2}}}$$

and $\bar{\rho^{2}}=\left({\rho_{g,x}}^{2}+{\rho_{g,y}}^{2}\right)/2$ . The correlation $\rho_{x,y}$ was extracted from the supposed relation $\rho_{g,y}=\rho_{x,y}\rho_{g,x}$ , assuming that X⟶Y . Here, we simply estimated $\rho_{x,y}=\rho_{g,y}/\rho_{g,x}$ from the data using the median value of $\rho_{g,y}/\rho_{g,x}$ for all genome-wide SNPs $i=1,\ldots ,n_{j}$ in the selection sample j=1,2 . We adopted a one-tailed test, with the alternative hypothesis $H_{1}:\rho_{g,x}>\rho_{g,y}$ . Correction for multiple testing was applied using the Benjamini-Hochberg procedure (*50*), separately for each disease and within each selection sample. Variants with an FDR of >0.05 were removed from the disease-specific SNP set since these SNPs failed to explain significantly more variation in the exposure (BMI) than in the outcome (disease). Last, we note that a Steiger test performed in a single sample is generally conservative due to winner’s curse.

After the Steiger filtering step, disease-specific PGSs were generated using selected SNPs (data S1). First, two BMI-PGSs were generated independently for the individuals in each of the two subsamples A and B . Hence, a score for each individual, $i_{A}$ , in subsample A was calculated using the genetic variants identified in subsample B and selected for disease D in sample B (Steiger filtering), such that

$${\text{PGS}}_{i_{A},D}=B_{B_{D}}^{T}G_{i_{A},B_{D}}$$

where $B_{B_{D}}^{T}=\left(\beta_{g,1},\ldots ,\beta_{g,n_{B_{D}}}\right)$ denotes the row vector of weights of the $n_{B_{D}}$ selected SNPs, i.e., the SNPs’ effect on *z*-transformed BMI in subsample B, while $G_{i_{A},B_{D}}={\left(g_{i_{A},1},\ldots ,g_{i_{A},n_{B_{D}}}\right)}^{T}$ denotes the column vector of the corresponding $n_{B_{D}}$ genotypes for individual $i_{A}$ . In the same manner, a score, ${\text{PGS}}_{i_{B},D}$ , was calculated for each individual, $i_{B}$ , in subsample B using the $n_{A_{D}}$ genetic variants identified in subsample A and selected for disease D in sample A . These two PGSs were then standardized to zero mean and unit variance within each subsample, before the two subsamples were remerged into a single sample including all 361,906 participants. In the main analyses, a unique BMI-PGS was generated for each disease. In the hierarchical clustering analysis, a unique BMI-PGS was generated for each identified cluster (data S1). Last, for the secondary analyses with SBP as exposure, a unique SBP-PGS was generated for AF and CAD, respectively.

### Time-dependent modeling

The following derivation assumes that a prompt change in exposure results in an instant and irreversible change in the latent outcome. A relaxation of this assumption is briefly discussed in the Supplementary Materials. Let $\Upsilon_{i}=\Upsilon_{i}\left(t\right)$ describe the hazard rate for individual i as a continuous function of time t , and let $\upsilon_{i}\left(t\right)=d\Upsilon_{i}/\mathrm{dt}={\dot{\Upsilon }}_{i}\left(t\right)$ denote the change in the hazard rate per unit time. Now, suppose that the latent outcome $\upsilon_{i}\left(t\right)$ can be described by the linear model

$$\upsilon_{i}\left(t\right)=\gamma_{0}\left(t\right)+\gamma \left(t\right)\cdot x_{i}\left(t\right)+u^{\upsilon }\left(u_{i}\left(t\right),t\right)+z^{\upsilon }\left(z_{i}\left(t\right),t\right)+\epsilon_{i}^{\upsilon }\left(t\right)$$ (1)

where $x_{i}$ denotes the time-dependent exposure, $u^{\upsilon }$ and $z^{\upsilon }$ denote some time-dependent functions of a time-dependent confounding variable u and a covariate z , respectively, and where $\epsilon_{i}^{\upsilon }$ is a random error. The time-dependent effect of x on the latent outcome is denoted by γ(t) , while $\gamma_{0}$ denotes the intercept. To ensure that the hazard rate $\Upsilon_{i}$ is nonnegative, let Eq. 1 be constrained, such that $\int_{0}^{T}\upsilon_{i}\left(t\right)\mathrm{dt}+C_{0}\geq 0$ for all T and some arbitrary constant $C_{0}\geq 0$ . Note that the time-dependent variables u and z , and their corresponding time-dependent effects on outcome do not have to be separable in general.

Furthermore, let the exposure x be described by

$$x_{i}\left(t\right)=\beta_{0}\left(t\right)+\beta_{G}\left(t\right)\cdot G_{i}+u^{x}\left(u_{i}\left(t\right),t\right)+z^{x}\left(z_{i}\left(t\right),t\right)+\epsilon_{i}^{x}\left(t\right)$$ (2)

where $G_{i}$ denotes the genetic instrument, while $u^{x}$ and $z^{x}$ are time-dependent functions of u and z that both influence x , and $\epsilon_{i}^{x}$ is the error. It is assumed that x is linear in G , where the time-dependent effect of the genetic instrument on exposure is given by $\beta_{G}\left(t\right)$.

In this context, a genetic instrument used in MR may be regarded as having a lifetime effect on outcome, via the exposure or risk factor, up to time t=T . Such a risk factor would accumulate the risk of an outcome to occur as we get older. What we observe is then a realization of the integral version of Eq. 1 for the hazard rate $\Upsilon_{i}\left(T\right)$ that is built-up over time, such that

$$\Upsilon_{i}\left(T\right)\equiv \int_{0}^{T}\upsilon_{i}\left(t\right)\mathrm{dt}+C_{0}=\int_{0}^{T}\gamma \left(t\right)\cdot x_{i}\left(t\right)\mathrm{dt}+$$

$$+\int_{0}^{T}\gamma_{0}\left(t\right)\mathrm{dt}+\int_{0}^{T}u^{\upsilon }\left(u_{i}\left(t\right),t\right)\mathrm{dt}+\int_{0}^{T}z^{\upsilon }\left(z_{i}\left(t\right),t\right)\mathrm{dt}+C_{0}+\epsilon_{i}^{\Upsilon }\left(T\right)$$ (3)

If we insert the expression for the exposure from Eq. 2 into Eq. 3 and simplify, then we obtain

$$\Upsilon_{i}\left(T\right)=\left(\int_{0}^{T}\gamma \left(t\right)\cdot \beta_{G}\left(t\right)\mathrm{dt}\right)\cdot G_{i}+A\left(T\right)+U_{i}\left(T\right)+Z_{i}\left(T\right)+\epsilon_{i}\left(T\right)$$ (4)

where A corresponds to the intercept, while $U_{i}$ and $Z_{i}$ are functions of the confounder and the covariate, respectively, with $\epsilon_{i}$ denoting the random error. Here, the integral

$$Η\left(T\right)\equiv \int_{0}^{T}\gamma \left(t\right)\cdot \beta_{G}\left(t\right)\mathrm{dt}$$ (5)

is identified to represent the accumulated effect, from conception up to time T , of the genetic instrument on disease, where the effect of the instrument on exposure at time t≤T is given by $\beta_{G}\left(t\right)$ . Hence, Η denotes the observed effect of the instrument on outcome.

Now, suppose that $x_{i}\left(t\right)$ and $x_{j}\left(t\right)$ denote the exposure at time t of individual i and j , respectively, and let individual j have a unit higher exposure than individual i at all times, such that $x_{j}\left(t\right)=x_{i}\left(t\right)+1,\forall t$ . From Eq. 3, keeping all other variables fixed, we then have that

$$\begin{array}{c} \Upsilon_{j}\left(T\right)-\Upsilon_{i}\left(T\right)\\ =\int_{0}^{T}\gamma \left(t\right)\cdot \left(x_{i}\left(t\right)+1\right)\mathrm{dt}-\int_{0}^{T}\gamma \left(t\right)\cdot x_{i}\left(t\right)\mathrm{dt}=\int_{0}^{T}\gamma \left(t\right)\mathrm{dt}=\Gamma \left(T\right) \end{array}$$ (6)

which is the sought-after cumulative effect of the exposure on outcome, i.e., the difference in the hazard rate at time T due to a unit higher value in exposure throughout the entire time from t=0 up to time t=T.

From Eq. 1, we identify γ(t) as the latent, momentaneous effect of the exposure exerted during the time interval t to t+dt . In terms of the hazard rate, γ describes the change in the hazard, being exposed to a unit higher value in exposure for one additional unit of time, at time t . Evidently, Γ cannot be recovered by simply taking the ratio $Η\left(T\right)/\beta_{G}\left(T\right)$ with Η(T) defined in Eq. 5, unless $\beta_{G}$ is independent on time (*11*). However, by first taking the derivative of Η with respect to time, such that

$$\dot{Η}\left(t\right)\equiv \frac{\mathrm{dΗ}}{\mathrm{dt}}=\gamma \left(t\right)\cdot \beta_{G}\left(t\right)$$ (7)

then divide by $\beta_{G}\left(t\right)$ , and integrate, i.e.,

$$\int_{0}^{T}\frac{\mathrm{dΗ}/\mathrm{dt}}{\beta_{G}\left(t\right)}dt=\int_{0}^{T}\frac{\gamma \left(t\right)\cdot \beta_{G}\left(t\right)}{\beta_{G}\left(t\right)}\mathrm{dt}=\int_{0}^{T}\gamma \left(t\right)\mathrm{dt}=\Gamma \left(T\right)$$ (8)

the cumulative effect Γ(T) may formally be recovered. Here, the ratio

$$\frac{\dot{Η}\left(t\right)}{\beta_{G}\left(t\right)}=\frac{\mathrm{dΗ}/\mathrm{dt}}{\beta_{G}\left(t\right)}=\gamma \left(t\right)$$ (9)

denotes the appropriate Wald ratio. We will estimate the time derivative $\dot{Η}\left(t\right)$ using the finite difference method. In doing so, we assume that the effect of the genetic instrument on exposure does not change within the temporal resolution, i.e., within each age bin. This should be an acceptable assumption, given the high temporal resolution of 1 year and the relatively moderate change in effect across the full lifespan (Fig. 3D).

### Estimation of the instrument effect on exposure $\beta_{G}\left(t\right)$

In the main analyses, we used a quartic model for the time dependence of the effect of the PGS instrument on exposure (*z*-transformed BMI). Specifically, we assumed that

$$E\left[x_{i}\left(t\right)\right]=\beta_{0}\left(t\right)+\beta_{G}\left(t\right)\cdot G_{i}+\text{covariates}$$

with $\beta_{0}\left(t\right)=\beta_{0}$ and

$$\beta_{G}\left(t\right)=a+\mathrm{bt}+ct^{4}$$ (10)

This form was chosen such that the function would follow the observed UKB data in the interval [40,70] years in a “quadratic fashion,” with a linear extrapolation toward low ages. Here, t denotes age at assessment, while the covariates include sex, genotyping array, assessment center, and the first 25 genetic PCs. To reduce potential biases, e.g., due to reversed causation, the effect of the BMI-PGS on *z*-transformed BMI was only estimated in individuals who were event free at assessment for the disease under investigation (*51*), when BMI was measured. In the sex-stratified analyses, we also estimated the time-dependent effect of the PGS on BMI in males and females, separately. The time-dependent effect estimates $\hat{\beta_{G}}\left(t\right)$ may therefore differ slightly between diseases and between sexes. Note that although effect estimates $\hat{\beta_{G}}\left(t\right)$ were uncertain at t<40 years of age (see, e.g., fig. S6), life-course effects were found to be insensitive to this uncertainty for the adult-onset diseases investigated here (fig. S19).

For comparison, we also adopted a linear interaction model with age, such that

$$\beta_{G}\left(t\right)=a+\mathrm{bt}$$ (11)

The interaction coefficient b in the linear model (*11*) was mainly used to classify the individual lead genetic variants ( $g_{i,j}$ ) by the strength of their time dependence (Fig. 3). When discussed in the main text (see the “Age-dependent effect of PGS on BMI” section), this coefficient is referred to as $\beta_{\text{linear}}$ , while $\beta_{\text{main effect}}$ (Fig. 3) refers to the estimate of the time-fixed effect, taken from the standard GWAS model. Similar to the GWAS, the estimation of $\beta_{\text{linear}}$ was performed within each subsample, without any filtering on disease, and including sex, standardized age at assessment, genotyping array, assessment center, and the first 25 genetic PCs as covariates.

Note that for the PGS generated from the set of SNPs that showed a protective effect on T2DM (fig. S15), the quartic model Eq. 10 resulted in a negative effect when extrapolating toward young ages. This led to a violation of the first MR assumption since the instrument was not strongly associated with BMI at all ages $\left[\mathrm{\beta G}t=0\right.$ at some t ]. For this case, we instead used the linear model in Eq. 11 to estimate the time dependence of $\beta_{G}\left(t\right)$.

### Derivation of the 95% CI for $\hat{\beta_{G}}\left(t\right)$

Assuming that the time t is a fixed parameter, the variance of the estimator for $\beta_{G}\left(t\right)$ in Eq. 10 is given by

$$\begin{array}{c} \text{Var}\left[\hat{\beta_{G}}\left(t\right)\right]=\text{Var}\left[\hat{a}+\hat{b}t+\hat{c}t^{4}\right]=\text{Cov}\left[\hat{a}+\hat{b}t+\hat{c}t^{4},\hat{a}+\hat{b}t+\hat{c}t^{4}\right]\\ =\text{Var}\left[\hat{a}\right]+t^{2}\cdot \text{Var}\left[\hat{b}\right]+t^{8}\cdot \text{Var}\left[\hat{c}\right]\\ +2t\cdot \text{Cov}\left[\hat{a},\hat{b}\right]+2t^{4}\cdot \text{Cov}\left[\hat{a},\hat{c}\right]+2t^{5}\cdot \text{Cov}\left[\hat{b},\hat{c}\right] \end{array}$$ (12)

The variance and covariance terms of the coefficients in Eq. 12 are estimated in the fit to the regression model (Eq. 10) and may be extracted from the vcov()function in R. It follows that the 95% CI, as a function of t , is given by

$$\hat{\beta_{G}}\left(t\right)\pm z_{1-\alpha /2}\sqrt{\hat{\text{Var}\left[\hat{\beta_{G}}\left(t\right)\right]}}$$

with $z_{1-\alpha /2}=1.96$ for normally distributed errors and significance level α=0.05 . The corresponding 95% CI for the linear model in Eq. 11 is derived in a similar manner.

### Estimation of the instrument effect on disease Η(t)

To estimate lifetime effects on an absolute scale from time-to-event data, we made use of the additive hazard model by Aalen (*22*, *52*), in which time dependence is naturally incorporated. In general, the hazard function is described by the linear model

$$\begin{array}{c} \lambda \left(t|x\right)=b{\left(t\right)}^{T}x=b_{0}\left(t\right)+b_{G}\left(t\right)\cdot G+b_{1}\left(t\right)\cdot x_{1}+\ldots \\ +b_{p}\left(t\right)\cdot x_{p}=b_{0}\left(t\right)+b_{G}\left(t\right)\cdot G+\sum_{m=1}^{p}b_{m}\left(t\right)\cdot x_{m} \end{array}$$ (13)

for the covariate vector $x={\left(1,G,x_{1},\ldots ,x_{p}\right)}^{T}$ , where $b_{0}\left(t\right)$ denotes the baseline hazard. In Aalen’s model, the cumulative regression coefficients $\hat{B_{m}}\left(t\right)$ are estimated rather than the regression coefficients $\hat{b_{m}}\left(t\right)$ . Any estimator $\hat{B_{m}}$ at time t is defined as

$$\hat{B_{m}}\left(t\right)=\sum_{t_{\psi }\leq t}\hat{b_{m}}\left(t_{\psi }\right)$$ (14)

Here, $t_{\psi }$ denotes the time at event ψ=1,…,n , where n is the total number of observed events in the data. Between event times, the estimator $\hat{B_{m}}$ does not change. Using Aalen’s model in Eq. 13 to model the (accumulated) hazard rate Υ(t) described in Eq. 4, we identify the estimator for the observed effect of the instrument on disease in Eq. 5 as the regression coefficient $\hat{b_{G}}\left(t\right)=\hat{Η}\left(t\right)$ to the instrument G , with the corresponding cumulative regression coefficient estimator $\hat{B_{G}}\left(t\right)$.

Since the unit of time is measured in years, with the number of events per year possibly being much larger than 1, random noise is used to break ties within each year in the aalen()function of the timereg R package. We thus defined the regression coefficient $\hat{Η}\left(t_{k}\right)$ at $t=t_{k}$ as

$$\hat{Η}\left(t_{k}\right)\Delta t=\hat{Η}\left(t_{k}\right)=\hat{B_{G}}\left(t_{\psi }\right)-\hat{B_{G}}\left(t_{\chi }\right)$$ (15)

where $t_{\psi }$ denotes the event with the largest random noise added to the year $t_{k}$ , i.e., at age k , and where $t_{\chi }$ denotes the event with the largest random noise added to the nearest preceding year $t_{k-1}$ , at age k−1 . The unit of time is Δt=1 year. Note that the number of events occurring at age k is given by ψ−χ . Estimates of the two terms $\hat{B_{G}}\left(t_{\psi }\right)$ and $\hat{B_{G}}\left(t_{\chi }\right)$ can be extracted from the output of aalen().

We note that Aalen’s model has several desirable properties. The hazard rate difference of Aalen’s model can be a more appropriate quantity for causal inference, e.g., as compared to the hazard ratio of the Cox model, since it is less susceptible to bias connected with the breaking of randomization in survival data (*53*). Furthermore, the effect estimator of Aalen’s model is collapsible in continuous time (*54*). In practice, it is also collapsible in discretized time when the event rate in each discrete time interval may be considered small (*55*), i.e., when $e^{-x_{k}}\approx 1-x_{k}$ for the number of events $x_{k}$ in time interval k . In the present study, the maximal observed number of events in one unit time interval (year) across all diseases was about 0.01≪1 . Making use of an additive model also reduces the phenomenon of “effect dilution,” which may drive relative effects such as the hazard ratio toward null at old age. This can make the interpretation of effects less straightforward. For example, a hazard ratio that shows a decreasing trend with increasing age may do so because of a background rate that increases with increasing age, rather than having a hazard of exposed individuals that decreases with age relative to a time-fixed background.

In the modeling of the instrument effect on disease, the same covariates were included as in the modeling of the instrument effect on BMI, i.e., sex, genotyping array, assessment center, and the first 25 genetic PCs. Similar to the main exposure, all covariates were modeled as having a time-dependent effect on disease outcome, i.e., no variable was set to constant.

### Estimation of the derivative of the instrument effect on disease $\dot{Η}\left(t\right)$

To approximate the time derivative of Η(t) in Eq. 5 from the estimated lifetime effect of the instrument on disease, we make use of the central finite difference method, such that the derivative at the midpoint $t=t_{k-1/2}$ is given by

dΗdt^t=tk−1/2=Η^(tk)−Η^(tk−1)tk−tk−1+𝒪(tk−tk−1)≈Η^(tk)−Η^(tk−1)tk−tk−1=Η^(tk)−Η^(tk−1) (16)

since $\Delta t=t_{k}-t_{k-1}=1$ year, as above. Here, $\hat{Η}\left(t_{k}\right)$ denotes the estimate of the cumulative effect of the instrument on outcome, as given in Eq. 15. For adult-onset diseases, we may typically assume that Η(0)=Γ(0)=0.

### Estimation of the lifetime effect of exposure on disease

From the expressions in Eqs. 9 and 16, the central finite-difference estimate of the momentaneous effect at the midpoint $t=t_{k-1/2}$ is given by

$$\hat{\gamma }\left(t_{k-1/2}\right)=\frac{\hat{\mathrm{dΗ}}}{\mathrm{dt}}/\beta_{G}\left(t_{k-1/2}\right)\approx \frac{\hat{Η}\left(t_{k}\right)-\hat{Η}\left(t_{k-1}\right)}{\beta_{G}\left(t_{k-1/2}\right)}$$ (17)

where we assume that $\hat{Η}\left(t_{0}=0\right)=0$ . Now, to obtain an estimate of Γ in Eq. 8, we must numerically integrate Eq. 17 over time. This can be done, e.g., by adopting the midpoint rule with Δt=1 . An estimate for the lifetime causal effect of exposure on disease up to time $t=t_{k}$ is then given by

$$\hat{\Gamma }\left(t_{k}\right)=\hat{\int_{0}^{t_{k}}\frac{\mathrm{dΗ}/\mathrm{dt}}{\beta_{G}\left(t\right)}\mathrm{dt}}\approx \sum_{i=1}^{k}\hat{\gamma }\left(t_{i-1/2}\right)$$ (18)

where $\hat{\gamma }\left(t_{i-1/2}\right),i=1,\ldots ,k$ , are defined in Eq. 17 and $\hat{\gamma }\left(t_{0}=0\right)=0$.

For any downstream analysis in which the causal effect estimation of Γ was used as input, e.g., for calculating trends of the cumulative and momentaneous effects, we deployed the midpoint rule in Eq. 18 as an approximation of the integral in Eq. 8. We chose the midpoint rule to minimize the correlation between time points. However, for the visual presentation of $\hat{\Gamma }$ in the main text (see Fig. 4, E to H and the “Age-dependent, sex-combined effect of sustained high BMI on incident disease” section), we instead used the trapezoidal rule as an approximation of the integral in Eq. 8. By this choice, there is a gain in power that resulted in smaller standard errors. The causal effect estimates in adjacent time points are, however, no longer uncorrelated.

Hence, for an interior point $t_{k-1/2},k\geq 2$ , we have that

$$\begin{array}{c} \hat{\Gamma }\left(t_{k-1/2}\right)=\hat{\int_{0}^{t_{k-1/2}}\frac{\mathrm{dΗ}/\mathrm{dt}}{\beta_{G}\left(t\right)}\mathrm{dt}}\approx \frac{\Delta t\cdot \hat{\gamma }\left(t_{1/2}\right)}{4}+\frac{\Delta t\cdot \hat{\gamma }\left(t_{1/2}\right)}{2}\\ +\Delta t\cdot \sum_{i=2}^{k-1}\hat{\gamma }\left(t_{i-1/2}\right)+\frac{\Delta t\cdot \hat{\gamma }\left(t_{k-1/2}\right)}{2}=\frac{1}{4}\hat{\gamma }\left(t_{1/2}\right)\\ +\frac{1}{2}\hat{\gamma }\left(t_{1/2}\right)+\sum_{i=2}^{k-1}\hat{\gamma }\left(t_{i-1/2}\right)+\frac{1}{2}\hat{\gamma }\left(t_{k-1/2}\right) \end{array}$$ (19)

where we assume that Δt=1 in the last equality. The first term denotes the approximation of the integral in the interval $\left[0,t_{1/2}\right]=\left[0,1/2\right]$ where we assume that $\hat{\gamma }\left(t_{0}=0\right)=0$ , while the three last terms approximate the integral between $\left[t_{1/2},t_{k-1/2}\right]$ using the trapezoidal rule.

### Estimation of the variance of the instrument’s effect on outcome

Since the increments $\hat{b_{G}}\left(t_{\psi }\right)$ from Aalen’s model are uncorrelated, the variance may be written as

$$\begin{array}{c} \text{Var}\left[\hat{B_{G}}\left(t\right)\right]=\text{Var}\;\left[\sum_{t_{\psi }\leq t}\hat{b_{G}}\left(t_{\psi }\right)\right]=\sum_{t_{\psi }\leq t}\text{Var}\left[\hat{b_{G}}\left(t_{\psi }\right)\right] \end{array}$$ (20)

Now, given the definition for the instrumental effect estimate $\hat{Η}\left(t_{k}\right)$ at age $t_{k}$ in Eq. 15, the variance of $\hat{Η}\left(t_{k}\right)$ may be expressed as, using Eqs. 14 and 20

$$\begin{array}{c} \text{Var}\left[\hat{Η}\left(t_{k}\right)\right]=\text{Var}\left[\hat{B_{G}}\left(t_{\psi }\right)-\hat{B_{G}}\left(t_{\chi }\right)\right]\\ =\text{Var}\left[\sum_{j=1}^{\psi }\hat{b_{G}}\left(t_{j}\right)-\sum_{j=1}^{\chi }\hat{b_{G}}\left(t_{j}\right)\right]=\text{Var}\left[\sum_{j=\chi }^{\psi }\hat{b_{G}}\left(t_{j}\right)\right]\\ =\sum_{j=\chi }^{\psi }\text{Var}\left[\hat{b_{G}}\left(t_{j}\right)\right]=\sum_{j=1}^{\psi }\text{Var}\left[\hat{b_{G}}\left(t_{j}\right)\right]-\sum_{j=1}^{\chi }\text{Var}\left[\hat{b_{G}}\left(t_{j}\right)\right]\\ =\text{Var}\left[\hat{B_{G}}\left(t_{\psi }\right)\right]-\text{Var}\left[\hat{B_{G}}\left(t_{\chi }\right)\right]=\sigma_{k}^{2} \end{array}$$ (21)

where estimates of the two terms $\text{Var}\left[\hat{B}\left(t_{\psi }\right)\right]$ and $\text{Var}\left[\hat{B}\left(t_{\chi }\right)\right],\chi <\psi$ , can be extracted from the output of aalen().

From the expression in Eq. 29, we note that the variance of $\hat{\gamma }$ at time $t_{k-1/2}$ , given in Eq. 17, can to first order be estimated by

Var[γ^(tk−1/2)]=Var[dΗdt^t=tk−1/2βG(tk−1/2)]≈Var[Η^(tk)−Η^(tk−1)βG(tk−1/2)]≈1βG2(tk−1/2)(σk2+σk−12) (22)

### Variance estimation and 95% CI of $\hat{\Gamma }\left(t_{k}\right)$ for the midpoint rule

From the expression of the central finite-difference estimate of the momentaneous effect in Eq. 17 and the Riemann sum in Eq. 18, it follows that the variance of the lifetime effect $\hat{\Gamma }$ , accumulated up to time $t_{k}$ , may be estimated by

$$\text{Var}\left[\hat{\Gamma }\left(t_{k}\right)\right]\approx \frac{\sigma_{k}^{2}}{\beta_{G}^{2}\left(t_{k}\right)}$$ (23)

A derivation of the expression in Eq. 23 is given in the Supplementary Materials.

Then, from the result in Eq. 23, the 100(1−α) % pointwise CI of $\hat{\Gamma }\left(t_{k}\right)$ estimated from Eq. 18 can be expressed as

$$\hat{\Gamma }\left(t_{k}\right)\pm z_{1-\alpha /2}\cdot \frac{\sigma_{k}}{\left|\beta_{G}\left(t_{k}\right)\right|}$$ (24)

where $z_{1-\alpha /2}$ denotes the upper 1−α/2 percentile of the standard normal distribution and $\sigma_{k}=\sqrt{\sigma_{k}^{2}}$ is the SE of the instrument’s effect on disease at time $t_{k}$ , with variance given in Eq. 21.

### Variance estimation of $\hat{\Gamma }\left(t_{k-1/2}\right)$ for the trapezoidal rule

An approximation of the variance of the estimator $\hat{\Gamma }$ in Eq. 19, derived from the trapezoidal rule, is given by (see Supplementary Materials for a derivation)

$$\text{Var}\left[\hat{\Gamma }\left(t_{k-1/2}\right)\right]\approx \frac{\sigma_{1}^{2}}{16\cdot \beta_{G}^{2}\left(t_{1}\right)}+\frac{\sigma_{k-1}^{2}}{4\cdot \beta_{G}^{2}\left(t_{k-1}\right)}+\frac{\sigma_{k}^{2}}{4\cdot \beta_{G}^{2}\left(t_{k}\right)}$$ (25)

for an interior point $t=t_{k-1/2},k\geq 3$ . There are two special cases for the variance at the two initial time points $t_{1/2}$ and $t_{3/2}$ , i.e., when k=1 and k=2 . For k=1 , $\text{Var}\left[\hat{\Gamma }\left(t_{1/2}\right)\right]\approx \sigma_{1}^{2}/16\beta_{G}^{2}\left(t_{1}\right)$ , and for k=2 , $\text{Var}\left[\hat{\Gamma }\left(t_{3/2}\right)\right]\approx \sigma_{1}^{2}/16\beta_{G}^{2}\left(t_{1}\right)+\sigma_{2}^{2}/4\beta_{G}^{2}\left(t_{2}\right)$.

In this study, we use the expression in Eq. 25, together with the special cases for k=1 and k=2 , to estimate the variance of $\hat{\Gamma }$ when estimated using the trapezoidal rule. However, we note that at large times, i.e., for k≫1 , it can often be assumed that $\sigma_{1}^{2}\ll \sigma_{k-1}^{2}\approx \sigma_{k}^{2}$ . If so, then the variance in Eq. 25 reduces to $\sigma_{k}^{2}/2\beta_{G}^{2}\left(t_{k}\right)$ , which is half the variance of that in Eq. 23. At early times, i.e., for k≈3 , we have instead that $\text{Var}\left[\hat{\Gamma }\left(t_{k-1/2}\right)\right]≲9\sigma_{k}^{2}/16\beta_{G}^{2}\left(t_{k}\right)$ , which approximately amounts to about $\sigma_{k}^{2}/2\beta_{G}^{2}\left(t_{k}\right)$ as well. Hence, an approximate but simpler expression for the variance of $\hat{\Gamma }\left(t_{k-1/2}\right)$ than that in Eq. 25 may be given by

$$\text{Var}\left[\hat{\Gamma }\left(t_{k-1/2}\right)\right]\approx \frac{\sigma_{k}^{2}}{2\cdot \beta_{G}^{2}\left(t_{k}\right)},\forall k\in \left[1,\ldots ,n\right]$$ (26)

where k=n corresponds to the maximum age, at which the calculations are terminated.

### Nonparametric estimation of the trend of γ

One of the tools that can generally be used to investigate how the cumulative effect Γ changes over time is to study its derivative $\dot{\Gamma }$ . Moreover, if it can be assumed that the response in the hazard rate to a change in exposure is instant and irreversible, i.e., disease latencies are short, then $\dot{\Gamma }$ may be interpreted as the momentaneous effect γ=γ(t) and can be used to identify specific causal time periods for the exposure on outcome.

However, γ(T) at time t=T , defined in Eq. 9 and estimated from the expression in Eq. 17, cannot adequately be characterized because of the inherent uncertainty in the data—the estimated function will simply be too noisy. To overcome this problem, we instead estimated a smoothed version of the momentaneous effect, denoted by $\bar{\gamma }\left(T\right)$ , that describes the trend of γ . We defined $\bar{\gamma }\left(T\right)$ as the momentaneous effect averaged over a finite time interval around T . Nonparametric, i.e., local, estimation of $\bar{\gamma }$ was performed by first fitting a linear model to the estimated cumulative effects $\hat{\Gamma }$ in the local neighborhood of T using weighted linear regression. The derivative of the fitted model at t=T was then taken as an estimate of $\bar{\gamma }\left(T\right)$.

In matrix form, the model parameters $\hat{\xi \left(T\right)}$ at time T of a weighted linear regression are given by the expression

$$\left(X^{T}K\left(T\right)X\right)\hat{\xi \left(T\right)}=X^{T}K\left(T\right)y$$ (27)

where X is the design matrix, $y={\left({\hat{\Gamma }}_{1},\ldots ,{\hat{\Gamma }}_{n}\right)}^{T}$ denotes the vector of cumulative effect estimates ${\hat{\Gamma }}_{i}=\hat{\Gamma }\left(t_{i}\right),i=1,\ldots ,n$ , and the diagonal matrix K denotes the weight matrix. Formally, K=K(T) is given by

$$K\left(T\right)=\left(\begin{array}{ccc} K\left(t_{1}-T\right) & \; & 0\\ \; & \begin{array}{ccc} K\left(t_{1}-T+1\right) & \; & \;\\ \; & \begin{array}{cc} \ddots & \;\\ \; & K\left(t_{i}-T\right) \end{array} & \;\\ \; & \; & \ddots \end{array} & \;\\ 0 & \; & K\left(t_{n}-T\right) \end{array}\right)$$ (28)

for some weight kernel K . Here, $t_{1}=t_{\text{start}}=0$ years and $t_{n}=t_{\text{stop}}=$ 80 years. To reduce random errors and obtain a smooth estimate of $\bar{\gamma }$ over time, we adopted a weight kernel of the form

K(u)={12cos(πbu)+12;∣u∣≤b0;∣u∣>b (29)

where b denotes the full duration at half maximum (FDHM) of the kernel (fig. S20) and K(u)=0 for u<−b and u>b . The diagonal in Eq. 28 is then nonzero only when $|t_{i}-T|\leq b$ . In this capacity, b denotes the resolution of $\bar{\gamma }\left(T\right)$ , such that adjacent temporal structures closer together than b years are not properly resolved.

By fitting a simple weighted linear regression model to the cumulative effect estimates, $\bar{\gamma }$ is readily given by

$$\bar{\gamma }\left(T\right)={\left.\frac{d}{\mathrm{dt}},\left(\hat{\xi_{0}\left(T\right)}+\hat{\xi_{1}\left(T\right)}t\right),\;\right|}_{t=T}=\hat{\xi_{1}\left(T\right)}$$ (30)

where $\hat{\xi_{0}\left(T\right)}$ and $\hat{\xi_{1}\left(T\right)}$ are the least-squares estimates to the model. We note that if $t_{i},i=1,\ldots ,n$ , is equidistant and the adopted kernel is symmetric around t=T , which the kernel in Eq. 28 is except near the end points $t_{\text{start}}$ and $t_{\text{stop}}$ , the estimate in Eq. 30 is accurate to second order, such that

$$\begin{array}{c} \bar{\gamma }\left(T\right)=\hat{\xi_{1}\left(T\right)}={\frac{d}{\mathrm{dt}}\left.(,\hat{\xi_{0}^{\prime }\left(T\right)},+,\hat{\xi_{1}^{\prime }\left(T\right)},t,+,\hat{\xi_{2}^{\prime }\left(T\right)},t^{2},),\;\right|}_{t=T}\\ =\hat{\xi_{1}^{\prime }\left(T\right)}+2\hat{\xi_{2}^{\prime }\left(T\right)}\cdot T \end{array}$$ (31)

where $\hat{\xi^{\prime }\left(T\right)}={\left(\hat{\xi_{0}^{\prime }\left(T\right)},\hat{\xi_{1}^{\prime }\left(T\right)},\hat{\xi_{2}^{\prime }\left(T\right)}\right)}^{T}$ denotes the vector of parameter estimates to the model including a second-order term in time.

For the results presented in this study, we adopt a resolution (FDHM) of b=10 years. That is, the estimated effect corresponds to the momentaneous effect, averaged over 10 years. Although arbitrarily chosen, the adopted kernel width of b=10 years results in a “smoothed” momentaneous effect, where high-frequency random fluctuations are suppressed, while larger-scale temporal structures are retained. Other kernels, such as the box or Gaussian kernel (fig. S20), could, in principle, also be used. The kernel in Eq. 29 was adopted to keep good balance between bias and variance.

### Nonparametric estimation of the trend of Γ

To estimate the trend of Γ , we made use of the Hodrick-Prescott filter. This filtering technique is commonly adopted in macroeconomics for time-series decomposition to estimate long-term trends or to remove cyclical or seasonal components. It is a nonparametric smoothing technique and a special case of a smoothing spline. We use it here for noise reduction and to identify nonlinear trends in the time-varying effects. To this end, we used the hpfilter() function in the mFilter (v. 0.1-5) package in R. The smoothness penalty parameter λ was arbitrarily set to λ=50 to enable detection of long-term trends, while suppressing short-term fluctuations.

### Agglomerate hierarchical clustering of SNP effects

To identify potentially distinct time-dependent effects between different subgroups of SNPs, we applied the agglomerate hierarchical clustering procedure (*56*) implemented in the MR_AHC() function from the MRAHC (v. 0.1.0) package in R (https://github.com/xiaoran-liang/MRAHC). The method operates on summary statistics of the SNP effects on exposure and on outcome, together with corresponding standard errors of the effect estimates. We used default function settings, with minimum number of SNPs in a cluster set to four, and outlier removal procedure set to FALSE.

We included all unique BMI-associated SNPs identified in both subsamples of UKB. However, to increase precision in the clustering step, we reestimated effects and SEs using the full sample ( N=361,906 ). Summary statistics on T2DM (*57*) were taken from the DIAGRAM/DIAMANTE consortium (http://diagram-consortium.org/index.html), while effects on CAD were taken from a meta-analysis (*58*) of UKB and CARDIoGRAMplusC4D, downloaded via MR-Base (*10*). Further information about the outcome data summary statistics is provided in table S3. After disease-specific Steiger filtering and harmonization (*10*) such as effect-allele alignment, a total number of 149 SNPs for T2DM and 179 SNPs, excluding the four identified outlier SNPs, for CAD remained for cluster analysis (data S1). For each identified cluster, we then generated a unique BMI-PGS from the cluster-affiliated SNPs to be used as instrument for that cluster. To avoid overfitting, we constructed the cluster-specific PGSs using original weights, estimated in each subsample of UKB (see the “Steiger filtering and the generation of PGSs for BMI” section).

### Relative correction for selection into UKB

Let the probability $p_{i}$ , for individual i to opt out from participation in UKB, be given by

$$p_{i}=\Phi \left(d_{i}-\mu_{k}\right)$$

where Φ denotes the cumulative distribution function of the standard normal distribution, $d_{i}\in D$ denotes the standardized BMI measurement of individual i belonging to some set D , and $\mu_{k}$ denotes the mean of the normal distribution of the *k*th age stratum. As we do not know the characteristics of those individuals who actually opted out from UKB, we reused UKB participants to mimic opted-out individuals. To this end, we assigned the subset D of 33,871 participants below 45 years of age as reference. This subset was assumed to be least affected by selection. For each age stratum k , the parameter $\mu_{k}$ was determined by randomly selecting individuals from D given their probability $p_{i}$ , such that

$$|{\langle \text{BMI}-\text{PGS}\rangle }_{k,\text{corr}}-\left({\langle \text{BMI}-\text{PGS}\rangle }_{k,\text{orig}}+m_{k}\right)|<\varepsilon$$

where ${\langle \text{BMI}-\text{PGS}\rangle }_{k,\text{orig}}$ denotes the original mean BMI-PGS of the individuals in stratum k , ${\langle \text{BMI}-\text{PGS}\rangle }_{k,\text{corr}}$ denotes the mean BMI-PGS of the individuals in stratum k plus the individuals from D selected for stratum k , and $m_{k}$ denotes the relative expected offset $m_{k}$ . Here, $m_{k}$ is given by the expected mean BMI-PGS at 40 years minus the expected mean BMI-PGS at age stratum k (see fig. S16). Last, the parameter ε denotes a small, positive arbitrary number that governs the numerical tolerance. Since this is a single-parameter optimization problem, $\mu_{k}$ was determined by a simple table search. For each k , a set of new participants given by $\mu_{k}$ was thus appended to the existing UKB sample from the set D , such that the mean BMI-PGS was rectified (fig. S16). Note that except for being assigned a new age at assessment k , each newly selected participant appended to the existing UKB sample retained all their original data. The expanded UKB sample containing the original UKB sample plus the set of appended individuals was then used in the analysis as a UKB sample corrected for selection.

### Statistical analyses

Specific statistical analyses are discussed in the respective paragraph within Materials and Methods. The 95% CIs of the time-series trends for the cumulative and momentaneous effects were calculated using parametric bootstrap with 10,000 samples. New time-series data were generated by adding a random variate, $\varepsilon_{k}$ , to the effect estimate in each time point, such that

$${\hat{\Gamma }}_{\text{boot}}\left(t_{k}\right)=\hat{\Gamma }\left(t_{k}\right)+\varepsilon_{k}$$

where $\varepsilon_{k}∼𝒩\left(0,\sigma_{k}^{2}/\beta_{G}^{2}\left(t_{k}\right)\right)$ , assuming uncorrelated errors between time points. For each of the 10,000 bootstrapped time series, a new trend was fitted, and the 2.5 and 97.5% quantiles of the distribution of trend estimates were calculated in each time point.

Similarly, the 95% CIs of the mean and SD of BMI, stratified over both sex and age and depicted in fig. S18, were calculated using basic bootstrap with 1000 samples. Here, each bootstrap sample was generated by random resampling of individuals with replacement.

## Supplementary Materials

### The PDF file includes:

Supplementary Text Figs. S1 to S20 Tables S1 to S3 Legend for data S1 References
