# Supplementary material for: Time-resolved Mendelian randomization detects substantial variation in the detrimental effect of obesity throughout life
Source: Sci Adv. 2025 Oct 24;11(43):eadv0926. doi: 10.1126/sciadv.adv0926 (PMC12551696; doi:10.1126/sciadv.adv0926)
Supplement: Supplementary file 1 — Supplementary Text Figs. S1 to S20 Tables S1 to S3 Legend for data S1 References [file sciadv.adv0926_sm.pdf]

Science Advances
AAAS

Supplementary Materials for

Time-resolved Mendelian randomization detects substantial variation in the detrimental effect of obesity throughout life

Torgny Karlsson *et al.*

Corresponding author: Torgny Karlsson, torgny.karlsson@igp.uu.se; asa.johansson@igp.uu.se

Sci. Adv. **11**, eadv0926 (2025)
DOI: 10.1126/sciadv.adv0926

The PDF file includes:

Supplementary Text
Figs. S1 to S20
Tables S1 to S3
Legend for data S1
References

Other Supplementary Material for this manuscript includes the following:

Data S1

Supplementary Text

Summary of core assumptions of Mendelian randomization

A valid genetic instrument (Fig. S1) should satisfy three instrumental assumptions (*7,59*). The first assumption (i), referred to as the relevance condition, states that the genetic instrument should be strongly associated with the exposure. In the context of time-resolved MR, this generalizes to the instrument being strongly associated with the exposure, at all times. This implies that $\beta_G(t) \neq 0 \; \forall t$, and $\beta_G(t)$ must therefore not switch signs anytime during the life-course. The second assumption (ii), referred to as the exchangeability condition, states that the instrument and the outcome should not share any causes, i.e., there must be no confounding factors between instrument and outcome at any time. This condition may be violated in the presence of population stratification, assortative mating, or dynastic effects. The third assumption (iii), also called the exclusion restriction, states that the instrument should not affect the outcome except potentially through the exposure. This includes both direct pathways between instrument and outcome and indirect pathways through any other variable that acts as an ancestor to the outcome. When this condition is not met, a phenomenon known as horizontal pleiotropy arises.

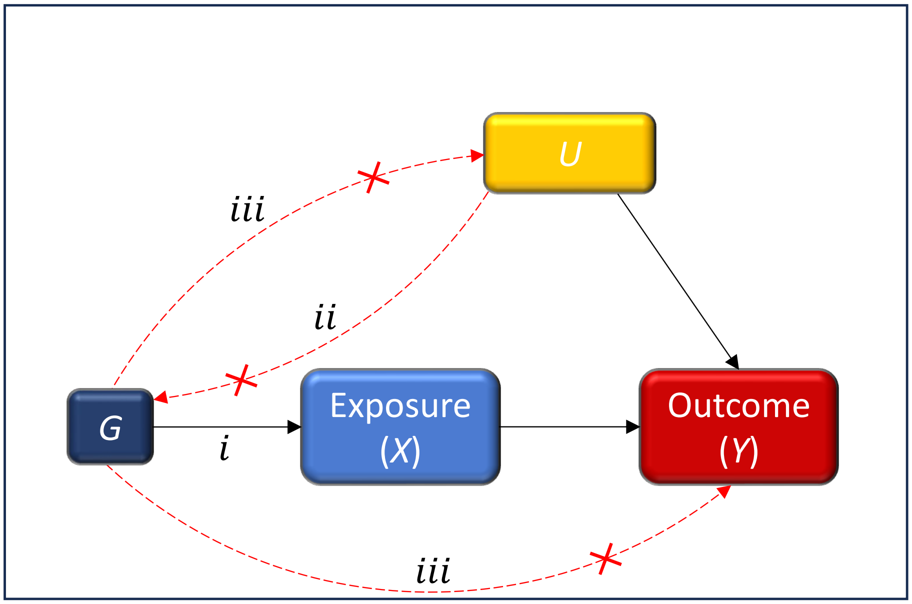

Figure S1. Causal diagram illustrating the three instrumental assumptions in Mendelian randomization. (i) The relevance condition states that the genetic instrument G should be associated with exposure X at all times. (ii) The exchangeability condition states that G and outcome Y should not share any causes U. (iii) The exclusion restriction states that any potential effect of G on outcome Y should be fully mediated through exposure X, alone. If horizontal pleiotropy is present, this condition is formally not met. Black, solid arrows show allowed or necessary pathways, while red, dashed arrows show forbidden pathways (crossed).

To enable a proper causal interpretation of the point estimate of the effect of the exposure on outcome, a fourth, identifying condition is necessary (*60*). For MR, the most relevant formulation of this identifying condition (iv) is commonly expressed as a monotonicity condition. Assume that a potential increase in the genetic instrument results in a change in exposure that always points in the same direction for all individuals, i.e., the effect is directionally uniform and there are no “defiers” in which the effect on exposure points in the opposite direction. If so, the estimated effect may be interpreted as the average causal effect of the exposure on outcome in those individuals whose exposure is changed by the instrument (*7*).

A generalized model allowing for the latent outcome to depend on time of measurement

In the present study, we assume that a prompt change in the exposure $x = x(t)$ exerted at time $t \leq T$, results in an instant and irreversible change in the latent outcome υ, described by the effect $\gamma = \gamma(t)$. That is, any change in biology induced by the exposure, has an instant effect on the risk of developing disease. However, if a change in x, exerted at time t, results in a delayed change in the latent outcome or in a change in the latent outcome that diminishes with time after exertion, the effect will also depend on what time T the outcome is measured.

To model a more general situation, let us define the manifested effect, $\delta = \delta(t, T)$, caused by x at time t, as the effect perceived at any later time $T > t$, where T being the time-point when the outcome is measured. Note that, for an instant and irreversible change in the latent outcome, the manifested effect $\delta(t, T)$, is equal to $\gamma(t), \forall\, T > t$. The general scenario may be modelled by modifying the expression (1, main text) for the latent outcome such that

$$\upsilon_i(t, T) = \gamma_0(t, T) + \delta(t, T) \cdot x_i(t) + u^{\upsilon}(u_i(t, T), t, T) + z^{\upsilon}(z_i(t, T), t, T) + \epsilon_i^{\upsilon}(t, T). \quad \text{(S1)}$$

Here, the manifested effect, $\delta(t, T)$, may be expressed as a generalized momentaneous effect of $x_i = x_i(t)$ on the latent outcome $\upsilon_i = \upsilon_i(t, T)$, caused by x_i at time t and perceived at time $T > t$, for individual i. If we instead insert (S1) in expression (3, main text) and expand $x_i(t)$ using (2, main text), we may extract a modified integral equation for H, such that

$$\mathrm{H}(T) = \int_0^T \delta(t, T) \cdot \beta_G(t) \mathrm{d}t, \quad \text{(S2)}$$

where $\gamma(t)$ in (5, main text) has been replaced by $\delta(t, T)$. We note that the integral equation in (S2), resembles a Volterra integral equation of the first kind. Generally, δ is an unknown function of (t, T), and will depend on disease outcome. The undefined dependence on T signifies our temporal ignorance – at a molecular level – of *when* and *to what degree* a momentaneous change in the exposure at t causes a change in the outcome at T.

To remedy the situation, let Λ denote some bounded function, such that $0 \leq \Lambda \leq 1$, and identify γ as the underlying momentaneous causal effect, exerted at t. Furthermore, let the manifested effect be given by

$$\delta(t, T) = \gamma(t) \cdot \Lambda(t, T). \quad \text{(S3)}$$

Hence, by inserting (S3) in (S2), we obtain

$$\mathrm{H}(T) = \int_0^T \gamma(t) \cdot \big(\Lambda(t, T) \cdot \beta_G(t)\big) \mathrm{d}t. \quad \text{(S4)}$$

Suppose now that we have prior information on the functional form of Λ, e.g., by biological insight and/or clinical knowledge. The only unknown in (S4) is then the sought-after causal effect $\gamma(t)$ which can be solved for, using numerical optimization procedures. Here, the function $\Lambda(t, T)$ will be denoted as the "carrier function", since it may be interpreted as a carrier of the effect $\gamma(t)$ of x, exerted at time t, into some future time of observation T. In this study, we let $\Lambda(t, T) = 1$, which implies that a prompt change in exposure at time t has an instant and irreversible effect on outcome.

Derivations of the variance of the estimator of the cumulative effect $\hat{\boldsymbol{\Gamma}}(\boldsymbol{t_k})$

Below, we outline the derivations of the variance expressions for the cumulative effects, used to enable inference of the point estimates, such as calculations of 95% confidence intervals. We derive expressions, both for the midpoint rule and for the trapezoidal rule.

Variance of $\hat{\Gamma}(t_k)$ *for the midpoint rule*

Recall that for the midpoint rule, the estimate of the cumulative effect of exposure on outcome is given by

$$\hat{\Gamma}(t_k) = \int_0^{t_k} \frac{\widehat{\mathrm{dH/dt}}}{\beta_G(t)} \mathrm{d}t \approx \sum_{i=1}^{k} \hat{\gamma}\left(t_{i-1/2}\right), \tag{S5}$$

where an estimate of the momentaneous effect is given by

$$\hat{\gamma}\left(t_{k-1/2}\right) = \frac{\widehat{\mathrm{dH}}}{\mathrm{d}t} \Big/ \beta_G\left(t_{k-1/2}\right) \approx \frac{\hat{\mathrm{H}}(t_k) - \hat{\mathrm{H}}(t_{k-1})}{\beta_G\left(t_{k-1/2}\right)}. \tag{S6}$$

Hence, the expression for the variance of $\hat{\Gamma}(t_k)$ may be expanded, using (S5) and (S6), to

$$\begin{aligned}\mathrm{Var}\left[\hat{\Gamma}(t_k)\right] = \mathrm{Var}\Bigg[&\frac{\hat{\mathrm{H}}(t_1) - \hat{\mathrm{H}}(t_0)}{\beta_G\left(t_{1/2}\right)} + \frac{\hat{\mathrm{H}}(t_2) - \hat{\mathrm{H}}(t_1)}{\beta_G\left(t_{3/2}\right)} + \cdots + \frac{\hat{\mathrm{H}}(t_{k-1}) - \hat{\mathrm{H}}(t_{k-2})}{\beta_G\left(t_{k-3/2}\right)} \\ &+ \frac{\hat{\mathrm{H}}(t_k) - \hat{\mathrm{H}}(t_{k-1})}{\beta_G\left(t_{k-1/2}\right)}\Bigg].\end{aligned} \tag{S7}$$

By rearranging the terms in (S7), we obtain

$$\begin{aligned}\mathrm{Var}\left[\hat{\Gamma}(t_k)\right] = \mathrm{Var}\Bigg[&-\frac{\hat{\mathrm{H}}(t_0)}{\beta_G\left(t_{1/2}\right)} + \left(\frac{\hat{\mathrm{H}}(t_1)}{\beta_G\left(t_{1/2}\right)} - \frac{\hat{\mathrm{H}}(t_1)}{\beta_G\left(t_{3/2}\right)}\right) + \cdots + \left(\frac{\hat{\mathrm{H}}(t_{k-1})}{\beta_G\left(t_{k-3/2}\right)} - \frac{\hat{\mathrm{H}}(t_{k-1})}{\beta_G\left(t_{k-1/2}\right)}\right) \\ &+ \frac{\hat{\mathrm{H}}(t_k)}{\beta_G\left(t_{k-1/2}\right)}\Bigg],\end{aligned} \tag{S8}$$

which can be rewritten as

$$\begin{aligned}\mathrm{Var}\left[\hat{\Gamma}(t_k)\right] = \mathrm{Var}\Bigg[&-\frac{\hat{\mathrm{H}}(t_0)}{\beta_G\left(t_{1/2}\right)} + \hat{\mathrm{H}}(t_1)\left(\frac{\beta_G\left(t_{3/2}\right) - \beta_G\left(t_{1/2}\right)}{\beta_G\left(t_{1/2}\right) \cdot \beta_G\left(t_{3/2}\right)}\right) + \cdots \\ &+ \hat{\mathrm{H}}(t_{k-1})\left(\frac{\beta_G\left(t_{k-1/2}\right) - \beta_G\left(t_{k-3/2}\right)}{\beta_G\left(t_{k-3/2}\right) \cdot \beta_G\left(t_{k-1/2}\right)}\right) + \frac{\hat{\mathrm{H}}(t_k)}{\beta_G\left(t_{k-1/2}\right)}\Bigg].\end{aligned} \tag{S9}$$

Given that all increments are uncorrelated and the variance of β_G can be neglected to first order, the expression in (S9) may be written as

$$\mathrm{Var}\big[\hat{\Gamma}(t_k)\big] = \sigma_1^2 \frac{\big(\beta_G(t_{3/2}) - \beta_G(t_{1/2})\big)^2}{\beta_G^2(t_{1/2}) \cdot \beta_G^2(t_{3/2})} + \cdots + \sigma_{k-1}^2 \frac{\big(\beta_G(t_{k-1/2}) - \beta_G(t_{k-3/2})\big)^2}{\beta_G^2(t_{k-3/2}) \cdot \beta_G^2(t_{k-1/2})} + \frac{\sigma_k^2}{\beta_G^2(t_{k-1/2})}$$
$$\approx \frac{\sigma_k^2}{\beta_G^2(t_{k-1/2})} \approx \frac{\sigma_k^2}{\beta_G^2(t_k)}, \qquad \text{(S10)}$$

using the notation $\mathrm{Var}\big[\hat{\mathrm{H}}(t_k)\big] = \sigma_k^2$ in (21, main text). Here, we have assumed that $\hat{\mathrm{H}}(t_0 = 0) = 0$ and that all terms quadratic in $\beta_G(t_j) - \beta_G(t_{j-1})$ can be neglected. This approximation should be acceptable, as long as the change in β_G between two consecutive years is small. Similarly, the last approximation assumes that $\beta_G^2(t_{k-1/2}) \approx \beta_G^2(t_k)$, which is made to simplify the expression.

Variance of $\hat{\Gamma}(t_{k-1/2})$ for the trapezoidal rule

Recall that, for the trapezoidal rule, we have that

$$\hat{\Gamma}(t_{k-1/2}) = \widehat{\int_0^{t_{k-1/2}} \frac{\mathrm{dH/dt}}{\beta_G(t)} \mathrm{d}t} \approx \frac{\Delta t \cdot \hat{\gamma}(t_{1/2})}{4} + \frac{\Delta t \cdot \hat{\gamma}(t_{1/2})}{2} + \Delta t \cdot \sum_{i=2}^{k-1} \hat{\gamma}(t_{i-1/2}) + \frac{\Delta t \cdot \hat{\gamma}(t_{k-1/2})}{2}$$
$$= \frac{1}{4}\hat{\gamma}(t_{1/2}) + \frac{1}{2}\hat{\gamma}(t_{1/2}) + \sum_{i=2}^{k-1} \hat{\gamma}(t_{i-1/2}) + \frac{1}{2}\hat{\gamma}(t_{k-1/2}), \qquad \text{(S11)}$$

for an interior point $t_{k-1/2}, k \geq 2$. Here, we have assumed that $\Delta t = 1$ in the last equality. Assuming that $\hat{\gamma}(t_0 = 0) = 0$, the first term denotes an approximation of the integral in the interval $[0, t_{1/2}] = [0, 1/2]$. Now, for an interior point $t_{k-1/2}, k \geq 3$, we have that

$$\mathrm{Var}\big[\hat{\Gamma}(t_{k-1/2})\big] = \mathrm{Var}\left[\Delta t \left(\frac{\hat{\mathrm{H}}(t_1) - \hat{\mathrm{H}}(t_0)}{4 \cdot \beta_G(t_{1/2})}\right)\right.$$
$$+ \Delta t \left(\frac{\hat{\mathrm{H}}(t_1) - \hat{\mathrm{H}}(t_0)}{2 \cdot \beta_G(t_{1/2})} + \frac{\hat{\mathrm{H}}(t_2) - \hat{\mathrm{H}}(t_1)}{\beta_G(t_{3/2})} + \frac{\hat{\mathrm{H}}(t_3) - \hat{\mathrm{H}}(t_2)}{\beta_G(t_{5/2})} \ldots + \frac{\hat{\mathrm{H}}(t_{k-1}) - \hat{\mathrm{H}}(t_{k-2})}{\beta_G(t_{k-3/2})}\right.$$
$$\left.\left. + \frac{\hat{\mathrm{H}}(t_k) - \hat{\mathrm{H}}(t_{k-1})}{2 \cdot \beta_G(t_{k-1/2})}\right)\right]$$
$$= \Delta t^2 \cdot \mathrm{Var}\left[-\frac{3 \cdot \hat{\mathrm{H}}(t_0)}{4 \cdot \beta_G(t_{1/2})} + \hat{\mathrm{H}}(t_1)\left(\frac{3 \cdot \beta_G(t_{3/2}) - 4 \cdot \beta_G(t_{1/2})}{4 \cdot \beta_G(t_{1/2}) \cdot \beta_G(t_{3/2})}\right)\right.$$
$$+ \hat{\mathrm{H}}(t_2)\left(\frac{\beta_G(t_{5/2}) - \beta_G(t_{3/2})}{\beta_G(t_{3/2}) \cdot \beta_G(t_{5/2})}\right) + \cdots + \hat{\mathrm{H}}(t_{k-1})\left(\frac{2 \cdot \beta_G(t_{k-1/2}) - \beta_G(t_{k-3/2})}{2 \cdot \beta_G(t_{k-3/2}) \cdot \beta_G(t_{k-1/2})}\right)$$
$$\left. + \frac{\hat{\mathrm{H}}(t_k)}{2 \cdot \beta_G(t_{k-1/2})}\right]. \qquad \text{(S12)}$$

Similar to above, we assume that $\Delta t = 1$, $\hat{\mathrm{H}}(t_0 = 0) = 0$, and that all terms quadratic in $\beta_G(t_{j+1/2}) - \beta_G(t_{j-1/2})$ have a negligible contribution to the variance. However, in contrast to the expression in (S9), there are now three non-negligible terms in (S12), such that

$$\mathrm{Var}\left[\hat{\Gamma}\left(t_{k-1/2}\right)\right] \approx \sigma_1^2 \frac{\left(3 \cdot \beta_G\left(t_{3/2}\right) - 4 \cdot \beta_G\left(t_{1/2}\right)\right)^2}{16 \cdot \beta_G^2\left(t_{1/2}\right) \cdot \beta_G^2\left(t_{3/2}\right)} + \sigma_{k-1}^2 \frac{\left(2 \cdot \beta_G\left(t_{k-1/2}\right) - \beta_G\left(t_{k-3/2}\right)\right)^2}{4 \cdot \beta_G^2\left(t_{k-3/2}\right) \cdot \beta_G^2\left(t_{k-1/2}\right)} + \frac{\sigma_k^2}{4 \cdot \beta_G^2\left(t_{k-1/2}\right)} \approx \frac{\sigma_1^2}{16 \cdot \beta_G^2(t_1)} + \frac{\sigma_{k-1}^2}{4 \cdot \beta_G^2(t_{k-1})} + \frac{\sigma_k^2}{4 \cdot \beta_G^2(t_k)}, \quad \text{(S13)}$$

where we have assumed that $\beta_G\left(t_{j-1/2}\right) \approx \beta_G\left(t_j\right) \approx \beta_G\left(t_{j+1/2}\right)$.

Simulations

Main simulation for validation of the time-resolved MR method

We performed simulations to illustrate that a time-dependent effect can correctly be retrieved also for a time-dependent instrument, using time-resolved MR, while a naïve use of the Wald ratio leads to biased results. For the main simulations, we assumed that the effect per unit higher value in exposure per unit of time on the latent outcome followed a power-law in time, such that $\gamma(t) \propto t^4$. This implies that the cumulative, life-course effect should increase with time to the power of five, such that

$$\Gamma(T) \propto T^5, \quad \text{(S14)}$$

which is strongly time-dependent. Let the data-generating model formally be given by the expressions for the exposure $x(t)$ and the latent outcome $\upsilon(t)$

$$x(t) = \beta_0(t) + \beta_G(t) \cdot G + \beta_u(t) \cdot u + \beta_z(t) \cdot z, \quad \text{(S15)}$$
$$\upsilon(t) = \gamma_0(t) + \gamma(t) \cdot x(t) + \gamma_u(t) \cdot u + \gamma_z(t) \cdot z. \quad \text{(S16)}$$

The observed outcome is then given by

$$\Upsilon(T) = \int_0^T \upsilon(t)\mathrm{d}t + C_0 = \int_0^T \gamma_0(t) + \gamma(t) \cdot x(t) + \gamma_u(t) \cdot u + \gamma_z(t) \cdot z\mathrm{d}t + C_0 = \int_0^T \gamma_0(t)\mathrm{d}t + \int_0^T \gamma(t) \cdot x(t)\mathrm{d}t + \int_0^T \gamma_u(t) \cdot u\mathrm{d}t + \int_0^T \gamma_z(t) \cdot z\mathrm{d}t + C_0, \quad \text{(S17)}$$

which can be rewritten to

$$\Upsilon(T) = \int_0^T \gamma(t) \cdot x(t)\mathrm{d}t + \Gamma_0(T) + \Gamma_u(T) \cdot u + \Gamma_z(T) \cdot z + C_0, \quad \text{(S18)}$$

where the first term in (S18) may be written as

$$\int_0^T \gamma(t)\cdot x(t)\mathrm{d}t = \int_0^T \gamma(t)\cdot(\beta_0(t)+\beta_G(t)\cdot G+\beta_u(t)\cdot u+\beta_z(t)\cdot z)\,\mathrm{d}t$$
$$= \mathrm{H}(T)\cdot G+\mathrm{H}_0(T)+\mathrm{H}_u(T)\cdot u+\mathrm{H}_z(T)\cdot z. \qquad \text{(S19)}$$

Without loss of generality, we have assumed that u and z are time-independent with $u \sim \Gamma(1,1)$ and $z \sim \Gamma(10,1)$ being gamma-distributed, while their effects on x, γ and Γ are time-dependent. Furthermore, the genetic instrument is assumed to be dichotomous following a binomial distribution, such that $G \sim Bin(1,p)$ with success probability $p = 0.4$. To ensure a positive rate at all times, we set $C_0 = 1 \times 10^{-8}$.

Functional forms of the time-dependences of the specific parameters in the data-generating model for the main simulation (Fig. 2, main text) are given in Supplementary Table S1. These functional forms were specifically chosen to give an exposure that numerically resembled BMI in UKB. For example, the mean and standard deviation of the exposure between 40 and 70 years of age were 26.4 units and 4.6 units, respectively, which are close to the mean and standard deviation of BMI in UKB. Furthermore, the set of parameters in Supplementary Table S1 resulted in an outcome prevalence of 15.0% in the population between 0 and 80 years of age, somewhere in between the prevalence of coronary artery disease and osteoarthritis, which are both common diseases in the UKB (Table 1, main text). In the simulations, we assumed a number of individuals $N = 300{,}000$ to match the number of participants in our study.

To model the hazard rate, we first transformed $\Upsilon_i(T)$ in (S18) for each individual i into a probability

$$p_i(T) = 1 - e^{-\Upsilon_i(T)}. \qquad (S20)$$

Whether or not individual i was diagnosed with disease at age T was then determined by a negative binomial with success rate $p_i(T)$, such that $D_i \sim NBin\big(r = 1, p_i(T)\big)$, where r denotes the number of successes. Any individual with zero failures before first ($r = 1$) success at age T, i.e., any individual with $D_i = 0$ at T, was then said to be diagnosed with disease at T. This procedure was repeated for all ages $T \in \{0,1,\ldots,80\}$. Once diagnosed with the disease, the individual was removed from the risk set.

We compared the time-dependent exposure effect on outcome, estimated by time-resolved MR, with the time-dependent effects, estimated by standard MR where we made a naïve use of the Wald ratio. This was done in the following way: First, we estimated the effect of the instrument on exposure and on outcome for the simulated data, in the same way as described in the main text. The effect of the instrument on exposure was estimated using linear regression. A constant (time-invariant) effect was estimated from a mis-specified model including only G and a fourth-order polynomial in age, while a time-dependent (quartic) effect was estimated from a correctly specified model including G, $G \times$ age, $G \times$ age^4, and an additional fourth-order polynomial in age (*cf.* Fig. 2D, E). The time-varying effect of the instrument on outcome was estimated using Aalen's additive hazard model, including G as a single covariate. Second, the output from the models was used to estimate the time-dependent effect of exposure on outcome. We adopted three different MR estimation methods, of which two generated biased results. In methods A and B, we made a naïve use of the Wald ratio and simply divided the instrument effect on outcome with the instrument effect on exposure. The instrument effect on exposure was either taken from the mis-specified regression model (method A: time-fixed β_G) or from the correctly specified model (method B: time-dependent β_G). In method C, we used time-resolved MR to estimate the time-dependent effect of exposure on outcome.

To mimic the error and dependence between adjacent time-points in the results from time-resolved MR using the trapezoidal rule, we calculated the mean estimated effect, $\hat{\Gamma}(t_{k-1/2})$, at $t = k - 1/2$ of the two adjacent points in $t = k - 1$ and $t = k$, for the two MR methods A and B. That is,

$$\hat{\Gamma}(t_{k-1/2}) = \frac{\hat{\Gamma}(t_{k-1}) + \hat{\Gamma}(t_k)}{2}, k = 1, \ldots, 80. \tag{S21}$$

The corresponding variance of the estimate at $t_{k-1/2}$ was given by

$$\mathrm{Var}[\hat{\Gamma}(t_{k-1/2})] = \frac{1}{4}\left(\mathrm{Var}[\hat{\Gamma}(t_{k-1})] + \mathrm{Var}[\hat{\Gamma}(t_k)]\right). \tag{S22}$$

As for the numerical effect estimates, the theoretical (biased) effects in methods A and B were calculated using the (naïve) Wald ratio. Here, the numerator was given by

$$\mathrm{H}(T) = \int_0^T \gamma(t) \cdot \beta_G(t) \mathrm{d}t \tag{S23}$$

in both methods A and B. Note that, the expression in (S23) depends on $\beta_G(t)$ at all past time-points $t \leq T$. The denominator in method A was represented by the mean, time-averaged instrument effect on exposure

$$\langle \beta_G \rangle = \int_0^{T_{\max}} \beta_G(t) \mathrm{d}t \Big/ T_{\max}, \tag{S24}$$

while the denominator in method B was represented by the time-dependent instrument effect on exposure evaluated at time T, i.e., $\beta_G(T)$. Hence, the Wald ratios were given by

$$\text{Method A: } \mathrm{H}(T)/\langle \beta_G \rangle \text{ and} \tag{S25}$$
$$\text{Method B: } \mathrm{H}(T)/\beta_G(T), \tag{S26}$$

respectively. Clearly, the cumulative effect $\Gamma(T) = \int_0^T \gamma(t)\mathrm{d}t$ cannot be extracted from (S23), neither by dividing by $\langle \beta_G \rangle$ in (S25), nor by $\beta_G(T)$ in (S26).

The main simulations are presented in Fig. 2 (main text), while additional simulations are presented in Supplementary Fig. S4.

PGS constructed from SNPs with different time-dependences

In a secondary set of simulations, we tested whether the estimated time-dependent effect of a variable given by the sum of three individual instruments with widely diverse time-dependences also is a valid, time-dependent instrument. Here, we assumed that the three individual instruments $G_1 \sim Bin(2,0.1)$, $G_2 \sim Bin(2,0.4)$, and $G_3 \sim Bin(2,0.2)$ were independent genetic variants with different minor alleles frequencies. Similar to Eq. (S15), the exposure was given by

$$x(t) = \beta_0(t) + \beta_1(t) \cdot G_1 + \beta_2(t) \cdot G_2 + \beta_3(t) \cdot G_3 + \beta_u(t) \cdot u + \beta_z(t) \cdot z,$$

with three genetic instruments, however, instead of a single instrument. The three time-dependent coefficients were set to $\beta_1(t) = 0.2 + 0.001t$, $\beta_2(t) = 0.3 - 0.003t$, and $\beta_3(t) = 0.2 + 0.002t - 8 \times 10^{-9}t^4$ (Fig. S5). Otherwise, all variables and parameters were the same as in the main simulation (Fig. 2, Table S1). To mimic a PGS, we generated an unweighted sum of the three individual variants, such that $G = G_1 + G_2 + G_3$. The time-dependent coefficient of the PGS, given the time-dependences of the individual variants and their specific minor allele frequencies, was estimated to

$$\beta_G(t) = 0.249 - 0.000642t - 2.599 \times 10^{-9}t^4,$$

assuming a quartic model in time. We used this PGS as instrument in the time-resolved MR analysis, with $\beta_G(t)$ describing the time-dependent effect on exposure. The results show that a PGS computed from individual SNPs with uniquely different time-dependences is also a valid, time-dependent instrument, as illustrated in Fig. S5.

Pathway-specific effect of sustained high BMI using agglomerate hierarchical clustering

We examined the possible existence of multiple causal pathways between BMI and the two cardiometabolic diseases T2DM and CAD. To this end, we generated cluster-specific PGSs, where the partitioning of BMI-associated SNPs into distinct clusters was performed for each disease separately, using agglomerate hierarchical clustering (*56*), as described in Materials and Methods.

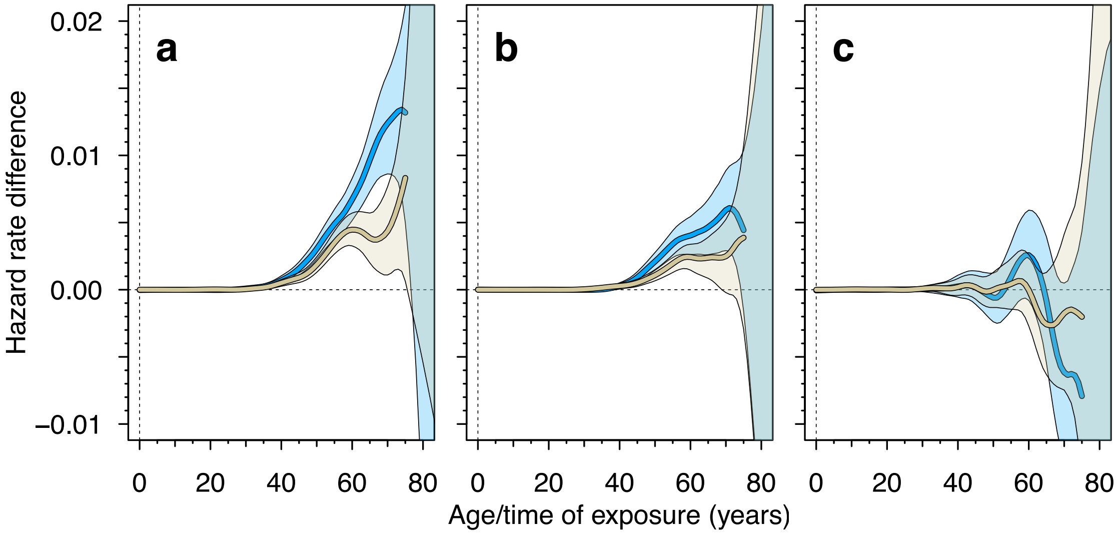

Figure S2. Sex-stratified trends for the three identified causal pathways between BMI and T2DM. Time-series trends were estimated from raw cumulative effects using Hodrick-Prescott filtering with $\lambda = 50$ (Materials and Methods). Results for females are denoted in beige, while results for males are denoted in blue. Parametric bootstrap with 10,000 bootstrap samples was used to calculate the 95% CIs. **a**) Trends of the cumulative effects for the high-risk pathway. **b**) Trends of the cumulative effects for the medium-risk pathway. **c**) Trends of the cumulative effects for the protective pathway. BMI = body mass index, T2DM = type 2 diabetes mellitus, CI = confidence interval.

For T2DM, hierarchical clustering partitioned SNPs into three different clusters, two clusters for which a one-unit higher BMI increased the odds of T2DM while the third cluster indicated a protective effect (Fig. S15). This agrees with previous studies that have identified both concordant and discordant effects of obesity-associated genetic variants on the risk of T2DM (*37, 39, 41*). The corresponding life-course effects showed unique temporal trends for the three identified clusters (Fig. S15), with the protective pathway being significantly negative at about the same time as the appearance of the trough in females

(Fig. 5A, main text). However, as demonstrated from sex-stratified analyses (Fig. S2), the high-risk pathway was found to exhibit the most pronounced difference in trends between sexes, where a trough in females but not in males was observed. This suggests that the sex-difference that was recognized in the time-dependent effect of BMI on T2DM is coupled to the high-risk pathway, rather than the protective pathway. The corresponding clustering results for CAD (Fig. S15) also indicate that the characteristic trough found in both sexes may be predominantly linked to the high-risk pathway, albeit the medium-risk pathway also displays tendencies of a trough. These results are intriguing and requires further investigation.

Finally, we note that significant differences in time-dependence exist among the cluster instruments (Fig. S3). If selection of genetic instruments would be based merely on the strength of their time-dependence as previously suggested (*11*), one should be aware that this could lead to preferential sampling of specific pathways.

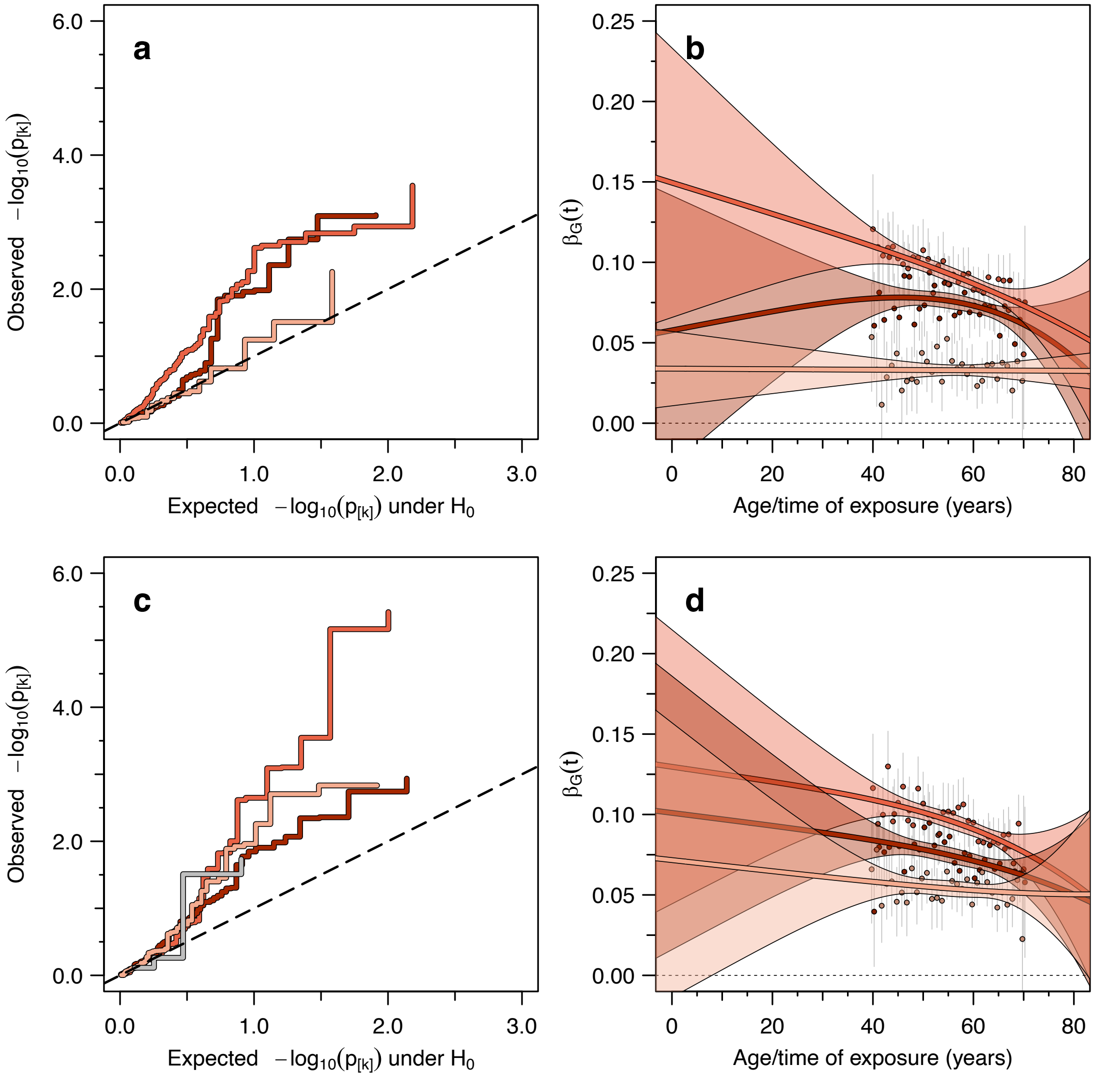

Figure S3. Time-dependence of cluster-specific SNPs for T2DM and CAD. a) QQ-plot for one-tailed *P*-values (H_1: $\beta_{\text{linear}} \times \text{sgn}(\beta_{\text{main effect}}) < 0$) of the linear interaction-effect estimates (β_{linear}) of cluster-specific SNPs identified in T2DM (Fig. S15). The dark, ruby red curve denotes partitioned SNPs belonging to the high-risk pathway, while the medium red curve denotes SNPs belonging to the medium-risk pathway. The curve for the protective pathway is

displayed in light rose red. The number of low P-values corresponding to negative time-interaction effects is enhanced in the two pathways showing an increased risk, but not in the protective pathway. **b**) Time-dependent effects on BMI of the corresponding PGSs, generated from each cluster-specific set of SNPs identified in T2DM. $\beta_G(t)$ of the two increased-risk pathways are strongly time-dependent, while $\beta_G(t)$ of the protective pathway show little evidence of time variation. **c**) QQ-plot for one-tailed P-values (H_1: $\beta_{\text{linear}} \times \text{sgn}(\beta_{\text{main effect}}) < 0$) of cluster-specific SNPs identified in CAD (Fig. S15). The dark, ruby red curve denotes partitioned SNPs belonging to the high-risk pathway, the medium red curve denotes SNPs belonging to the medium-risk pathway, the light rose red curve denotes SNPs belonging to the protective pathway, while the grey curve denotes outlier-SNPs removed from analysis. In contrast to the clusters identified in T2DM, all CAD pathways appear to be enriched in SNPs with negative time-interaction effects. **d**) Time-dependent effects on BMI of the PGSs, generated from each cluster-specific set of SNPs identified in CAD.

Supplementary Figures

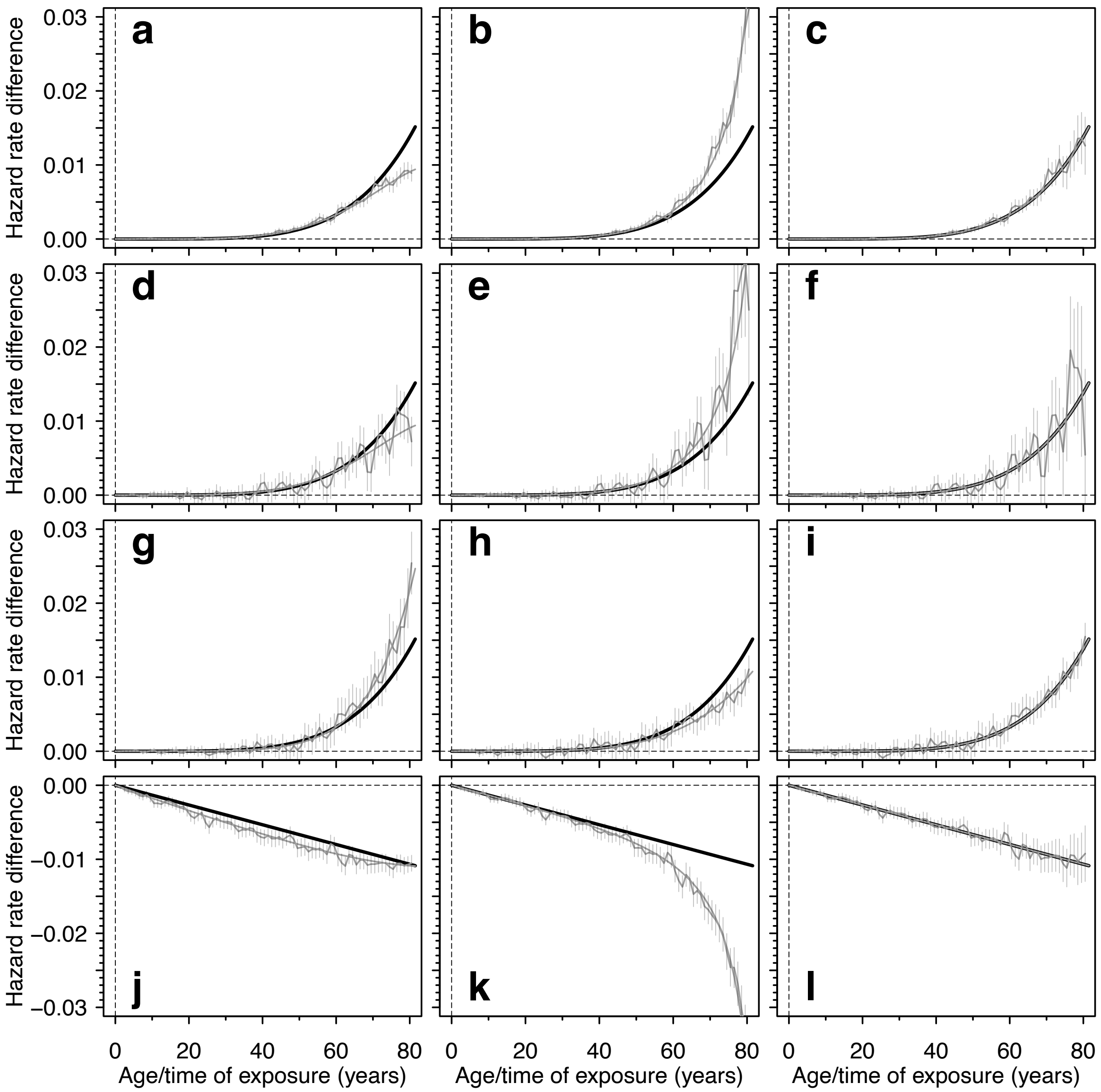

Figure S4. Simulations of time-dependent effects with time-dependent instruments. Each row signifies a different simulation while each column shows the results for the three different MR estimation methods (A, B, and C). Panels **a**-**c**) show results from a simulation with an outcome prevalence of 3.0%. **a**) Biased estimation (grey, connected points with 95% CI) of the non-linear ($\propto T^5$) cumulative effect (black, thick line), using method A (time-fixed estimate of β_G). The biased effect is denoted by the grey curve. **b**) Biased estimation (grey, connected points with 95% CI) of the cumulative effect (black, thick line), using method B (time-dependent estimate of β_G). The biased effect is denoted by the grey curve. **c**) Unbiased estimation (grey, connected points with 95% CI) of the cumulative effect (black, thick line) using time-resolved MR. No theoretical bias is present (grey curve). Panels **d**-**f**) show results from a simulation with an outcome prevalence of 30.0%. **d**) Biased estimation (grey, connected points with 95% CI) of the non-linear ($\propto T^5$) cumulative effect (black, thick line), using method A (time-fixed estimate of β_G). The biased effect is denoted by the grey curve. **e**) Biased estimation (grey, connected points with 95% CI) of the cumulative effect (black, thick line), using method B (time-dependent estimate of β_G). The biased effect is denoted by the grey curve. **f**) Unbiased estimation (grey, connected points with 95% CI) of the cumulative effect (black, thick line) using time-resolved MR. No theoretical bias is present (grey curve). Panels **g**-**i**) show results from

a simulation with an instrument effect on exposure that increases with age, such that $\beta_G(t) = 0.064 + 9 \times 10^{-4}t + 4 \times 10^{-9}t^4$, $t \in [0,80]$. The outcome prevalence was set to 15.0%. **g**) Biased estimation (grey, connected points with 95% CI) of the non-linear ($\propto T^5$) cumulative effect (black, thick line), using method A (time-fixed estimate of β_G). The biased effect is denoted by the grey curve. **h**) Biased estimation (grey, connected points with 95% CI) of the cumulative effect (black, thick line), using method B (time-dependent estimate of β_G). The biased effect is denoted by the grey curve. **i**) Unbiased estimation (grey, connected points with 95% CI) of the cumulative effect (black, thick line) using time-resolved MR. No theoretical bias is present (grey curve). Panels **j-l**) show results from a simulation with a linear ($\propto T$), protective exposure effect on outcome, a $\beta_G(t)$ that decreases with increasing age (Supplementary Table S1), and an outcome prevalence of 15.0%. **j**) Biased estimation (grey, connected points with 95% CI) of the protective, linear ($\propto T$) cumulative effect (black, thick line), using method A (time-fixed estimate of β_G). The biased effect is denoted by the grey curve. **k**) Biased estimation (grey, connected points with 95% CI) of the cumulative effect (black, thick line), using method B (time-dependent estimate of β_G). The biased effect is denoted by the grey curve. **l**) Unbiased estimation (grey, connected points with 95% CI) of the cumulative effect (black, thick line) using time-resolved MR. No theoretical bias is present (grey curve). CI = confidence interval, MR = Mendelian randomization.

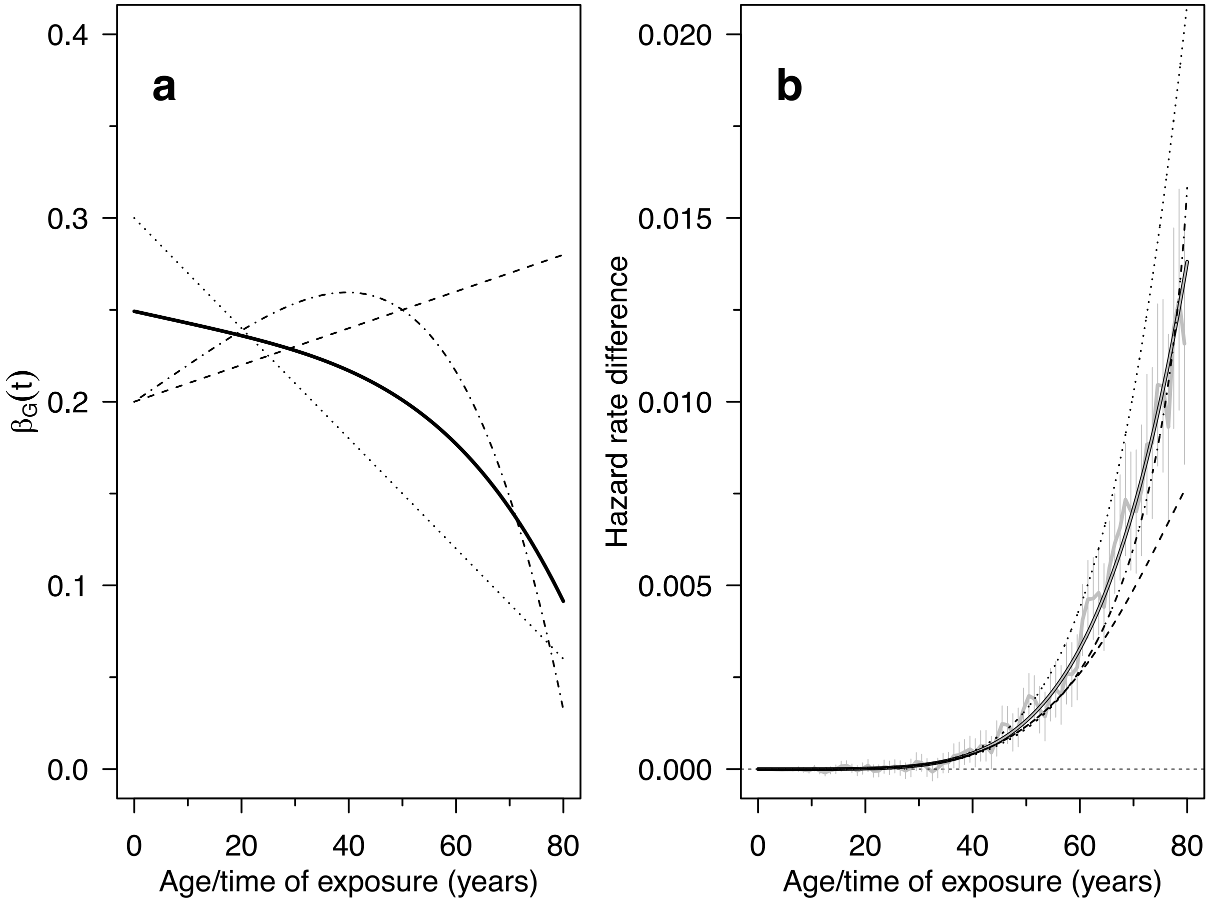

Figure S5. Simulation of a PGS constructed from SNPs with diverse time-dependences. In this simulation, we tested whether the PGS is a valid, time-dependent instrument given that it is generated by a sum of individual SNPs with widely diverse time-dependences. **a**) Adopted time-dependences of the individual SNPs (dashed, dotted, and dash-dotted lines). See Supplementary Materials for further details. The resulting time-dependence of the corresponding PGS is denoted by the thick, sold line. **b**) The cumulative effect estimated by time-resolved MR is denoted by the grey connected points with 95% CIs (grey bars), adopting the time-dependence of the PGS. The black/grey think curve denotes the true effect, while the dashed, dotted, and dash-dotted thin curves denote theoretical biased results, where the time-dependence of the PGS instrument is wrongly assumed to follow the time-dependence of each of the individual SNPs, shown to the left. PGS = polygenic score, SNP = single nucleotide polymorphism, MR = Mendelian randomization, CI = confidence interval.

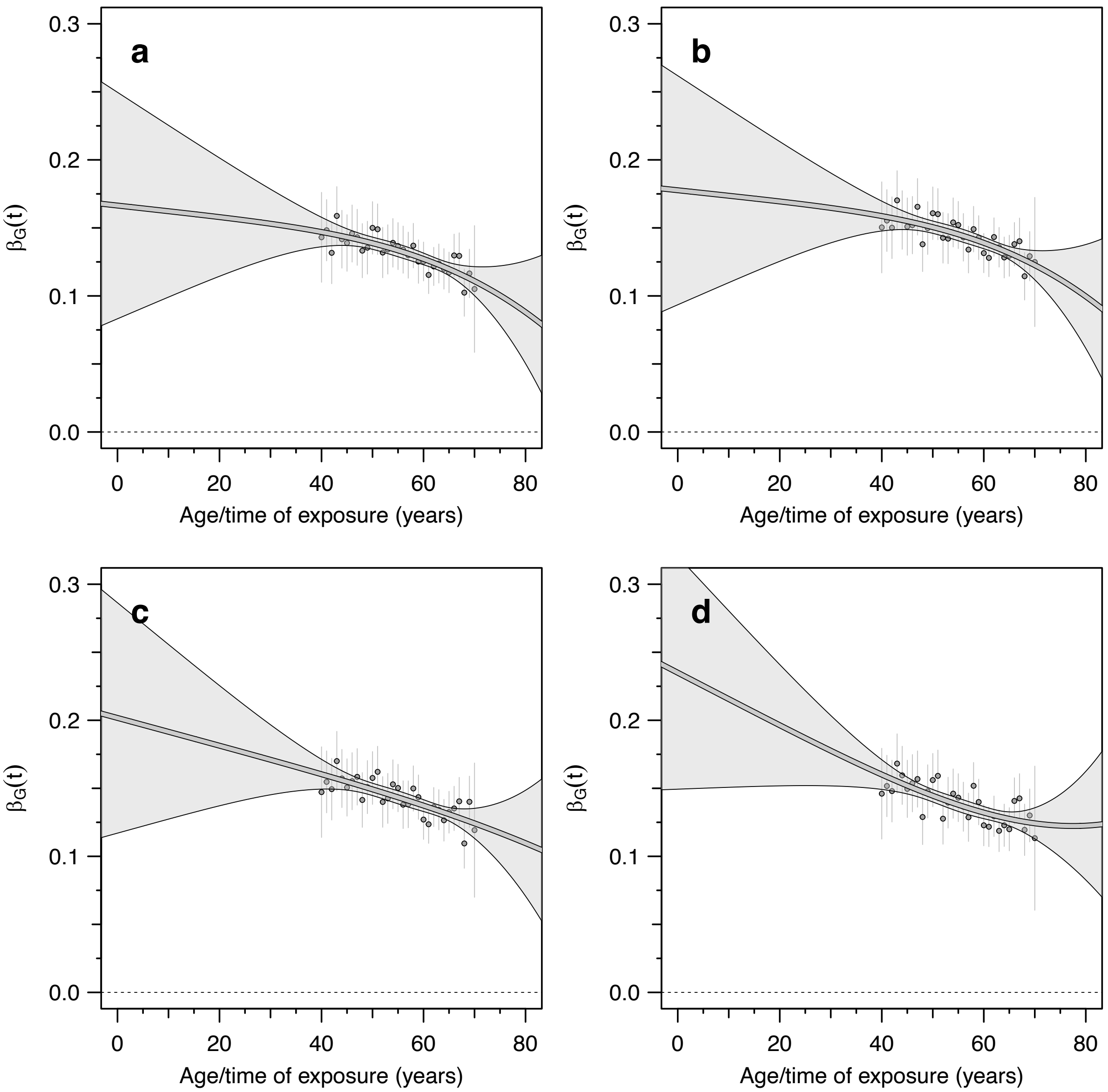

Figure S6. Time-dependence of the PGS for each disease, in all individuals. Time-dependent effects, $\beta_{\mathrm{G}}(t)$, of the disease-specific polygenic scores on BMI. The time-dependences, with corresponding 95% CIs, are determined using quartic fits to the data (Materials and Methods). Grey circles denote time-fixed estimates stratified on age. **a**) Time-dependent effect on BMI for T2DM, **b**) for atrial fibrillation, **c**) for CAD, and **d**) for osteoarthritis. PGS = polygenic score, BMI = body mass index, T2DM = type 2 diabetes mellitus, CAD = coronary artery disease.

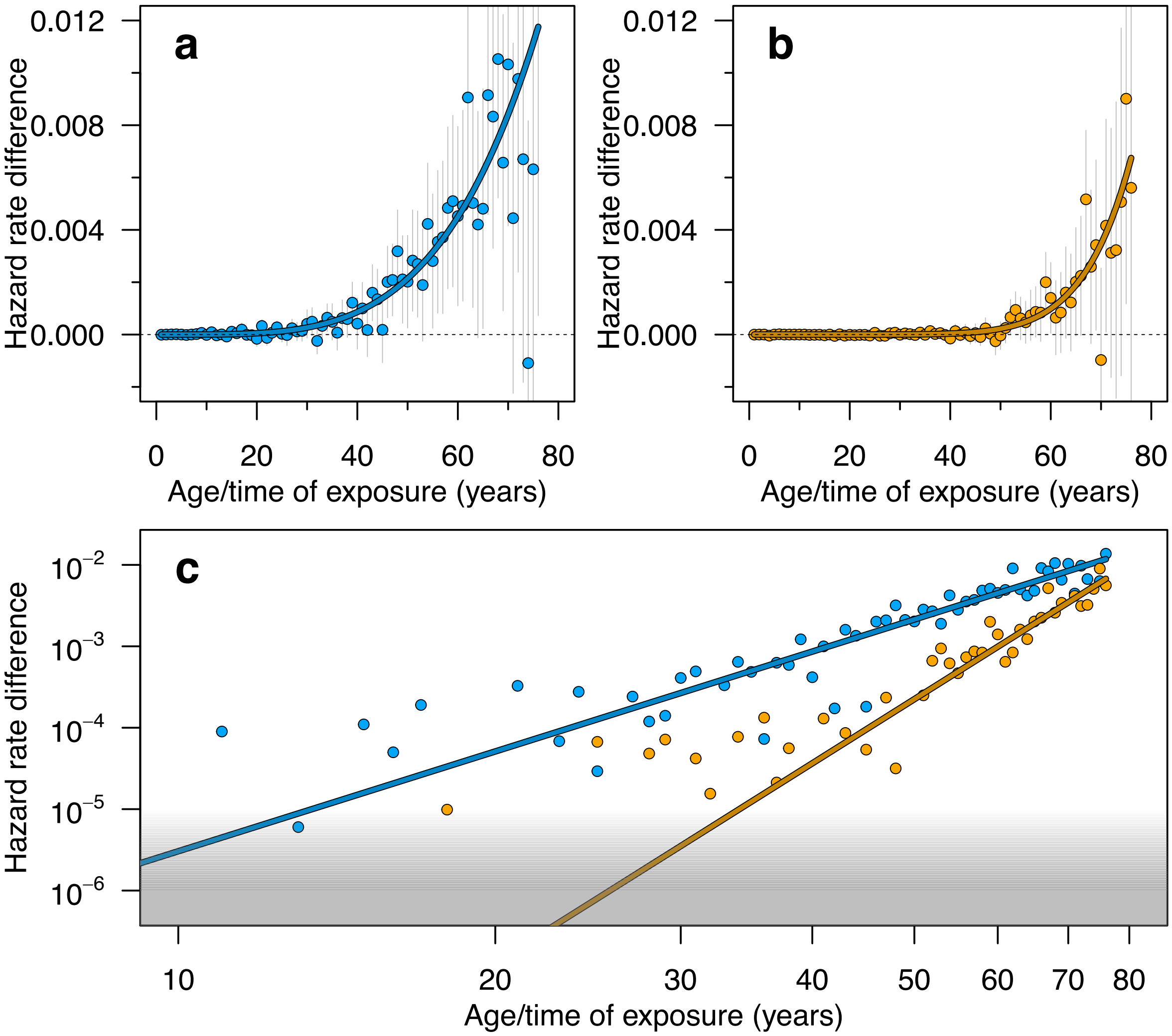

Figure S7. Power-law fits to the cumulative effects on osteoarthritis and atrial fibrillation. a) Blue filled circles denote the uncorrelated effect estimates (with 95% CI) for osteoarthritis (*cf.* Fig. 4, main text) using the midpoint rule (Materials and Methods). The blue, thick line is a power-law fit to the effect estimates. **b**) Yellow filled circles denote the uncorrelated effect estimates (with 95% CI) for atrial fibrillation (*cf.* Fig. 4, main text) using the midpoint rule (Materials and Methods). The yellow, thick line is a power-law fit to the effect estimates. **c**) Log-log plot of the power-law fits, with corresponding effect estimates. The rate of increase of the cumulative effect is higher for atrial fibrillation (yellow) compared with osteoarthritis (blue), as evident from the steeper slope of the fitted yellow line, indicating a larger exponent of the power-law. CI = confidence interval.

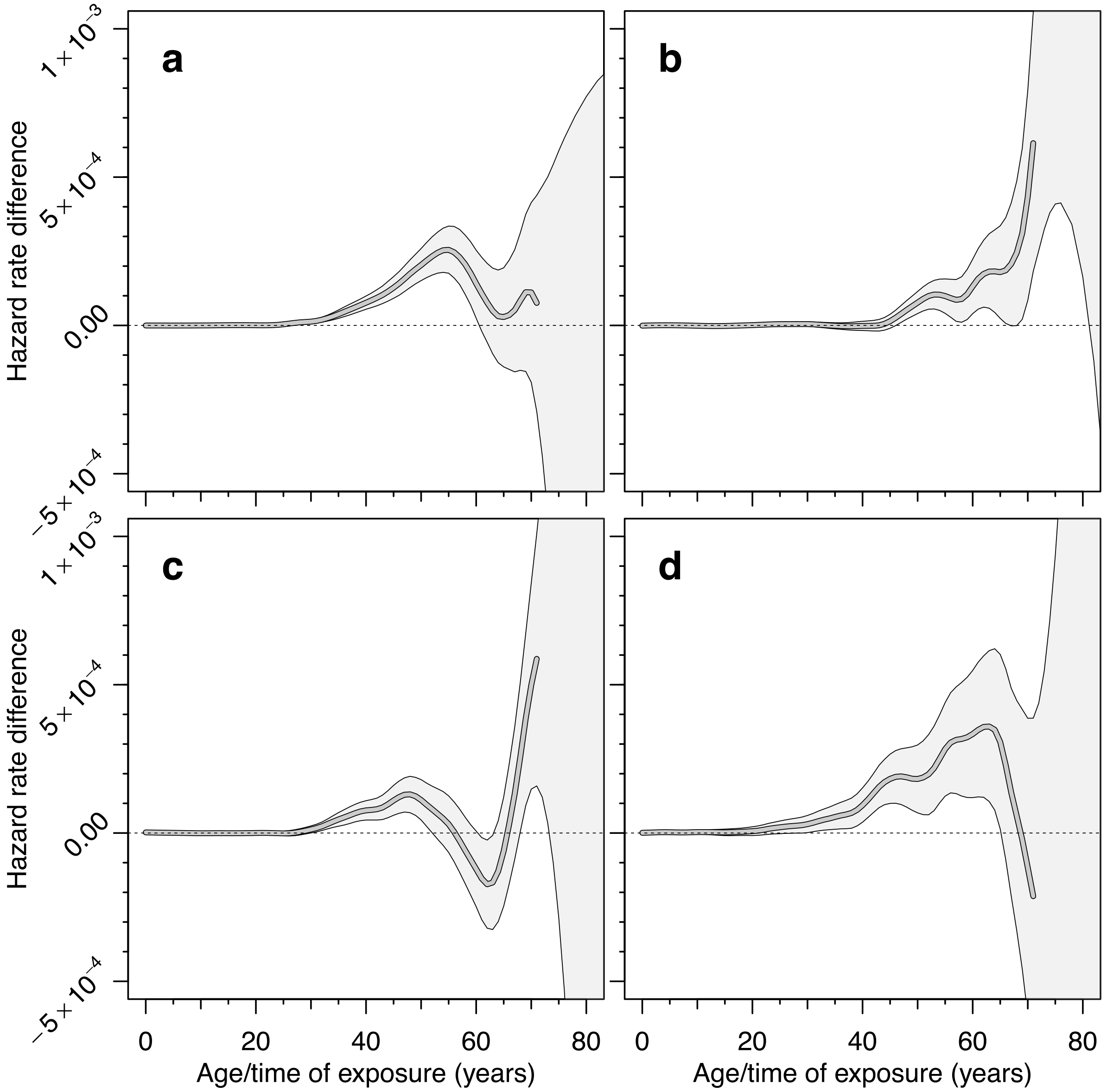

Figure S8. Trends of momentaneous effects in all individuals. Resolution of the trends is FDHM=10 years. High frequency random fluctuations below this resolution are suppressed while larger-scale temporal structures are retained. **a**) Estimated trend of the momentaneous effect on T2DM. **b**) Estimated trend of the momentaneous effect on atrial fibrillation. **c**) Estimated trend of the momentaneous effect on CAD. **d**) Estimated trend of the momentaneous effect on osteoarthritis. The plateau in the life-course effect of BMI on T2DM around 60 years of age (Fig. 4, main text) is preceded by a decrease in the momentaneous effect trend, towards the null. The trough in the cumulative effect for CAD shown in Fig. 4 (main text) is corroborated by a significant negative gradient for CAD in the age range 60-65 years. The momentaneous effect for atrial fibrillation shows a persistent increase with time, with no sign of a break below 70 years. On the other hand, the rise in the momentaneous effect for osteoarthritis appears not to continue above to ~65 years of age. T2DM = type 2 diabetes mellitus, CAD = coronary artery disease, FDHM = full duration at half maximum.

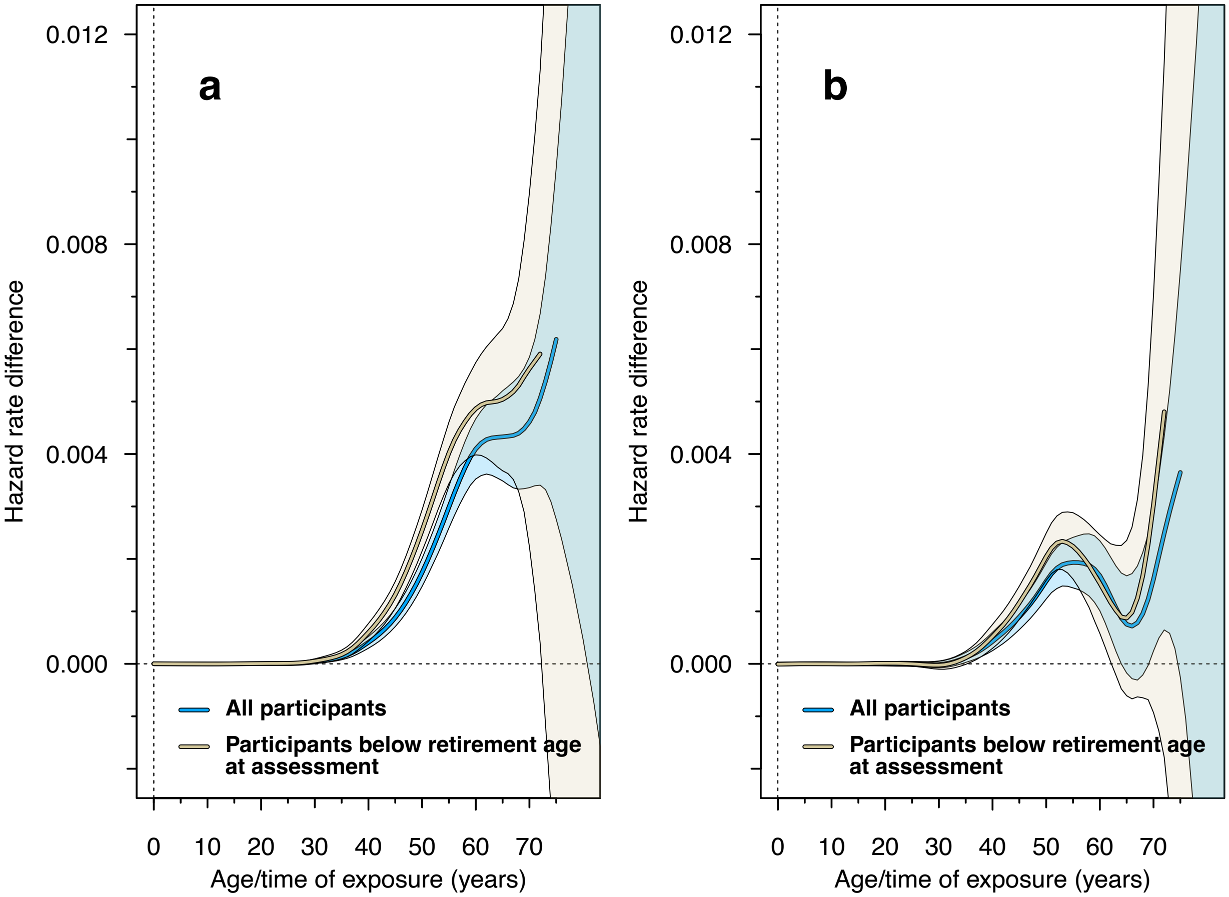

Figure S9. Time-series trends of cumulative effects of BMI on T2DM and CAD in participants who have not yet reached retirement age, at assessment. a) Trends of the effect on T2DM. Blue curve (with 95% CI) denotes the trend of the life-course effect for all participants, while the beige curve (with 95% CI) denotes the life-course effect for participants who had not yet reached retirement age, at assessment. **b**) Trends of the effect on CAD. Blue curve (with 95% CI) denotes the trend of the life-course effect for all participants, while the beige curve (with 95% CI) denotes the life-course effect for participants who had not yet reached retirement age, at assessment. These results suggest that potential changes in participation rates into UKB around retirement cannot fully explain the presence of these troughs and features. BMI = body mass index, T2DM = type 2 diabetes mellitus, CAD = coronary artery disease, CI = confidence interval.

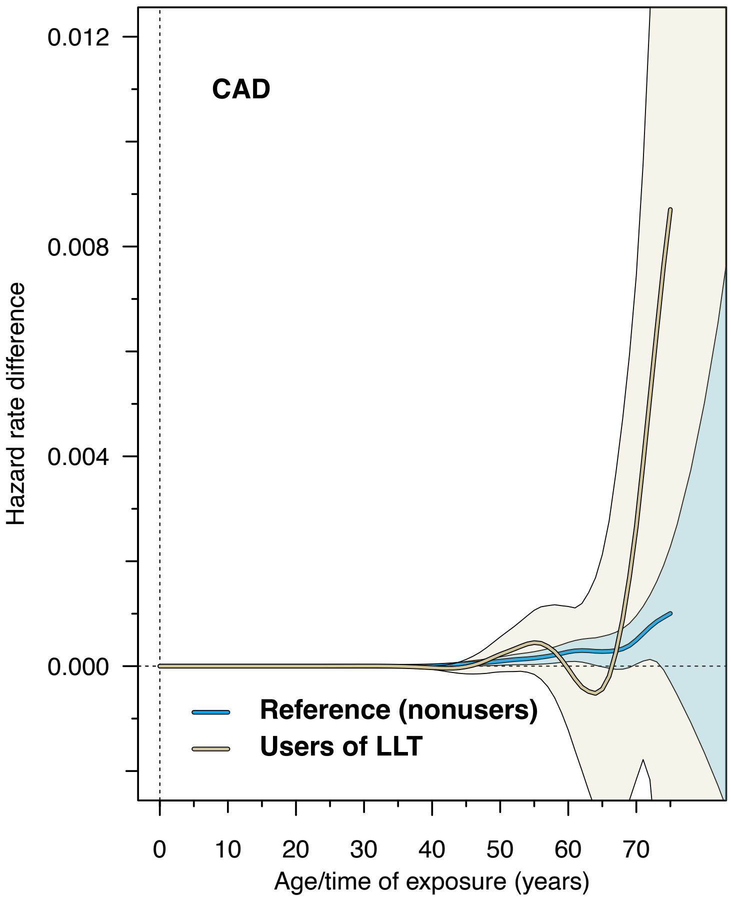

Figure S10. Time-series trends of cumulative effects of BMI on CAD in participants on lipid-lowering treatment, at assessment. Trend for participants regularly using lipid-lowering treatment (LLT) at assessment is denoted in beige. As reference, the trend for participants reporting that they do not take lipid-lowering treatment (LLT) at assessment is denoted in blue. The trends were estimated using Hodrick-Prescott filtering with $\lambda = 50$ (Materials and Methods). Parametric bootstrap with 10,000 bootstrap samples was used to calculate the 95% CIs of the trends. A clear trough in the trend of BMI on CAD is present for participants taking LLT, while the trend is largely absent for participants not taking LLT and antihypertensives. These results are broadly consistent with primary preventions strategies, temporarily reducing the total effect of BMI on CAD in the population. BMI = body mass index, CAD = coronary artery disease, LLT = lipid-lowering treatment, CI = confidence interval.

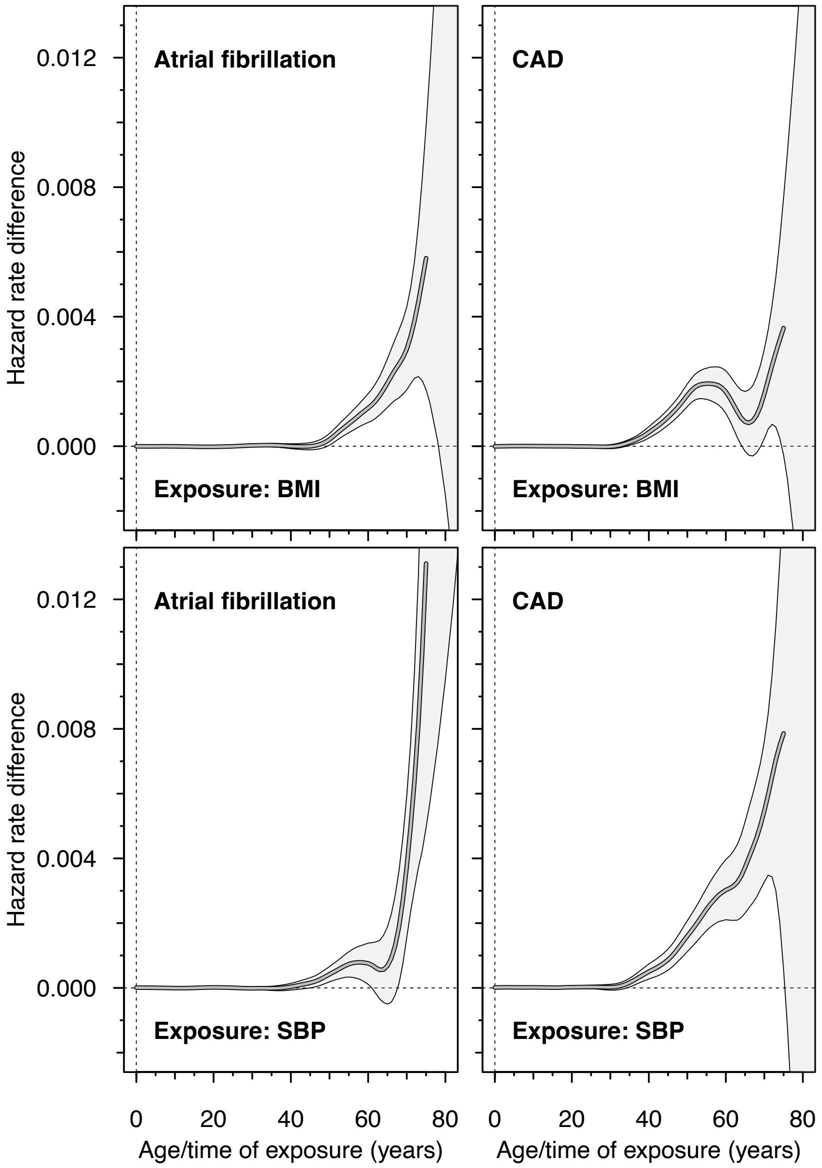

Figure S11. Time-series trends of cumulative effects of BMI and SBP on atrial fibrillation and CAD. The two top panels show trends of effects on atrial fibrillation and CAD with BMI as exposure (same as Fig. 4J and K, main text), while the bottom panels show trends of effects on atrial fibrillation and CAD with SBP as exposure. Trends were estimated using Hodrick-Prescott filtering with $\lambda = 50$ (Materials and Methods). Parametric bootstrap with 10,000 bootstrap samples was used to calculate the 95% CIs of the trends. A clear trough in the trend of BMI on CAD is present, while the effect of SBP on atrial fibrillation shows evidence of a moderate trough. No trough is detected in the trend of BMI on atrial fibrillation, and only a tendency of a trough is present in the trend of SBP on CAD. These results are broadly consistent with primary preventions strategies, temporarily reducing the total risk of disease in the population that is related to a specific exposure. BMI = body mass index, SBP = systolic blood pressure, CAD = coronary artery disease, CI = confidence interval.

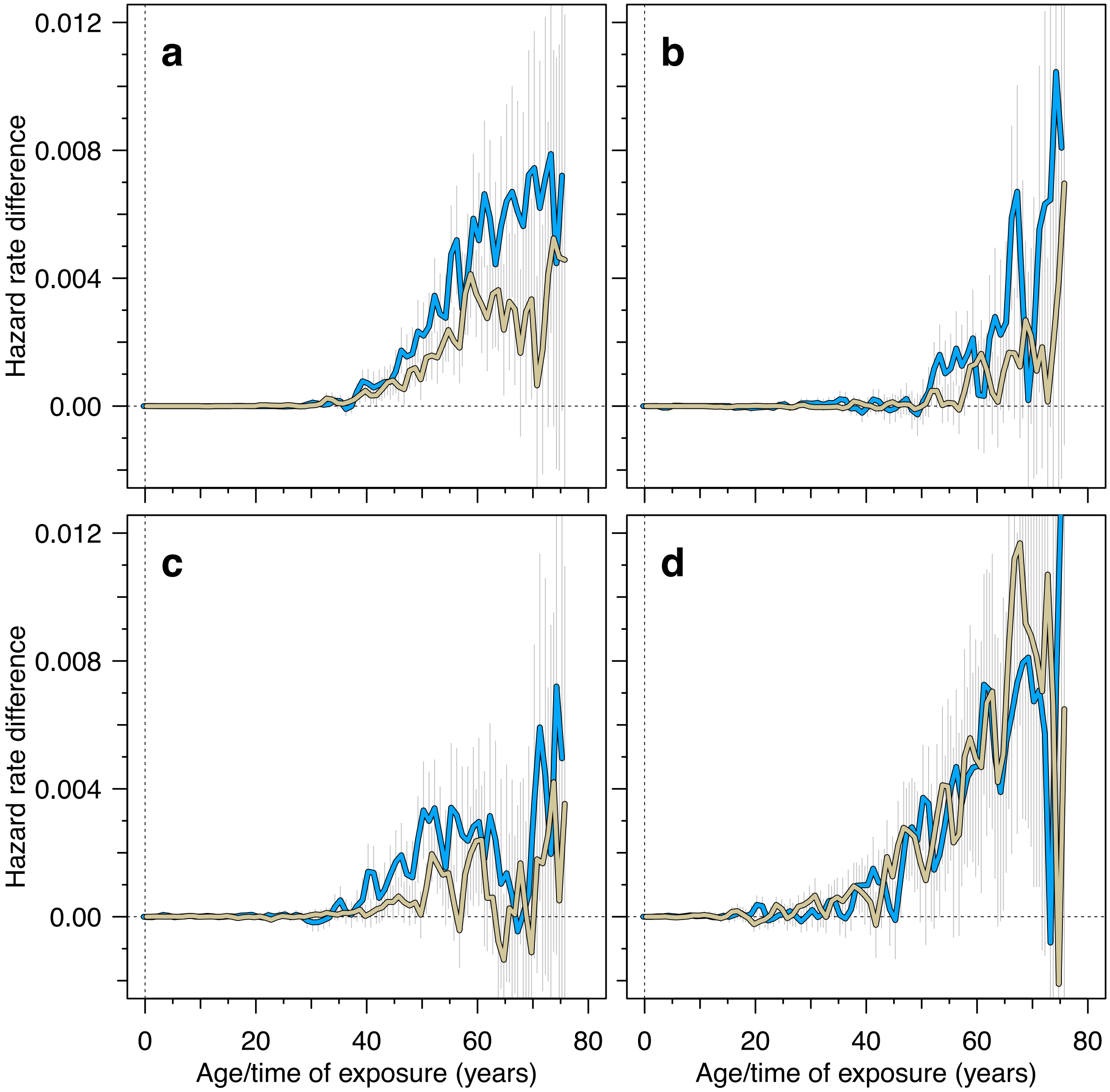

Figure S12. Time-resolved, cumulative effects of BMI on four complex diseases in females and males. Results for females are denoted in beige, while results for males are denoted in blue. Grey, vertical lines denote 95% CIs. The time-dependent difference in the hazard rate in females (number of cases at age $T \leq 76$ years $n_{\mathrm{F}} = 10{,}427$) and in males ($n_{\mathrm{M}} = 16{,}287$) for T2DM, having one standard deviation higher BMI during the life-course, up to a given age. The time-dependent differences in hazard rates for **b**) atrial fibrillation ($n_{\mathrm{F}} = 8{,}379$; $n_{\mathrm{M}} = 15{,}565$), **c**) CAD ($n_{\mathrm{F}} = 13{,}807$; $n_{\mathrm{M}} = 27{,}399$), and **d**) osteoarthritis ($n_{\mathrm{F}} = 52{,}329$; $n_{\mathrm{M}} = 36{,}241$). Cumulative effects are numerically integrated over time using the trapezoidal rule (Materials and Methods). Corresponding time-series trends of the cumulative effects are presented in Fig. 5 (main text). BMI = body mass index, T2DM = type 2 diabetes mellitus, CAD = coronary artery disease, CI = confidence interval.

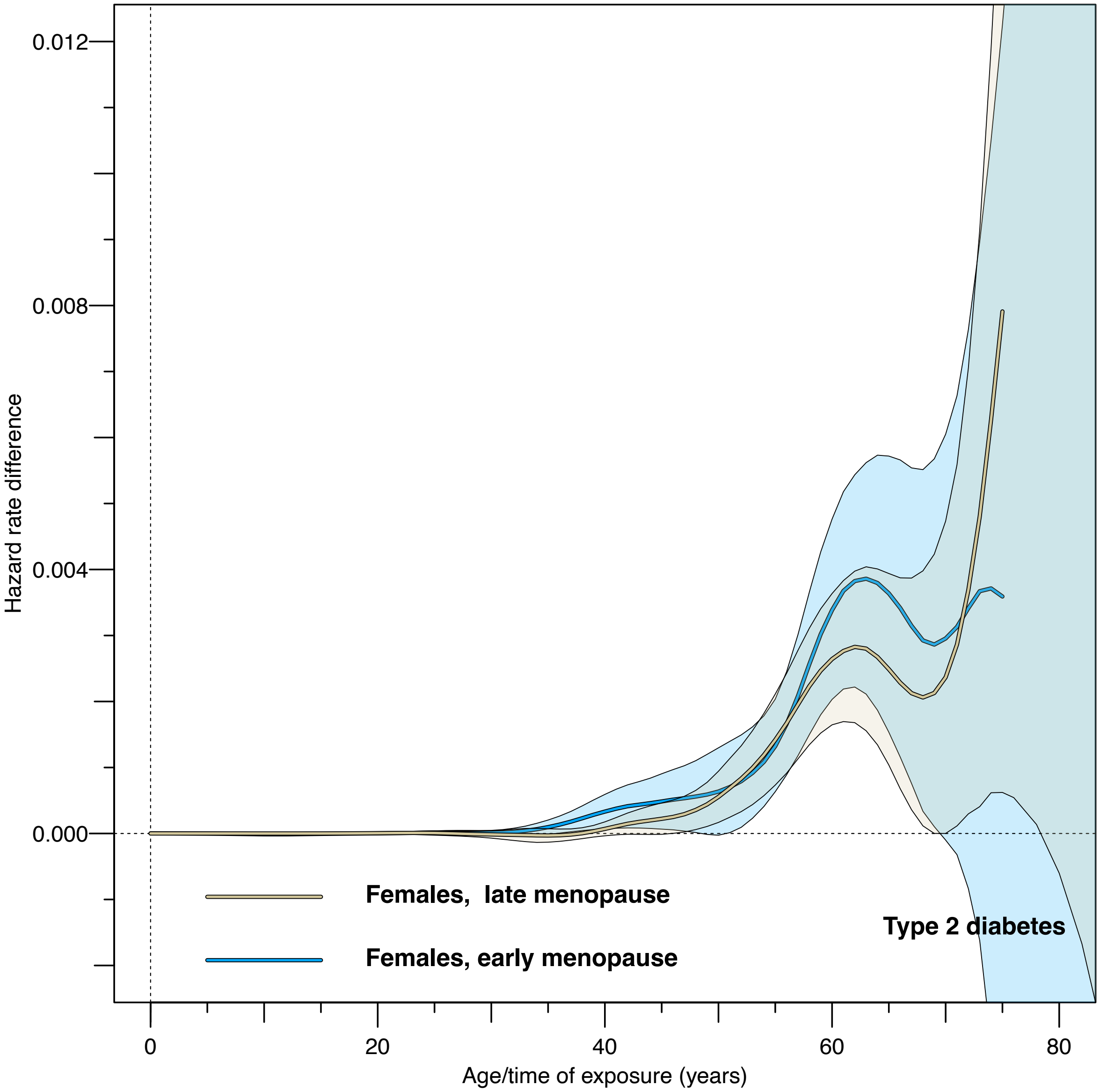

Figure S13. Time-series trends of effects of sustained BMI on incident type 2 diabetes in women with early and late menopause. Time-series trend of the effect of BMI on T2DM in women with early menopause is denoted in blue, while the trend of the effect in women with late menopause is denoted in beige. There is a difference in mean age at menopause of 9 years between the "late menopause" and "early menopause" samples. Trends were estimated from raw cumulative effects using Hodrick-Prescott filtering with $\lambda = 50$ (Materials and Methods). Shaded areas denote corresponding 95% CIs, which were calculated using parametric bootstrap with 10,000 bootstrap samples. The timing of the trough appears not to depend on the timing of menopause. However, women with early menopause tend to show a slightly higher risk of diabetes at > 55 years of age. BMI = Body Mass Index, T2DM = type 2 diabetes mellitus, CI = confidence interval.

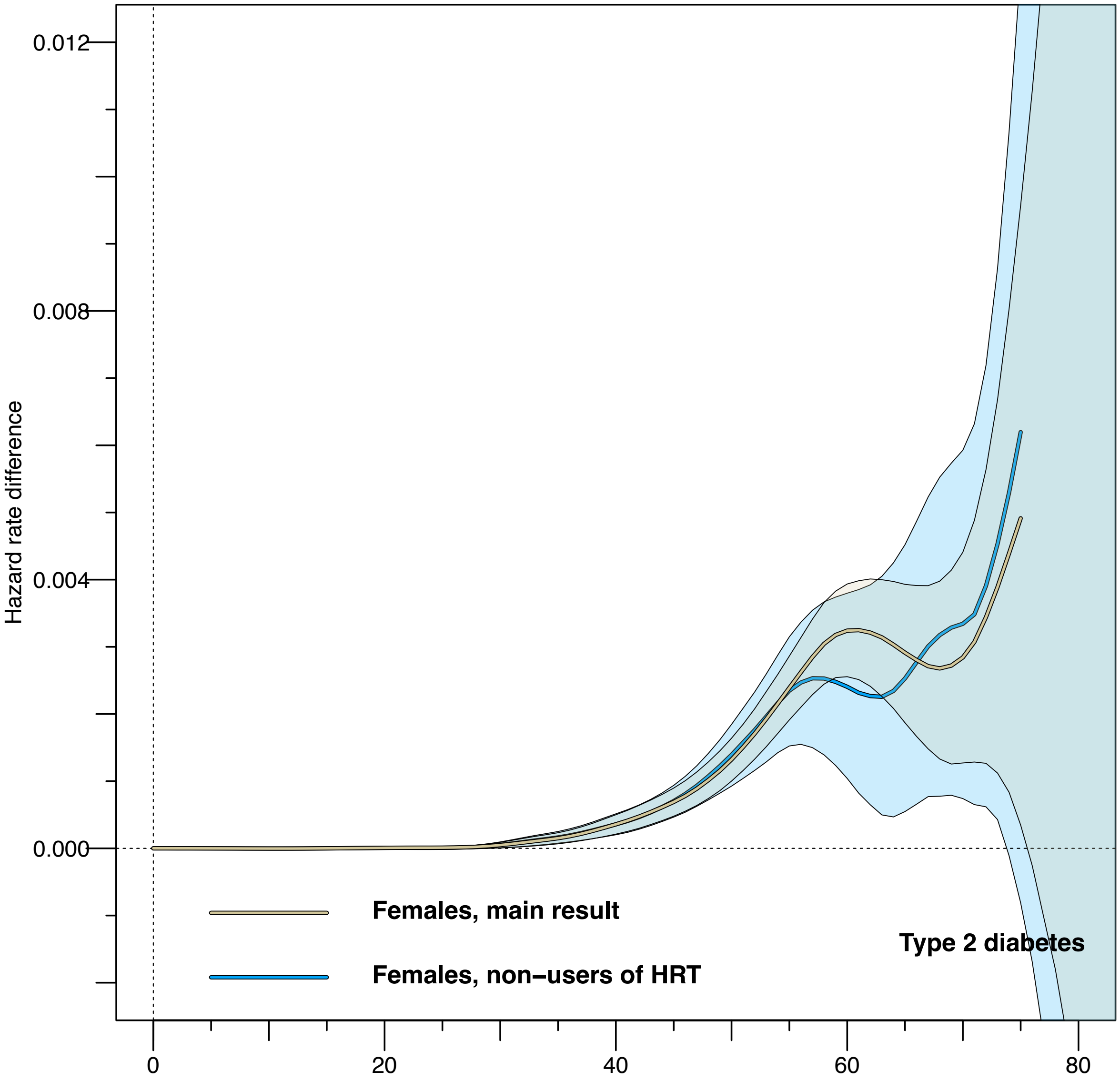

Figure S14. Time-series trend of effect of sustained BMI on incident type 2 diabetes in non-users of menopausal hormone therapy. Time-series trend of the effect of BMI on T2DM in non-users of HRT is denoted in blue, while the trend of the effect in all females (same as Fig. 5A, main text) is denoted in beige. Trends were estimated from raw cumulative effects using Hodrick-Prescott filtering with $\lambda = 50$ (Materials and Methods). Shaded areas denote corresponding 95% CIs, which were calculated using parametric bootstrap with 10,000 bootstrap samples. Also in non-users, a temporary decline in effect is present, which suggests that menopausal hormone therapy medication cannot be entirely responsible for the occurrence of the trough. BMI = Body Mass Index, T2DM = type 2 diabetes mellitus, HRT = Hormone replacement therapy (= menopausal hormone therapy, here), CI = confidence interval.

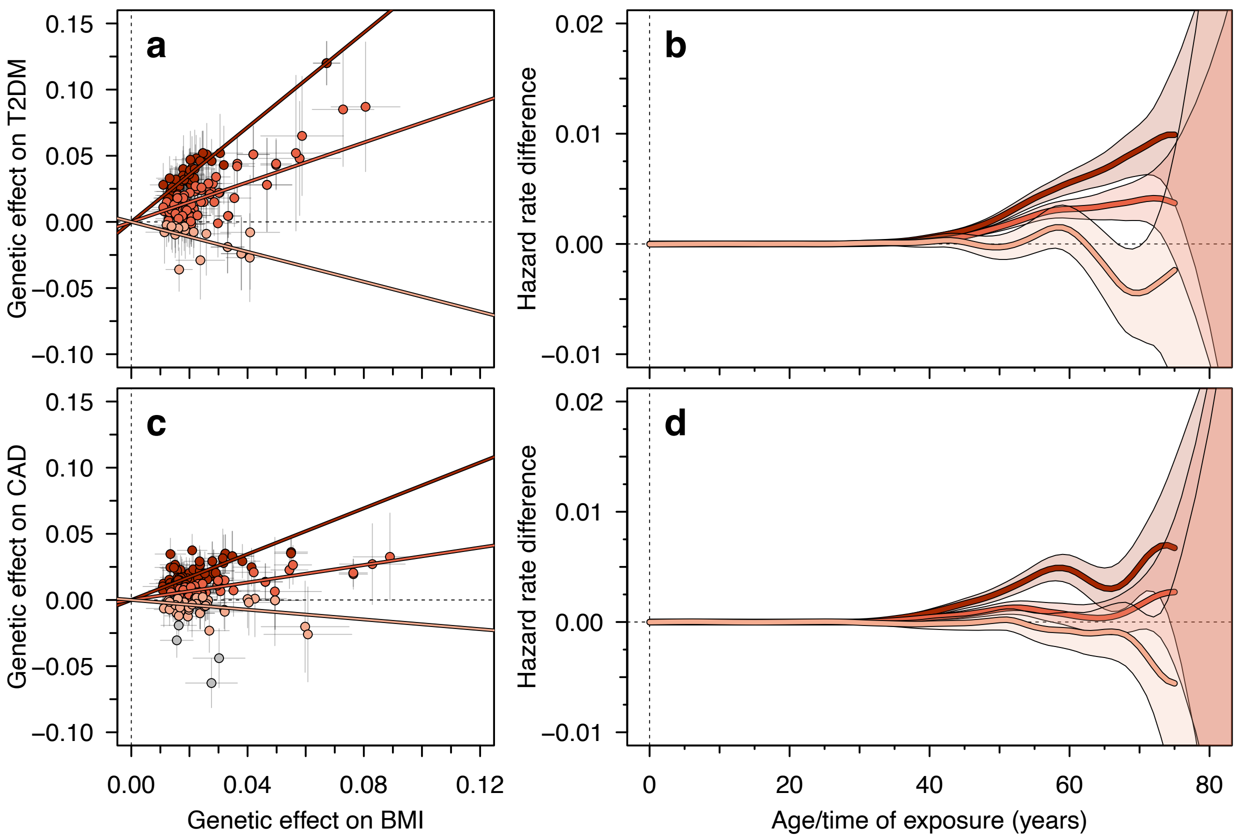

Figure S15. Independent causal pathways between BMI and the cardiometabolic diseases T2DM and CAD. Clustering of BMI-associated SNPs, related to the onset of disease through potentially independent mechanisms. **a**) Partitioning of BMI-associated SNPs based on the SNPs' corresponding associations (log[OR]) with T2DM using agglomerate hierarchical clustering (*30*). Three clusters were identified, corresponding to three potentially independent causal pathways. Dark, ruby red dots denote SNPs belonging to the high-risk pathway, while medium red dots denote SNPs belonging to the medium-risk pathway. SNPs belonging to the protective pathway are displayed in light rose red. This color-coding was adopted in all panels throughout the figure. The solid lines indicate corresponding effects, estimated from standard MR analysis. **b**) Trends of time-resolved, cumulative effects of BMI on T2DM using three independent PGSs, where each score was generated from SNPs belonging to a specific cluster. Parametric bootstrap with 10,000 bootstrap samples was used to calculate the 95% CIs for the time-series trends. The protective pathway is significantly negative at around 70 years of age (*cf*. Fig. 5A). **c**) Partitioning of BMI-associated SNPs, based on the SNPs' corresponding associations (log[OR]) with CAD, into three independent clusters. The four dots displayed in grey were identified as outliers by the clustering algorithm and were removed from further analysis. **d**) Trends of time-resolved, cumulative effects of BMI on CAD corresponding to the three clusters for CAD, identified by the clustering algorithm. The high-risk pathway shows a clear trough in the trend at around 65 years of age (*cf*. Fig. 5C). Although not significant, the protective pathway shows a consistently negative effect from about 50 years of age, and onwards. BMI = body mass index, T2DM = type 2 diabetes mellitus, CAD = coronary artery disease, SNP = single nucleotide polymorphism, PGS = polygenic score, CI = confidence interval.

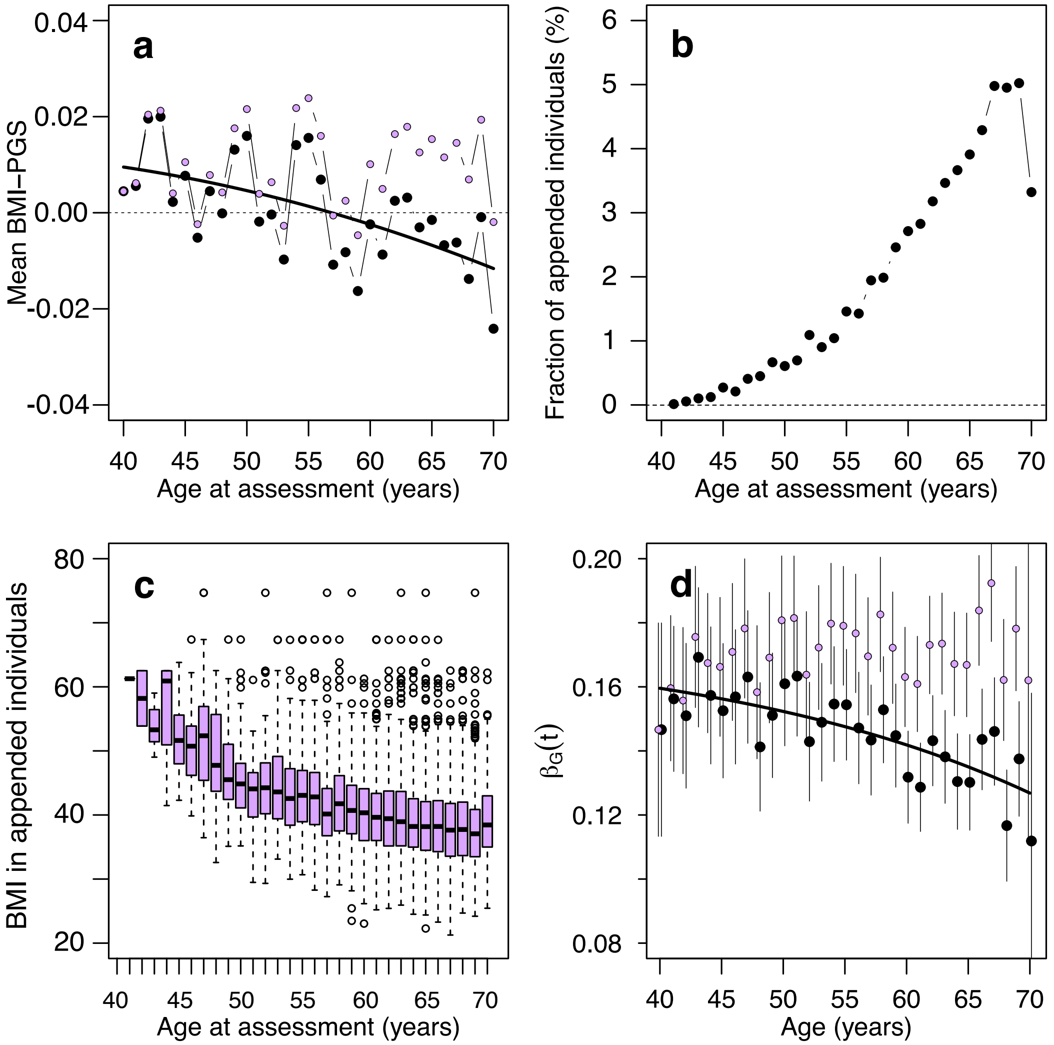

Figure S16. Descriptive statistics for relative correction of selection into UK Biobank. a) Trend in mean BMI-PGS. Black dots denote age stratified mean BMI-PGS for the original UKB data set while the purple dots denote age stratified mean BMI-PGS for the expanded UKB data set, including appended individuals with high BMI. The black curve denotes the expected mean BMI-PGS $\mathbb{E}\langle \text{BMI-PGS} \rangle_{k,\text{orig}} = \mathbb{E}\langle \text{BMI-PGS} \rangle_{40,\text{orig}} - m_k$, where $\mathbb{E}\langle \text{BMI-PGS} \rangle_{40,\text{orig}}$ denotes the expected mean BMI-PGS at 40 years of age and m_k denotes the relative expected offset (see Materials and Methods). **b**) Fraction of appended individuals per age stratum. Overall fraction of appended individuals was 2.2%. **c**) Boxplots indicating the distributions of BMI for the appended individuals per age stratum. **d**) Time-dependent effect of BMI-PGS on BMI for the original UKB data set (black dots and black curve) and for the expanded UKB data set, including appended individuals with high BMI (purple dots). BMI = Body Mass Index, PGS = polygenic score, UKB = UK Biobank.

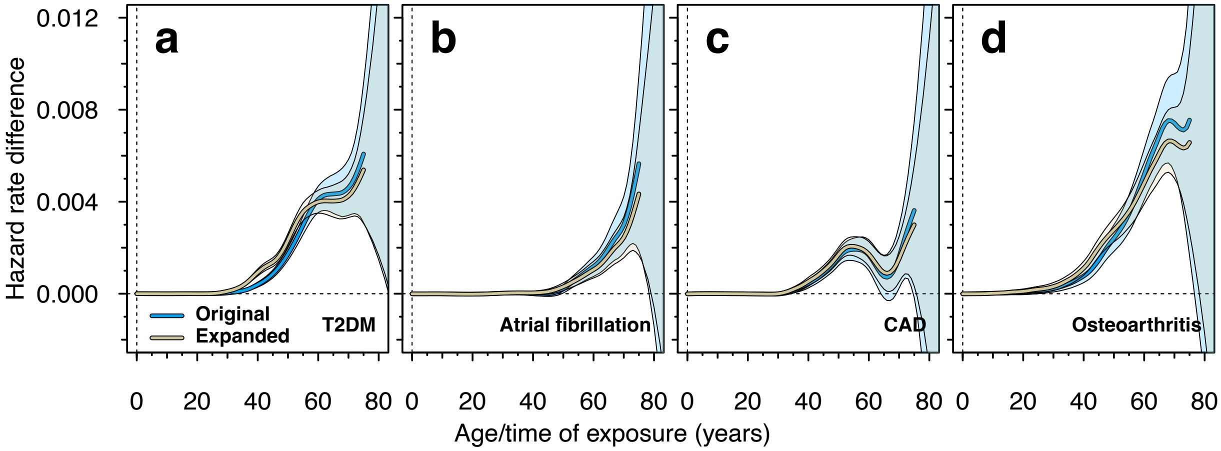

Figure S17. Trends of cumulative effects corrected for selection into UK Biobank. Time-series trends of life-course effects estimated using Hodrick-Prescott filtering with $\lambda = 50$, are presented for **a**) T2DM, **b**) AF, **c**) CAD, and **d**) OA. Original, main results (Fig. 4, main text) are denoted in blue, while results using the expanded UKB data set corrected for selection (Materials and Methods) are denoted in beige. Parametric bootstrap with 10,000 bootstrap samples was used to calculate the 95% CIs of the trends. Minor systematic differences are observed. However, all previously identified features and troughs are still present. BMI = body mass index, T2DM = type 2 diabetes mellitus, AF = atrial fibrillation, CAD = coronary artery disease, OA = osteoarthritis, CI = confidence interval.

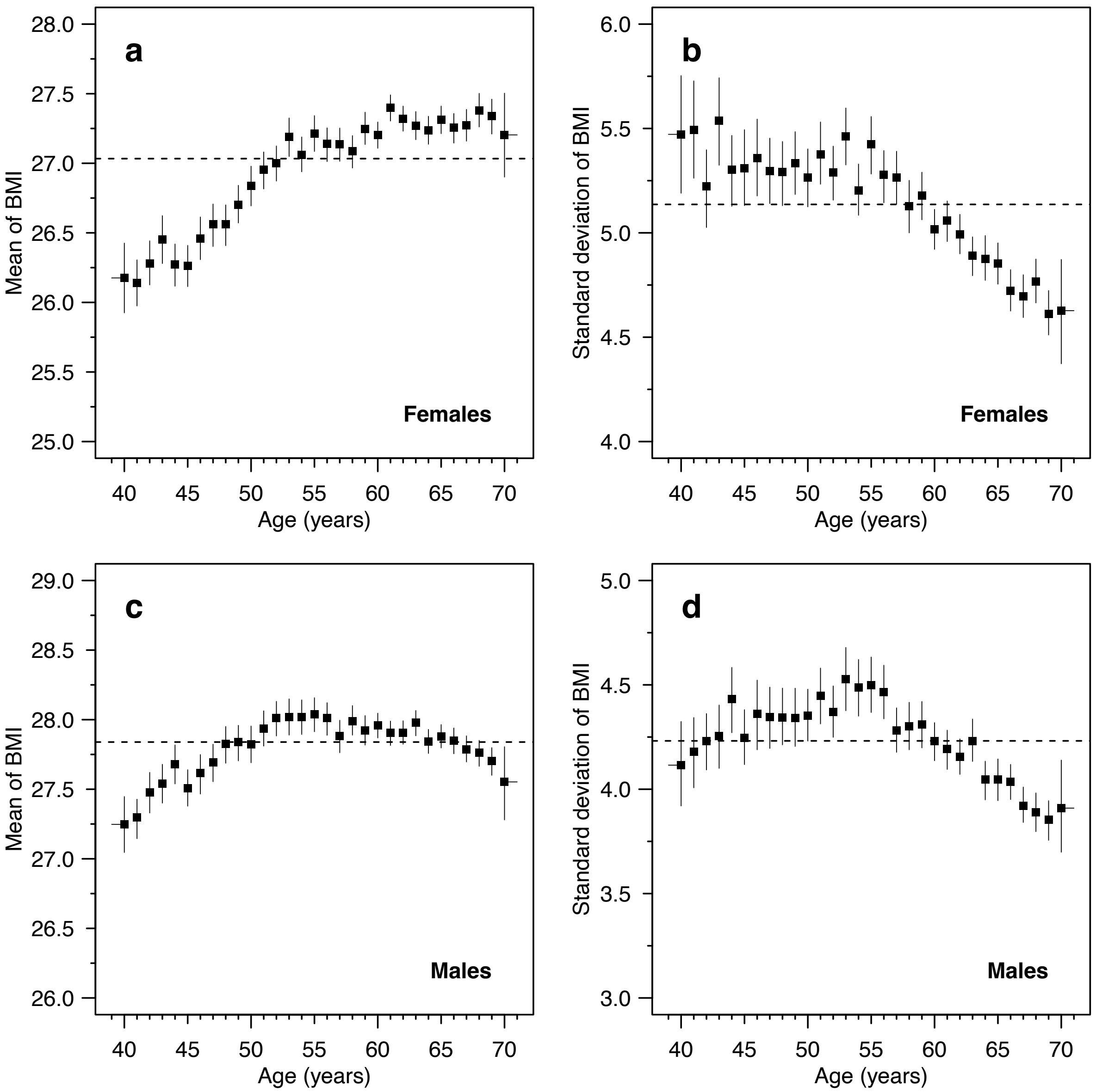

Figure S18. Mean and standard deviation of BMI for both sex and age strata. a) Mean BMI in UKB females for different age strata. **b**) Standard deviation of BMI in UKB females for different age strata. **c**) Mean BMI in UKB males for different age strata. **d**) Standard deviation of BMI in UKB males for different age strata. Vertical lines denote 95% basic bootstrap CI, using 1,000 bootstrap samples. The mean and standard deviation of the lowest and highest age strata are calculated using all individuals with age $\leq$ 40 years and age $\geq$ 70 years, respectively, and is indicated by the horizontal line attached to the corresponding square symbol. The dashed lines denote the marginal mean and standard deviation, taken over all ages. Note that, while females have a lower mean BMI as compared to males, the standard deviation of BMI is higher in females. BMI = body mass index, UKB = UK Biobank, CI = confidence interval.

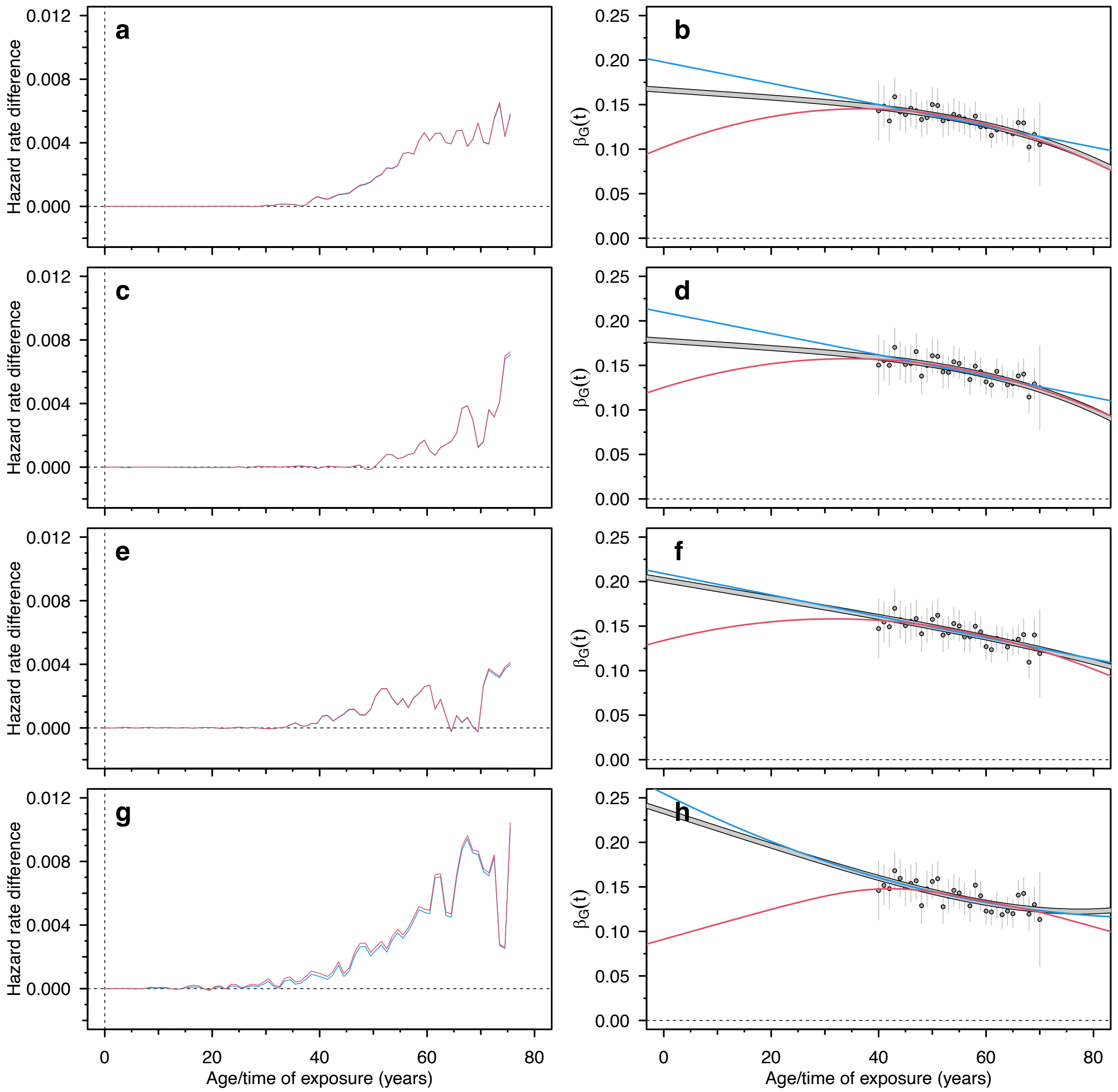

Figure S19. Sensitivity to systematic uncertainty in the estimation of $\boldsymbol{\beta_G(t)}$ at young ages. a) Life-course effects on T2DM estimated in all individuals for an extrapolated instrument effect on BMI that is larger (blue) and smaller (red) at young age ($t < 40$ years), compared with the adopted $\widehat{\beta_G}(t)$. **b**) Corresponding $\widehat{\beta_G}(t)$ for the estimated life-course effects on T2DM. The thick, grey curve denotes the time-dependent instrument effect on BMI for T2DM that was adopted in the main analysis. Grey dots denote age-stratified, main effect estimates on BMI. **c**) Life-course effects on AF, estimated in all individuals for a larger (blue) and smaller (red) $\widehat{\beta_G}(t)$ at young age. **d**) Corresponding $\widehat{\beta_G}(t)$ for AF. **e**) Life-course effects on CAD, estimated in all individuals for a larger (blue) and smaller (red) $\widehat{\beta_G}(t)$ at young age. **f**) Corresponding $\widehat{\beta_G}(t)$ for CAD. **g**) Life-course effects on OA, estimated in all individuals for a larger (blue) and smaller (red) $\widehat{\beta_G}(t)$ at young age. **h**) Corresponding $\widehat{\beta_G}(t)$ for OA. Due to negligible effect accumulations at young age, the life-course effects are found to be insensitive to uncertainties in the estimations of $\beta_G(t)$ for all diseases investigated in this study. Curves and symbols in all panels are color coded in the same way. BMI = body mass index, T2DM = type 2 diabetes mellitus, AF = atrial fibrillation, CAD = coronary artery disease, OA = osteoarthritis.

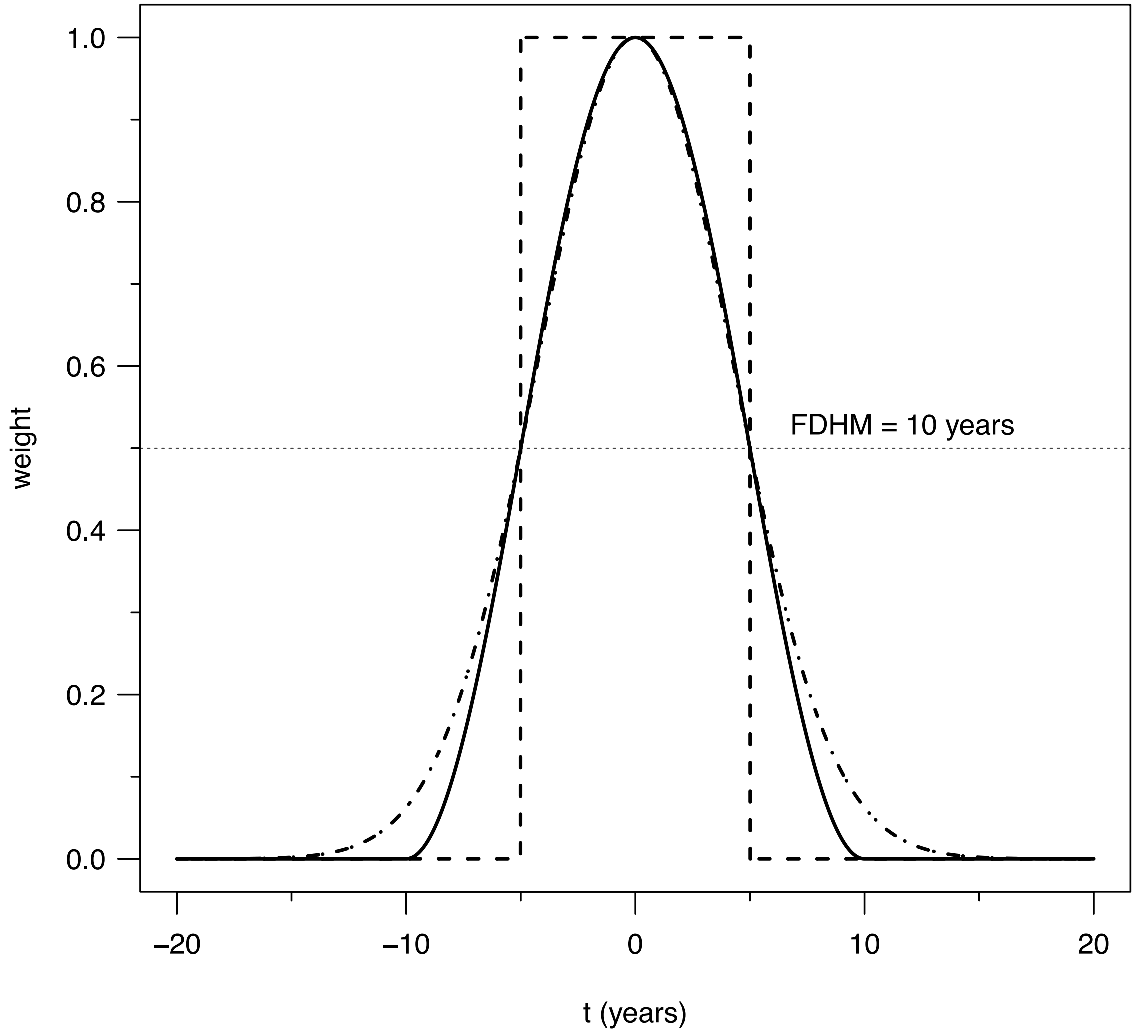

Figure S20. Comparison of different weight functions. The dashed curve denotes a rectangular, or box, kernel, the dash-dotted curve denotes a gaussian kernel, while the solid curve denotes the full-period cosine kernel used for estimation of the trend of the momentaneous effect in this study. All depicted kernels have a FDHM of 10 years. FDHM = Full Duration at Half Maximum.

Supplementary Tables

Table S1. Summary of parameters in data-generating model for the main simulation

	Effect parameter	Functional form
**Exposure $\boldsymbol{x}(\boldsymbol{t})$**	$\beta_0(t)$	12
	$\beta_G(t)$	$0.3 - 9 \times 10^{-4}t - 4 \times 10^{-9}t^4$
	$\beta_u(t)$	$0.1 + 4 \times 10^{-3}t - 1 \times 10^{-4}t^2$
	$\beta_z(t)$	$6 \times 10^{-2}t - 6 \times 10^{-4}t^2$
**Latent outcome $\boldsymbol{v}(\boldsymbol{t})$***	$\gamma_0(t)$	$-60c_x t^4$
	$\gamma_u(t)$	$2.5 \times 10^5 c_x t - 3 \times 10^3 c_x t^2 + 5 \times 10^{-2} c_x t^5$
	$\gamma_z(t)$	$-3 \times 10^{-1} c_x t^5 + 3 \times 10^{-3} c_x t^6$
	$\gamma(t)$	$5c_x t^4$
**Observed outcome $\boldsymbol{\Upsilon}(\boldsymbol{T})$***	C_0	1×10^{-8}
	$\Gamma_0(T)$	$-60c_x T^5/5$
	$\Gamma_u(T)$	$2.5 \times 10^5 c_x T^2/2 - 3 \times 10^3 c_x T^3/3 + 5 \times 10^{-2} c_x T^6/6$
	$\Gamma_z(T)$	$-3 \times 10^{-1} c_x T^6/6 + 3 \times 10^{-3} c_x T^7/7$
**Life-course effect of $\boldsymbol{x}$***	$\Gamma(T)$	$c_x T^5$

*The normalization factor $c_x = 0.01/75^5$ normalizes the cumulative life-course effect of exposure on outcome to be 0.01 yr^{-1} at 75 years of age.
The sought-after effects of exposure on observed and latent outcomes are highlighted in beige.

Table S2. Definitions of outcome diseases, exposure and covariate variables

Disease/covariate	Disease code[‡‡] (ICD-10)	Data type	Data unit	UKB data-field	Source/category
**Type 2 diabetes mellitus**	E11	continuous	date	130708	Endocrine, nutritional and metabolic diseases
**Atrial fibrillation**	I48	continuous	date	131350	Circulatory system disorders
**Coronary artery disease**	I20, I21, I22, I23, I24, I25	continuous	date	131296, 131298, 131300, 131302, 131304, 131306	Circulatory system disorders
**Osteoarthritis**	M15, M16, M17, M18, M19	continuous	date	131868, 131870, 131872, 131874, 131876	Musculoskeletal system and connective tissue disorders
**Body mass index (BMI)**[*]		continuous	kg/m^2	21001	Baseline characteristics
**Sex**[†]		binary	-	31	Baseline characteristics
**Year of birth**[‡]		continuous	years	34	Baseline characteristics
**Age at assessment**[††]		continuous	years	21003	Baseline characteristics
**Year of death**[‡]		continuous	years	40000	Death register
**Assessment centre**[†]		categorical	-	54	Reception
**Genotyping array**[†]		binary	-	22000	Genotyping process and sample QC
**Genetic PCs**[†]		continuous	-	22009	Genotyping process and sample QC

[*]Principal exposure in all analyses.
[†]Covariate in the main models, sex is excluded in the sex-stratified models, PC=principal component.
[‡]Variable used to calculate age at end of follow-up, as given by the expression min(2020 – year of birth, year of death – year of birth). Year of birth was also used to calculate age at diagnosis as given by year of first occurrence of disease – year of birth.
[††]Age at assessment was included as covariate in the GWAS model, age was also used as primary timescale in Aalen's hazard model.
[‡‡]First occurrence of disease codes from primary care data, hospital inpatient data, death register records, and self-reported medical condition codes are mapped to 3-character ICD-10 codes in UKB.

Table S3. Information on GWAS analyses of the cardiometabolic outcome diseases

	**Type 2 diabetes**	**Coronary artery disease**
**Publication**	Mahajan *et al.* (2018)	van der Harst &Verweij (2018)
**GWAS analysis**	Meta	Meta
**Outcome population(s)**	European/Caucasian	European
**Cases/controls**	55,005/400,308	122,733/424,528
**Overlap with UKB[*] (%)**	0.0	28.1

[*]Overlap was measured in terms of the fraction of all cases in the GWAS meta-analysis that originated from the UK Biobank.

Data Legends

Legend for Data S1 (separate file). Summary statistics of the 122+106 genome-wide significant lead SNPs identified in the two UKB subsamples. The file also includes information on disease-specific Steiger filtering removal and cluster membership for each SNP.

REFERENCES AND NOTES

1. GBD 2019 Diseases and Injuries Collaborators, Global burden of 369 diseases and injuries in 204 countries and territories, 1990-2019: A systematic analysis for the Global Burden of Disease Study 2019. *Lancet* **396**, 1204–1222 (2020).

2. NCD Risk Factor Collaboration (NCD-RisC), Worldwide trends in underweight and obesity from 1990 to 2022: A pooled analysis of 3663 population-representative studies with 222 million children, adolescents, and adults. *Lancet* **403**, 1027–1050 (2024).

3. M. Rask-Andersen, T. Karlsson, W. E. Ek, Å. Johansson, Genome-wide association study of body fat distribution identifies adiposity loci and sex-specific genetic effects. *Nat. Commun.* **10**, 339 (2019).

4. T. Karlsson, M. Rask-Andersen, G. Pan, J. Höglund, C. Wadelius, W. E. Ek, Å. Johansson, Contribution of genetics to visceral adiposity and its relation to cardiovascular and metabolic disease. *Nat. Med.* **25**, 1390–1395 (2019).

5. M. Rask-Andersen, E. Ivansson, J. Höglund, W. E. Ek, T. Karlsson, Å. Johansson, Adiposity and sex-specific cancer risk. *Cancer Cell* **41**, 1186–1197.e4 (2023).

6. V. Patwardhan, G. F. Gil, A. Arrieta, J. Cagney, E. DeGraw, M. E. Herbert, M. Khalil, E. C. Mullany, E. M. O'Connell, C. N. Spencer, C. Stein, A. Valikhanova, E. Gakidou, L. S. Flor, Differences across the lifespan between females and males in the top 20 causes of disease burden globally: A systematic analysis of the Global Burden of Disease Study 2021. *Lancet Public Health* **9**, e282–e294 (2024).

7. E. Sanderson, M. M. Glymour, M. V. Holmes, H. Kang, J. Morrison, M. R. Munafò, T. Palmer, C. M. Schooling, C. Wallace, Q. Zhao, G. Davey Smith, Mendelian randomization. *Nat Rev Methods Primers* **2**, 6 (2022).

8. S. Burgess, D. S. Small, S. G. Thompson, A review of instrumental variable estimators for Mendelian randomization. *Stat. Methods Med. Res.* **26**, 2333–2355 (2017).

9. J. MacArthur, E. Bowler, M. Cerezo, L. Gil, P. Hall, E. Hastings, H. Junkins, A. McMahon, A. Milano, J. Morales, Z. M. Pendlington, D. Welter, T. Burdett, L. Hindorff, P. Flicek, F. Cunningham, H. Parkinson, The new NHGRI-EBI catalog of published genome-wide association studies (GWAS Catalog). *Nucleic Acids Res.* **45**, D896–D901 (2017).

10. G. Hemani, J. Zheng, B. Elsworth, K. H. Wade, V. Haberland, D. Baird, C. Laurin, S. Burgess, J. Bowden, R. Langdon, V. Y. Tan, J. Yarmolinsky, H. A. Shihab, N. J. Timpson, D. M. Evans, C. Relton, R. M. Martin, G. Davey Smith, T. R. Gaunt, P. C. Haycock, The MR-Base platform supports systematic causal inference across the human phenome. *eLife* **7**, e34408 (2018).

11. J. A. Labrecque, S. A. Swanson, Interpretation and potential biases of Mendelian randomization estimates with time-varying exposures. *Am. J. Epidemiol.* **188**, 231–238 (2019).

12. T. W. Winkler, A. E. Justice, M. Graff, L. Barata, M. F. Feitosa, S. Chu, J. Czajkowski, T. Esko, T. Fall, T. O. Kilpeläinen, Y. Lu, R. Mägi, E. Mihailov, T. H. Pers, S. Rüeger, A. Teumer, G. B. Ehret, T. Ferreira, N. L. Heard-Costa, J. Karjalainen, V. Lagou, A. Mahajan, M. D. Neinast, I. Prokopenko, J. Simino, T. M. Teslovich, R. Jansen, H.-J. Westra, C. C. White, D. Absher, T. S. Ahluwalia, S. Ahmad, E. Albrecht, A. C. Alves, J. L. Bragg-Gresham, A. J. M. De Craen, J. C. Bis, A. Bonnefond, G. Boucher, G. Cadby, Y.-C. Cheng, C. W. K. Chiang, G. Delgado, A. Demirkan, N. Dueker, N. Eklund, G. Eiriksdottir, J. Eriksson, B. Feenstra, K. Fischer, F. Frau, T. E. Galesloot, F. Geller, A. Goel, M. Gorski, T. B. Grammer, S. Gustafsson, S. Haitjema, J.-J. Hottenga, J. E. Huffman, A. U. Jackson, K. B. Jacobs, Å. Johansson, M. Kaakinen, M. E. Kleber, J. Lahti, I. M. Leach, B. Lehne, Y. Liu, K. S. Lo, M. Lorentzon, J. Luan, P. A. F. Madden, M. Mangino, B. McKnight, C. Medina-Gomez, K. L. Monda, M. E. Montasser, G. Müller, M. Müller-Nurasyid, I. M. Nolte, K. Panoutsopoulou, L. Pascoe, L. Paternoster, N. W. Rayner, F. Renström, F. Rizzi, L. M. Rose, K. A. Ryan, P. Salo, S. Sanna, H. Scharnagl, J. Shi, A. V. Smith, L. Southam, A. Stančáková, V. Steinthorsdottir, R. J. Strawbridge, Y. J. Sung, I. Tachmazidou, T. Tanaka, G. Thorleifsson, S. Trompet, N. Pervjakova, J. P. Tyrer, L. Vandenput, S. W. Van Der Laan, N. Van Der Velde, J. Van Setten, J. V. Van Vliet-Ostaptchouk, N. Verweij, E. Vlachopoulou, L. L. Waite, S. R. Wang, Z. Wang, S. H. Wild, C. Willenborg, J. F. Wilson, A. Wong, J. Yang, L. Yengo, L. M. Yerges-

Armstrong, L. Yu, W. Zhang, J. H. Zhao, E. A. Andersson, S. J. L. Bakker, D. Baldassarre, K. Banasik, M. Barcella, C. Barlassina, C. Bellis, P. Benaglio, J. Blangero, M. Blüher, F. Bonnet, L. L. Bonnycastle, H. A. Boyd, M. Bruinenberg, A. S. Buchman, H. Campbell, Y.-D. I. Chen, P. S. Chines, S. Claudi-Boehm, J. Cole, F. S. Collins, E. J. C. De Geus, L. C. P. G. M. De Groot, M. Dimitriou, J. Duan, S. Enroth, E. Eury, A.-E. Farmaki, N. G. Forouhi, N. Friedrich, P. V. Gejman, B. Gigante, N. Glorioso, A. S. Go, O. Gottesman, J. Gräßler, H. Grallert, N. Grarup, Y.-M. Gu, L. Broer, A. C. Ham, T. Hansen, T. B. Harris, C. A. Hartman, M. Hassinen, N. Hastie, A. T. Hattersley, A. C. Heath, A. K. Henders, D. Hernandez, H. Hillege, O. Holmen, K. G. Hovingh, J. Hui, L. L. Husemoen, N. Hutri-Kähönen, P. G. Hysi, T. Illig, P. L. De Jager, S. Jalilzadeh, T. Jørgensen, J. W. Jukema, M. Juonala, S. Kanoni, M. Karaleftheri, K. T. Khaw, L. Kinnunen, S. J. Kittner, W. Koenig, I. Kolcic, P. Kovacs, N. T. Krarup, W. Kratzer, J. Krüger, D. Kuh, M. Kumari, T. Kyriakou, C. Langenberg, L. Lannfelt, C. Lanzani, V. Lotay, L. J. Launer, K. Leander, J. Lindström, A. Linneberg, Y.-P. Liu, S. Lobbens, R. Luben, V. Lyssenko, S. Männistö, P. K. Magnusson, W. L. McArdle, C. Menni, S. Merger, L. Milani, G. W. Montgomery, A. P. Morris, N. Narisu, M. Nelis, K. K. Ong, A. Palotie, L. Pérusse, I. Pichler, M. G. Pilia, A. Pouta, M. Rheinberger, R. Ribel-Madsen, M. Richards, K. M. Rice, T. K. Rice, C. Rivolta, V. Salomaa, A. R. Sanders, M. A. Sarzynski, S. Scholtens, R. A. Scott, W. R. Scott, S. Sebert, S. Sengupta, B. Sennblad, T. Seufferlein, A. Silveira, P. E. Slagboom, J. H. Smit, T. H. Sparsø, K. Stirrups, R. P. Stolk, H. M. Stringham, M. A. Swertz, A. J. Swift, A.-C. Syvänen, S.-T. Tan, B. Thorand, A. Tönjes, A. Tremblay, E. Tsafantakis, P. J. Van Der Most, U. Völker, M.-C. Vohl, J. M. Vonk, M. Waldenberger, R. W. Walker, R. Wennauer, E. Widén, G. Willemsen, T. Wilsgaard, A. F. Wright, M. C. Zillikens, S. C. Van Dijk, N. M. Van Schoor, F. W. Asselbergs, P. I. W. De Bakker, J. S. Beckmann, J. Beilby, D. A. Bennett, R. N. Bergman, S. Bergmann, C. A. Böger, B. O. Boehm, E. Boerwinkle, D. I. Boomsma, S. R. Bornstein, E. P. Bottinger, C. Bouchard, J. C. Chambers, S. J. Chanock, D. I. Chasman, F. Cucca, D. Cusi, G. Dedoussis, J. Erdmann, J. G. Eriksson, D. A. Evans, U. De Faire, M. Farrall, L. Ferrucci, I. Ford, L. Franke, P. W. Franks, P. Froguel, R. T. Gansevoort, C. Gieger, H. Grönberg, V. Gudnason, U. Gyllensten, P. Hall, A. Hamsten, P. Van Der Harst, C. Hayward, M. Heliövaara, C. Hengstenberg, A. A. Hicks, A. Hingorani, A. Hofman, F. Hu, H. V. Huikuri, K. Hveem, A. L. James, J. M. Jordan, A. Jula, M. Kähönen, E. Kajantie, S. Kathiresan, L. A. L. M. Kiemeney, M. Kivimaki, P. B. Knekt, H. A. Koistinen, J. S. Kooner, S. Koskinen, J. Kuusisto, W. Maerz, N. G. Martin, M. Laakso, T. A. Lakka, T.

Lehtimäki, G. Lettre, D. F. Levinson, L. Lind, M.-L. Lokki, P. Mäntyselkä, M. Melbye, A. Metspalu, B. D. Mitchell, F. L. Moll, J. C. Murray, A. W. Musk, M. S. Nieminen, I. Njølstad, C. Ohlsson, A. J. Oldehinkel, B. A. Oostra, L. J. Palmer, J. S. Pankow, G. Pasterkamp, N. L. Pedersen, O. Pedersen, B. W. Penninx, M. Perola, A. Peters, O. Polašek, P. P. Pramstaller, B. M. Psaty, L. Qi, T. Quertermous, O. T. Raitakari, T. Rankinen, R. Rauramaa, P. M. Ridker, J. D. Rioux, F. Rivadeneira, J. I. Rotter, I. Rudan, H. M. Den Ruijter, J. Saltevo, N. Sattar, H. Schunkert, P. E. H. Schwarz, A. R. Shuldiner, J. Sinisalo, H. Snieder, T. I. A. Sørensen, T. D. Spector, J. A. Staessen, B. Stefania, U. Thorsteinsdottir, M. Stumvoll, J.-C. Tardif, E. Tremoli, J. Tuomilehto, A. G. Uitterlinden, M. Uusitupa, A. L. M. Verbeek, S. H. Vermeulen, J. S. Viikari, V. Vitart, H. Völzke, P. Vollenweider, G. Waeber, M. Walker, H. Wallaschofski, N. J. Wareham, H. Watkins, E. Zeggini, CHARGE Consortium, DIAGRAM Consortium, GLGC Consortium, Global-BPGen Consortium, ICBP Consortium, MAGIC Consortium, A. Chakravarti, D. J. Clegg, L. A. Cupples, P. Gordon-Larsen, C. E. Jaquish, D. C. Rao, G. R. Abecasis, T. L. Assimes, I. Barroso, S. I. Berndt, M. Boehnke, P. Deloukas, C. S. Fox, L. C. Groop, D. J. Hunter, E. Ingelsson, R. C. Kaplan, M. I. McCarthy, K. L. Mohlke, J. R. O'Connell, D. Schlessinger, D. P. Strachan, K. Stefansson, C. M. Van Duijn, J. N. Hirschhorn, C. M. Lindgren, I. M. Heid, K. E. North, I. B. Borecki, Z. Kutalik, R. J. F. Loos, The influence of age and sex on genetic associations with adult body size and shape: A large-scale genome-wide interaction study. *PLOS Genet.* **11**, e1005378 (2015).

13. N. Vilor-Tejedor, M. A. Ikram, G. Roshchupkin, E. J. Vinke, M. W. Vernooij, H. H. H. Adams, Aging-dependent genetic effects associated to ADHD predict longitudinal changes of ventricular volumes in adulthood. *Front. Psychiatry* **11**, 574 (2020).

14. X. Jiang, C. Holmes, G. McVean, The impact of age on genetic risk for common diseases. *PLOS Genet.* **17**, e1009723 (2021).

15. R. M. Brouwer, M. Klein, K. L. Grasby, H. G. Schnack, N. Jahanshad, J. Teeuw, S. I. Thomopoulos, E. Sprooten, C. E. Franz, N. Gogtay, W. S. Kremen, M. S. Panizzon, L. M. Olde Loohuis, C. D. Whelan, M. Aghajani, C. Alloza, D. Alnæs, E. Artiges, R. Ayesa-Arriola, G. J. Barker, M. E. Bastin, E. Blok, E. Bøen, I. A. Breukelaar, J. K. Bright, E. E. L. Buimer, R. Bülow, D. M. Cannon, S. Ciufolini, N. A. Crossley, C. G. Damatac, P. Dazzan, C. L. de Mol, S. M. C. de Zwarte, S. Desrivières, C. M. Díaz-Caneja, N. T. Doan, K. Dohm, J. H.

Fröhner, J. Goltermann, A. Grigis, D. Grotegerd, L. K. M. Han, M. A. Harris, C. A. Hartman, S. J. Heany, W. Heindel, D. J. Heslenfeld, S. Hohmann, B. Ittermann, P. R. Jansen, J. Janssen, T. Jia, J. Jiang, C. Jockwitz, T. Karali, D. Keeser, M. G. J. C. Koevoets, R. K. Lenroot, B. Malchow, R. C. W. Mandl, V. Medel, S. Meinert, C. A. Morgan, T. W. Mühleisen, L. Nabulsi, N. Opel, V. O.-G. de la Foz, B. J. Overs, M.-L. Paillère Martinot, R. Redlich, T. R. Marques, J. Repple, G. Roberts, G. V. Roshchupkin, N. Setiaman, E. Shumskaya, F. Stein, G. Sudre, S. Takahashi, A. Thalamuthu, D. Tordesillas-Gutiérrez, A. van der Lugt, N. E. M. van Haren, J. M. Wardlaw, W. Wen, H.-J. Westeneng, K. Wittfeld, A. H. Zhu, A. Zugman, N. J. Armstrong, G. Bonfiglio, J. Bralten, S. Dalvie, G. Davies, M. Di Forti, L. Ding, G. Donohoe, A. J. Forstner, J. Gonzalez-Peñas, J. P. O. F. T. Guimaraes, G. Homuth, J.-J. Hottenga, M. J. Knol, J. B. J. Kwok, S. Le Hellard, K. A. Mather, Y. Milaneschi, D. W. Morris, M. M. Nöthen, S. Papiol, M. Rietschel, M. L. Santoro, V. M. Steen, J. L. Stein, F. Streit, R. M. Tankard, A. Teumer, D. van't Ent, D. van der Meer, K. R. van Eijk, E. Vassos, J. Vázquez-Bourgon, S. H. Witt, IMAGEN Consortium, H. H. H. Adams, I. Agartz, D. Ames, K. Amunts, O. A. Andreassen, C. Arango, T. Banaschewski, B. T. Baune, S. I. Belangero, A. L. W. Bokde, D. I. Boomsma, R. A. Bressan, H. Brodaty, J. K. Buitelaar, W. Cahn, S. Caspers, S. Cichon, B. Crespo-Facorro, S. R. Cox, U. Dannlowski, T. Elvsåshagen, T. Espeseth, P. G. Falkai, S. E. Fisher, H. Flor, J. M. Fullerton, H. Garavan, P. A. Gowland, H. J. Grabe, T. Hahn, A. Heinz, M. Hillegers, J. Hoare, P. J. Hoekstra, M. A. Ikram, A. P. Jackowski, A. Jansen, E. G. Jönsson, R. S. Kahn, T. Kircher, M. S. Korgaonkar, A. Krug, H. Lemaitre, U. F. Malt, J.-L. Martinot, C. McDonald, P. B. Mitchell, R. L. Muetzel, R. M. Murray, F. Nees, I. Nenadić, J. Oosterlaan, R. A. Ophoff, P. M. Pan, B. W. J. H. Penninx, L. Poustka, P. S. Sachdev, G. A. Salum, P. R. Schofield, G. Schumann, P. Shaw, K. Sim, M. N. Smolka, D. J. Stein, J. N. Trollor, L. H. van den Berg, J. H. Veldink, H. Walter, L. T. Westlye, R. Whelan, T. White, M. J. Wright, S. E. Medland, B. Franke, P. M. Thompson, H. E. H. Pol, Genetic variants associated with longitudinal changes in brain structure across the lifespan. *Nat. Neurosci.* **25**, 421–432 (2022).

16. Y. Cao, S. S. Rajan, P. Wei, Mendelian randomization analysis of a time-varying exposure for binary disease outcomes using functional data analysis methods. *Genet. Epidemiol.* **40**, 744–755 (2016).

17. E. Sanderson, T. G. Richardson, T. T. Morris, K. Tilling, G. Davey Smith, Estimation of causal effects of a time-varying exposure at multiple time points through multivariable mendelian randomization. *PLOS Genet.* **18**, e1010290 (2022).

18. T. G. Richardson, E. Sanderson, B. Elsworth, K. Tilling, G. Davey Smith, Use of genetic variation to separate the effects of early and later life adiposity on disease risk: Mendelian randomisation study. *BMJ* **369**, m1203 (2020).

19. H. Tian, S. Burgess, Estimation of time-varying causal effects with multivariable Mendelian randomization: Some cautionary notes. *Int. J. Epidemiol.* **52**, 846–857 (2023).

20. J. Shi, S. A. Swanson, P. Kraft, B. Rosner, I. De Vivo, M. A. Hernán, Mendelian randomization with repeated measures of a time-varying exposure: An application of structural mean models. *Epidemiology* **33**, 84–94 (2022).

21. J. Shi, S. A. Swanson, P. Kraft, B. Rosner, I. De Vivo, M. A. Hernán, Instrumental variable estimation for a time-varying treatment and a time-to-event outcome via structural nested cumulative failure time models. *BMC Med. Res. Methodol.* **21**, 258 (2021).

22. O. O. Aalen, A linear regression model for the analysis of life times. *Stat. Med.* **8**, 907–925 (1989).

23. T. T. Morris, J. Heron, E. C. M. Sanderson, G. Davey Smith, V. Didelez, K. Tilling, Interpretation of Mendelian randomization using a single measure of an exposure that varies over time. *Int. J. Epidemiol.* **51**, 1899–1909 (2022).

24. S. Burgess, S. G. Thompson, Use of allele scores as instrumental variables for Mendelian randomization. *Int. J. Epidemiol.* **42**, 1134–1144 (2013).

25. G. Hemani, K. Tilling, G. Davey Smith, Orienting the causal relationship between imprecisely measured traits using GWAS summary data. *PLOS Genet.* **13**, e1007081 (2017).

26. R. Chou, T. Dana, I. Blazina, M. Daeges, T. L. Jeanne, Statins for prevention of cardiovascular disease in adults: Evidence report and systematic review for the US preventive services task force. *JAMA* **316**, 2008–2024 (2016).

27. P. Verdecchia, F. Angeli, G. Reboldi, Hypertension and atrial fibrillation: Doubts and certainties from basic and clinical studies. *Circ. Res.* **122**, 352–368 (2018).

28. G. Georgiopoulos, G. Ntritsos, K. Stamatelopoulos, C. Tsioufis, A. Aimo, S. Masi, E. Evangelou, The relationship between blood pressure and risk of atrial fibrillation: A Mendelian randomization study. *Eur. J. Prev. Cardiol.* **29**, 1494–1500 (2022).

29. K. Rahimi, J. Emberson, P. McGale, W. Majoni, A. Merhi, F. W. Asselbergs, V. Krane, P. W. Macfarlane, PROSPER Executive, Effect of statins on atrial fibrillation: Collaborative meta-analysis of published and unpublished evidence from randomised controlled trials. *BMJ* **342**, d1250–d1250 (2011).

30. F. Mauvais-Jarvis, J. E. Manson, J. C. Stevenson, V. A. Fonseca, Menopausal hormone therapy and type 2 diabetes prevention: Evidence, mechanisms, and clinical implications. *Endocr. Rev.* **38**, 173–188 (2017).

31. T. Schoeler, D. Speed, E. Porcu, N. Pirastu, J.-B. Pingault, Z. Kutalik, Participation bias in the UK Biobank distorts genetic associations and downstream analyses. *Nat. Hum. Behav.* **7**, 1216–1227 (2023).

32. F. L. J. Visseren, F. Mach, Y. M. Smulders, D. Carballo, K. C. Koskinas, M. Bäck, A. Benetos, A. Biffi, J.-M. Boavida, D. Capodanno, B. Cosyns, C. Crawford, C. H. Davos, I. Desormais, E. Di Angelantonio, O. H. Franco, S. Halvorsen, F. D. R. Hobbs, M. Hollander, E. A. Jankowska, M. Michal, S. Sacco, N. Sattar, L. Tokgozoglu, S. Tonstad, K. P. Tsioufis, I. Van Dis, I. C. Van Gelder, C. Wanner, B. Williams, ESC Scientific Document Group, G. De Backer, V. Regitz-Zagrosek, A. H. Aamodt, M. Abdelhamid, V. Aboyans, C. Albus, R. Asteggiano, M. Bäck, M. A. Borger, C. Brotons, J. Čelutkienė, R. Cifkova, M. Cikes, F. Cosentino, N. Dagres, T. De Backer, D. De Bacquer, V. Delgado, H. Den Ruijter, P. Dendale, H. Drexel, V. Falk, L. Fauchier, B. A. Ference, J. Ferrières, M. Ferrini, M. Fisher, D. Fliser, Z. Fras, D. Gaita, S. Giampaoli, S. Gielen, I. Graham, C. Jennings, T. Jorgensen, A. Kautzky-Willer, M. Kavousi, W. Koenig, A. Konradi, D. Kotecha, U. Landmesser, M. Lettino, B. S. Lewis, A. Linhart, M.-L. Løchen, K. Makrilakis, G. Mancia, P. Marques-Vidal, J. W. McEvoy, P. McGreavy, B. Merkely, L. Neubeck, J. C. Nielsen, J. Perk, S. E. Petersen, A. S. Petronio, M. Piepoli, N. G. Pogosova, E. I. B. Prescott, K. K. Ray, Z. Reiner, D. J. Richter, L.

Rydén, E. Shlyakhto, M. Sitges, M. Sousa-Uva, I. Sudano, M. Tiberi, R. M. Touyz, A. Ungar, W. M. M. Verschuren, O. Wiklund, D. Wood, J. L. Zamorano, Y. M. Smulders, D. Carballo, K. C. Koskinas, M. Bäck, A. Benetos, A. Biffi, J.-M. Boavida, D. Capodanno, B. Cosyns, C. A. Crawford, C. H. Davos, I. Desormais, E. Di Angelantonio, O. H. Franco Duran, S. Halvorsen, F. D. Richard Hobbs, M. Hollander, E. A. Jankowska, M. Michal, S. Sacco, N. Sattar, L. Tokgozoglu, S. Tonstad, K. P. Tsioufis, I. V. Dis, I. C. Van Gelder, C. Wanner, B. Williams, 2021 ESC Guidelines on cardiovascular disease prevention in clinical practice. *Eur. Heart J.* **42**, 3227–3337 (2021).

33. F. Mach, C. Baigent, A. L. Catapano, K. C. Koskinas, M. Casula, L. Badimon, M. J. Chapman, G. G. De Backer, V. Delgado, B. A. Ference, I. M. Graham, A. Halliday, U. Landmesser, B. Mihaylova, T. R. Pedersen, G. Riccardi, D. J. Richter, M. S. Sabatine, M.-R. Taskinen, L. Tokgozoglu, O. Wiklund, ESC Scientific Document Group, 2019 ESC/EAS Guidelines for the management of dyslipidaemias: Lipid modification to reduce cardiovascular risk. *Eur. Heart J.* **41**, 111–188 (2020).

34. M. Brunström, B. Carlberg, Association of blood pressure lowering with mortality and cardiovascular disease across blood pressure levels: A systematic review and meta-analysis. *JAMA Intern. Med.* **178**, 28–36 (2018).

35. L. Mosca, E. Barrett-Connor, N. Kass Wenger, Sex/gender differences in cardiovascular disease prevention: What a difference a decade makes. *Circulation* **124**, 2145–2154 (2011).

36. J. Bowden, M. V. Holmes, Meta-analysis and Mendelian randomization: A review. *Res. Synth. Methods* **10**, 486–496 (2019).

37. H. Yaghootkar, L. A. Lotta, J. Tyrrell, R. A. J. Smit, S. E. Jones, L. Donnelly, R. Beaumont, A. Campbell, M. A. Tuke, C. Hayward, K. S. Ruth, S. Padmanabhan, J. W. Jukema, C. C. Palmer, A. Hattersley, R. M. Freathy, C. Langenberg, N. J. Wareham, A. R. Wood, A. Murray, M. N. Weedon, N. Sattar, E. Pearson, R. A. Scott, T. M. Frayling, Genetic evidence for a link between favorable adiposity and lower risk of type 2 diabetes, hypertension, and heart disease. *Diabetes* **65**, 2448–2460 (2016).

38. S. Burgess, J. Bowden, T. Fall, E. Ingelsson, S. G. Thompson, Sensitivity analyses for robust causal inference from mendelian randomization analyses with multiple genetic variants. *Epidemiology* **28**, 30–42 (2017).

39. S. Martin, M. Cule, N. Basty, J. Tyrrell, R. N. Beaumont, A. R. Wood, T. M. Frayling, E. Sorokin, B. Whitcher, Y. Liu, J. D. Bell, E. L. Thomas, H. Yaghootkar, Genetic evidence for different adiposity phenotypes and their opposing influences on ectopic fat and risk of cardiometabolic disease. *Diabetes* **70**, 1843–1856 (2021).

40. C. N. Foley, A. M. Mason, P. D. W. Kirk, S. Burgess, MR-Clust: Clustering of genetic variants in Mendelian randomization with similar causal estimates. *Bioinformatics* **37**, 531–541 (2021).

41. D. E. Coral, J. Fernandez-Tajes, N. Tsereteli, H. Pomares-Millan, H. Fitipaldi, P. M. Mutie, N. Atabaki-Pasdar, S. Kalamajski, A. Poveda, T. W. Miller-Fleming, X. Zhong, G. N. Giordano, E. R. Pearson, N. J. Cox, P. W. Franks, A phenome-wide comparative analysis of genetic discordance between obesity and type 2 diabetes. *Nat. Metab.* **5**, 237–247 (2023).

42. H. Tian, A. Patel, S. Burgess, Estimating time-varying exposure effects through continuous-time modelling in mendelian randomization. *Stat. Med.* **43**, 5166–5181 (2024).

43. D. J. P. Barker, C. Osmond, Infant mortality, childhood nutrition, and ischaemic heart disease in England and Wales. *Lancet* **327**, 1077–1081 (1986).

44. N. B. Lister, L. A. Baur, J. F. Felix, A. J. Hill, C. Marcus, T. Reinehr, C. Summerbell, M. Wabitsch, Child and adolescent obesity. *Nat. Rev. Dis. Primers.* **9**, 24 (2023).

45. C. Sudlow, J. Gallacher, N. Allen, V. Beral, P. Burton, J. Danesh, P. Downey, P. Elliott, J. Green, M. Landray, B. Liu, P. Matthews, G. Ong, J. Pell, A. Silman, A. Young, T. Sprosen, T. Peakman, R. Collins, UK Biobank: An open access resource for identifying the causes of a wide range of complex diseases of middle and old age. *PLOS Med.* **12**, e1001779 (2015).

46. UK10K Consortium, K. Walter, J. L. Min, J. Huang, L. Crooks, Y. Memari, S. McCarthy, J. R. B. Perry, C. Xu, M. Futema, D. Lawson, V. Iotchkova, S. Schiffels, A. E. Hendricks, P. Danecek, R. Li, J. Floyd, L. V. Wain, I. Barroso, S. E. Humphries, M. E. Hurles, E. Zeggini,

J. C. Barrett, V. Plagnol, J. B. Richards, C. M. T. Greenwood, N. J. Timpson, R. Durbin, N. Soranzo, The UK10K project identifies rare variants in health and disease. *Nature* **526**, 82–90 (2015).

47.1000G enomes Project Consortium, A. Auton, L. D. Brooks, R. M. Durbin, E. P. Garrison, H. M. Kang, J. O. Korbel, J. L. Marchini, S. M. Carthy, G. A. McVean, G. R. Abecasis, A global reference for human genetic variation. *Nature* **526**, 68–74 (2015).

48. S. Purcell, B. Neale, K. Todd-Brown, L. Thomas, M. A. R. Ferreira, D. Bender, J. Maller, P. Sklar, P. I. W. De Bakker, M. J. Daly, P. C. Sham, PLINK: A tool set for whole-genome association and population-based linkage analyses. *Am. J. Hum. Genet.* **81**, 559–575 (2007).

49. X. Meng, R. Rosenthal, D. B. Rubin, Comparing correlated correlation coefficients. *Psychol. Bull.* **111**, 172–175 (1992).

50. Y. Benjamini, Y. Hochberg, Controlling the false discovery rate: A practical and powerful approach to multiple testing. *J. R. Stat. Soc. B. Methodol.* **57**, 289–300 (1995).

51. S. Burgess, N. M. Davies, S. G. Thompson, Bias due to participant overlap in two-sample Mendelian randomization. *Genet. Epidemiol.* **40**, 597–608 (2016).

52. O. Aalen, "A model for nonparametric regression analysis of counting processes," in *Mathematical Statistics and Probability Theory*, vol. 2 of *Lecture Notes in Statistics*, W. Klonecki, A. Kozek, J. Rosiński, Eds. (Springer New York, 1980), pp. 1–25; http://link.springer.com/10.1007/978-1-4615-7397-5_1.

53. O. O. Aalen, R. J. Cook, K. Røysland, Does Cox analysis of a randomized survival study yield a causal treatment effect? *Lifetime Data Anal.* **21**, 579–593 (2015).

54. T. Martinussen, S. Vansteelandt, On collapsibility and confounding bias in Cox and Aalen regression models. *Lifetime Data Anal.* **19**, 279–296 (2013).

55. A. Sjölander, E. Dahlqwist, J. Zetterqvist, A note on the noncollapsibility of rate differences and rate ratios. *Epidemiology* **27**, 356–359 (2016).

56. X. Liang, N. Mounier, N. Apfel, S. Khalid, T. M. Frayling, J. Bowden, Using clustering of genetic variants in Mendelian randomization to interrogate the causal pathways underlying multimorbidity from a common risk factor. *Genet. Epidemiol.* **49**, e22582 (2025).

57. A. Mahajan, D. Taliun, M. Thurner, N. R. Robertson, J. M. Torres, N. W. Rayner, A. J. Payne, V. Steinthorsdottir, R. A. Scott, N. Grarup, J. P. Cook, E. M. Schmidt, M. Wuttke, C. Sarnowski, R. Mägi, J. Nano, C. Gieger, S. Trompet, C. Lecoeur, M. H. Preuss, B. P. Prins, X. Guo, L. F. Bielak, J. E. Below, D. W. Bowden, J. C. Chambers, Y. J. Kim, M. C. Y. Ng, L. E. Petty, X. Sim, W. Zhang, A. J. Bennett, J. Bork-Jensen, C. M. Brummett, M. Canouil, K.-U. Ec Kardt, K. Fischer, S. L. R. Kardia, F. Kronenberg, K. Läll, C.-T. Liu, A. E. Locke, J. Luan, I. Ntalla, V. Nylander, S. Schönherr, C. Schurmann, L. Yengo, E. P. Bottinger, I. Brandslund, C. Christensen, G. Dedoussis, J. C. Florez, I. Ford, O. H. Franco, T. M. Frayling, V. Giedraitis, S. Hackinger, A. T. Hattersley, C. Herder, M. A. Ikram, M. Ingelsson, M. E. Jørgensen, T. Jørgensen, J. Kriebel, J. Kuusisto, S. Ligthart, C. M. Lindgren, A. Linneberg, V. Lyssenko, V. Mamakou, T. Meitinger, K. L. Mohlke, A. D. Morris, G. Nadkarni, J. S. Pankow, A. Peters, N. Sattar, A. Stančáková, K. Strauch, K. D. Taylor, B. Thorand, G. Thorleifsson, U. Thorsteinsdottir, J. Tuomilehto, D. R. Witte, J. Dupuis, P. A. Peyser, E. Zeggini, R. J. F. Loos, P. Froguel, E. Ingelsson, L. Lind, L. Groop, M. Laakso, F. S. Collins, J. W. Jukema, C. N. A. Palmer, H. Grallert, A. Metspalu, A. Dehghan, A. Köttgen, G. R. Abecasis, J. B. Meigs, J. I. Rotter, J. Marchini, O. Pedersen, T. Hansen, C. Langenberg, N. J. Wareham, K. Stefansson, A. L. Gloyn, A. P. Morris, M. Boehnke, M. I. McCarthy, Fine-mapping type 2 diabetes loci to single-variant resolution using high-density imputation and islet-specific epigenome maps. *Nat. Genet.* **50**, 1505–1513 (2018).

58. P. Van Der Harst, N. Verweij, Identification of 64 novel genetic loci provides an expanded view on the genetic architecture of coronary artery disease. *Circ. Res.* **122**, 433–443 (2018).

59. V. Didelez, N. Sheehan, Mendelian randomization as an instrumental variable approach to causal inference. *Stat. Methods Med. Res.* **16**, 309–330 (2007).

60. M. A. Hernán, Robins, James M., *Causal Inference: What If* (Chapman & Hall/CRC, 2024).
